# GStream: Improving SNP and CNV Coverage on Genome-Wide Association Studies

**DOI:** 10.1371/journal.pone.0068822

**Published:** 2013-07-03

**Authors:** Arnald Alonso, Sara Marsal, Raül Tortosa, Oriol Canela-Xandri, Antonio Julià

**Affiliations:** 1 Rheumatology Research Group, Vall d'Hebron Hospital Research Institute, Barcelona, Spain; 2 Department of ESAII, Polytechnical University of Catalonia, Barcelona, Spain; The Scripps Research Institute, United States of America

## Abstract

We present GStream, a method that combines genome-wide SNP and CNV genotyping in the Illumina microarray platform with unprecedented accuracy. This new method outperforms previous well-established SNP genotyping software. More importantly, the CNV calling algorithm of GStream dramatically improves the results obtained by previous state-of-the-art methods and yields an accuracy that is close to that obtained by purely CNV-oriented technologies like Comparative Genomic Hybridization (CGH). We demonstrate the superior performance of GStream using microarray data generated from HapMap samples. Using the reference CNV calls generated by the 1000 Genomes Project (1KGP) and well-known studies on whole genome CNV characterization based either on CGH or genotyping microarray technologies, we show that GStream can increase the number of reliably detected variants up to 25% compared to previously developed methods. Furthermore, the increased genome coverage provided by GStream allows the discovery of CNVs in close linkage disequilibrium with SNPs, previously associated with disease risk in published Genome-Wide Association Studies (GWAS). These results could provide important insights into the biological mechanism underlying the detected disease risk association. With GStream, large-scale GWAS will not only benefit from the combined genotyping of SNPs and CNVs at an unprecedented accuracy, but will also take advantage of the computational efficiency of the method.

## Introduction

Over the last years, Genome-Wide Association Studies (GWAS) using microarray-based technology have played an important role in the identification of common genetic variations and their relationship with disease susceptibility [1], [2], [3], [4]. Genotyping microarrays [5] have enabled this success through the parallel genotyping of thousands of Single Nucleotide Polymorphisms (SNPs), capturing most of the common variation in the human genome. Very recently, the new generation of microarrays have integrated the extensive knowledge revealed by the 1KGP [6] and, together with the decreasing costs of this technology, are now allowing the use of the GWAS approach to the association of rare genetic risk variants or more complex human traits.

In addition to SNPs, Copy Number Variants (CNVs) have shown to play an important role in disease susceptibility [7]. CNVs are relatively large (>500 bp) genomic variations and include deletions, tandem duplications and insertions [8]. Recent studies based either on specific CGH arrays or genotyping microarrays have demonstrated the importance of CNVs due to their global contribution to the human genome variation, their functional impact and their role in human disease [7], [9], [10], [11], [12], [13], [14]. Some of these reference studies have contributed to elaborate a map of regions containing highly polymorphic CNVs called Copy Number Polymorphisms (CNPs) [9], [10], [15]. These common variations have appeared as a significant area of interest, since they segregate in the population at an appreciable frequency and their analysis over big sample collections could potentially lead to significant disease risk associations.

The development of the two mentioned technologies (CGH arrays and genotyping microarrays) for high throughput CNV screening has prompted the inclusion of CNVs in GWAS studies [16], [17], [18], [19]. When comparing both technologies, genotyping microarrays offer the practical advantage of obtaining at the same time SNP and CNV genotype data. However, there is still a major need to develop methods that can best deal with the signal-to-noise ratio deficiencies and genomic coverage of genotyping microarray data when attempting to identify and quantify CNVs. So far, most of the commonly used methods for CNV detection and genotyping at the genome-wide scale [20], [21], [22] are based on independent per-sample analyses that use summarized measurements relative to a reference set of samples. This type of approach has proven to work well for large genomic variations, but it fails to use the powerful information generated by analyzing multiple samples, leading to high false negative rates with small CNVs [23].

In this study we present GStream, a method for SNP and CNV/CNP genotyping that is tailored to GWAS objectives. GStream integrates a substantially improved version of our previous CNV calling software CNstream [24]. Our new method achieves a superior accuracy in both SNP and CNV genotyping compared to well-established methods. Indeed, GStream obtains an unprecedented accuracy within CNV regions, with a performance close to that obtained from purely CNV-oriented technologies like CGH arrays. All these improvements have been quantitatively compared against previous state-of-the-art methods and accurately assessed using different Illumina genotyping microarrays together with publicly available SNP [25] and CNV [9], [10], [15] reference datasets based on Next-Generation Sequencing (NGS), CGH array and genotyping microarray technologies. Finally, the computational efficiency of the method has been optimized, enabling the large-scale SNP and CNV analyses to be performed in a short amount of time.

In addition to presenting this new method and demonstrating its superior performance over reference datasets, we have also performed different relational analyses concerning previously known risk loci. Using GStream we have been able to identify, for the first time, several CNVs in strong linkage disequilibrium (LD) with risk-associated SNPs [26] as well as CNVs spanning disease-associated genes [27]. Together, these results could reveal important insights into the causality of these disease risk associations.

## Materials and Methods

We first introduce the Illumina BeadChip microarrays and describe the algorithms for SNP and CNV genotyping. Next, we provide information about the datasets used in this study and the implemented metrics for evaluating SNP and CNV genotyping accuracy. Finally, we describe the methods used for the CNV association studies that we have run using the GWAS catalog [26] and the OMIM [27] databases.

### Illumina BeadChip Data

Illumina BeadChips have been largely used in large-scale genome-wide association studies and are based on the Infinium assay technology [28]. This type of genotyping array consists on hundreds of thousands of probe pairs designed to capture genomic variation at the SNP and CNV level. In each probe pair, each probe has been designed to specifically bind one of the two SNP alleles (i.e. alleles A and B) generating a pair of fluorescence intensities. These signals are then measured and processed in order to infer the presence or absence of these alleles in each sample. GStream software uses these raw measurements to extract SNP and CNV genotypes for each sample at each probe pair. From here on, fluorescence measurements of alleles A and B will be called channel A and B intensities and samples will be categorized at each SNP as homozygotes (i.e. AA or BB) or heterozygotes (i.e. AB).

### GStream method for SNP genotyping

**Figure 1:**
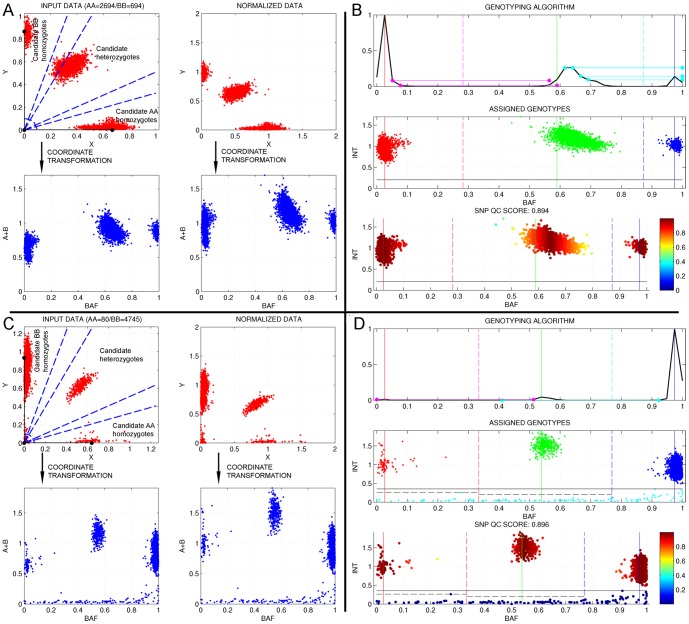
GStream method for SNP genotyping. This figure shows how GStream genotyping method works on two example markers, the first one representing a typical marker capturing a SNP (A and B) and the second one capturing both a SNP and a CNV (C and D). The leftmost graphs show the effects of the normalization procedure for the two markers, where the dotted blue lines enclose the ranges where candidate homozygotes and heterozygotes are identified in order to compute the scaling factors for each channel (black points over the axes). The rightmost graphs give an overview of the genotyping procedure: Upper subfigures represent the scaled BAF probability density function with the solid vertical lines setting the identified genotype centres, the dotted vertical lines setting the genotype limits and the horizontal lines representing the sequential search of genotype cluster peaks. Medium and lower subfigures represent genotype calls and quality call scores respectively.

Before identifying the clusters corresponding to the AA, AB and BB genotypes at each probe, raw intensity data of each probe must be normalized in order to equalize the overall sample intensity distribution at each channel (Figure 1). This step is crucial since the sensitivity differences of each probe and channel can lead to bias affecting the genotyping performance. The method used by GStream is based on the scaling correction used by Peiffer et al. [29]. In this method, the intensity centroids of a set of pre-computed AA and BB candidate homozygote samples are identified and used to scale channel A and B intensities. However, GStream adds two modifications in order to improve the normalization in those cases involving probes capturing both SNP and CNV variation. First, instead of using candidate homozygote intensity centroids, the scaling parameter is computed by weighting the candidate homozygotes intensity distributions (the higher the intensity, the higher the weight) and by finding the maximum over the resulting curve (Figure S1). This modification helps GStream to better deal with the particularities of intensity distributions coming from probes within CNV regions. The second modification introduced by GStream uses heterozygote intensity data when no homozygote candidates are found, thus helping to better deal with probes capturing low MAF SNPs.

Once the intensities from both A and B channels have been normalized, GStream proceeds to identify the clusters corresponding to each SNP genotype (i.e. AA, AB and BB). Developing an accurate SNP genotyping method is crucial not only for SNP analysis itself, but also because GStream CNV genotyping method uses this information to improve the accuracy of the CNV call. GStream applies the following procedure to assign a SNP genotype to each sample at each marker:

1. Channel A and channel B intensities from the analyzed marker are transformed to B allele frequencies (BAF) and absolute intensities [29].
2. Absolute intensities are used to detect samples without any allele (homozygous deletions) which are characterized by very low intensities at both channels. In order to compute the absolute intensity threshold between homozygous deletion samples and the other samples, the absolute intensities are sorted and then differences between each pair of consecutive intensities are computed. If high intensity differences are found within the expected threshold range ([0, 0.5]), the zero-threshold is fixed to the corresponding intensities (Figure S2).
3. The BAF probability density function (PDF) is estimated by computing the scaled histogram of all the sample BAF values. Peaks over this PDF corresponding to genotype clusters will be identified sequentially starting by the peak generated by the major allele frequency homozygote cluster. The algorithm establishes a minimum separation between peaks in order to assign them to different genotype clusters and it stops when three peaks have been found or when no more peaks are found. Once genotype peaks have been found, genotype limits are computed by finding the PDF minimum between each consecutive pair of centres (Figure 1). These limits will define the BAF intervals assigned to each genotype and each sample will be genotyped accordingly to them.
4. If the number of genotype peaks identified is less than three, each genotype cluster is re-analyzed with a better resolution (i.e. increasing the number of histogram bins to estimate the BAF PDF) in order to identify sub-clusters corresponding to different genotypes. This procedure avoids common errors seen in others algorithms where, for example, genotypes of SNPs with highly discordant sensitivities at each channel are incorrectly assigned.
5. Finally, a global genotyping quality score and an individual score for each sample genotype are computed (Figure 1). The global score is proportional to the standard deviation mean of the BAF values assigned to each genotype and the individual score corresponds to the distance between the sample BAF value and its corresponding genotype peak divided by the distance between genotype centres.

Both genotypes and quality control measurements for each sample are extracted by GStream to the output files. This information is also required by the CNV genotyping method, which is based both on the normalized channel intensities and the SNP genotype information. Further algorithm details are given in Text S1.

### GStream method for CNV genotyping

CNV identification and genotyping is one of the principal contributions of GStream to the current state-of-the-art microarray genotyping methodology, clearly outperforming previous approaches. Although this method has been based on our previous CNstream method [24], multiple changes have been introduced in order to improve performance and computational efficiency.

GStream uses normalized intensities and SNP genotypes computed in the SNP genotyping stage to identify the presence of deletions and amplifications characterized by variable clustering patterns on the intensity data (i.e. high frequency CNVs) or by slight deviations from the diploid distribution (i.e. low frequency CNVs).

**Figure 2:**
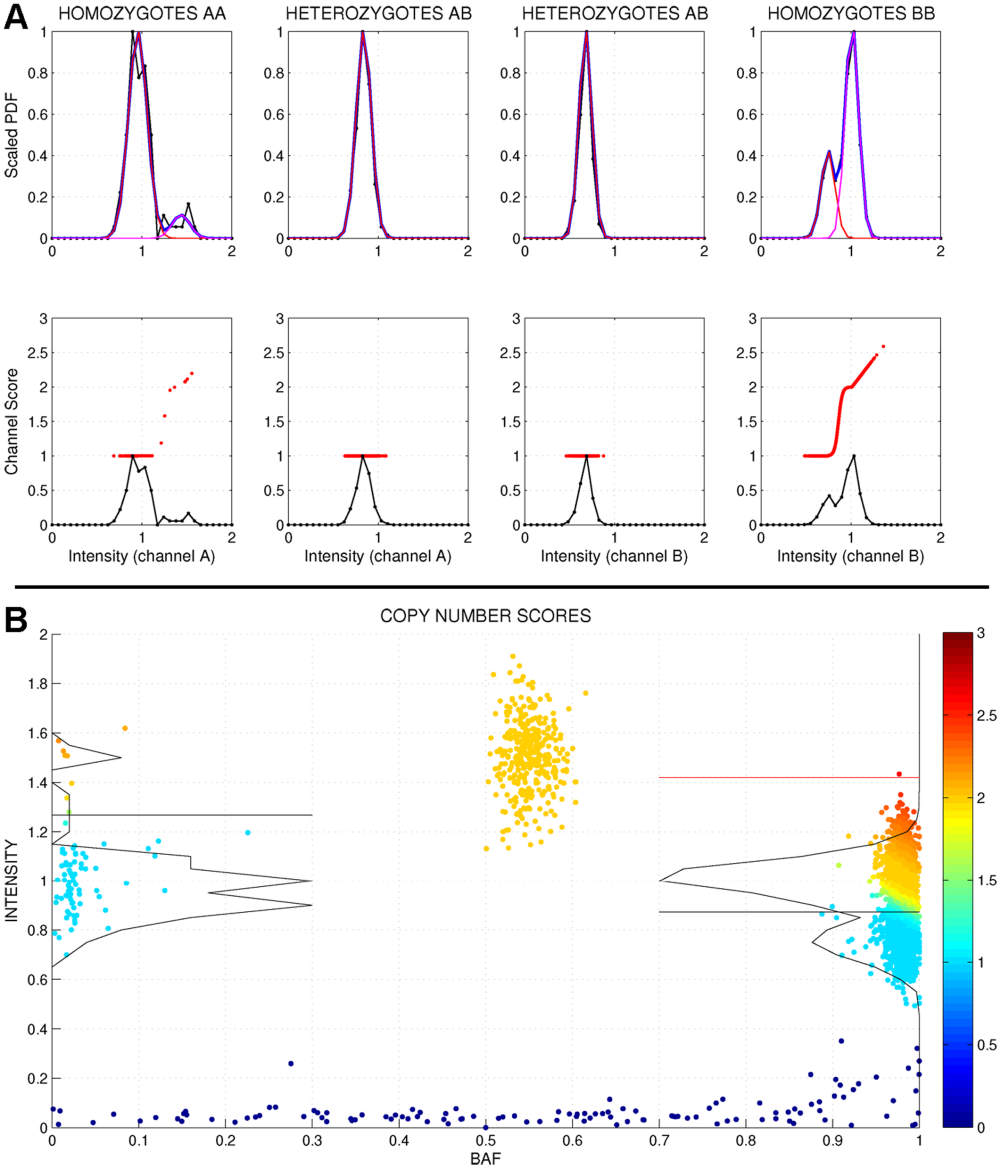
GStream method for CNV genotyping. (A) Each CNV analysis is divided in four independent sets where the number of allele copies per channel intensity is estimated. Here, the homozygote intensities over its respective informative channels (upper rightmost and leftmost graphs) are fitted with a two-component model (in this case, capturing a deletion) while heterozygote intensities over each channel are better fitted with a one-component model (upper centre graphs). Lower graphs show the intensity distributions (solid black lines) together with the corresponding copy number score (red points) assigned to each sample. AA homozygotes are mostly classified as deletions (scores near to 1), BB homozygotes are divided into diploids (scores∼2) and deletions (scores∼1) while heterozygotes are classified as diploids (i.e. one allele detected at each channel). (B) Final representation of the analyzed probe where points represent samples and colour their relative copy number scores. SNP and CNV genotypes are assigned along the BAF and the intensity axis respectively.

One of the improvements incorporated in the algorithm is that each SNP genotype cluster is independently analyzed taking only into account the intensity channel that carries valuable information. This way, the CNV algorithm is divided in four parallel steps (Figure 2A):

- Analysis of channel A intensities from the samples previously genotyped as AA homozygotes.
- Analysis of channel B intensities from the samples previously genotyped as BB homozygotes.
- Analysis of channel A intensities from the samples previously genotyped as heterozygotes (i.e. AB).
- Analysis of channel B intensities from the samples previously genotyped as heterozygotes (i.e. AB).

As well as dividing the analysis in four independent steps, the algorithm is based on the following assumptions:

- Homozygous deletions (0 allele copies) are previously detected during the SNP genotyping stage.
- Due to the technical limitations of genotyping microarrays, the intensity measurements show a saturation effect when amplifications are found. For this reason, intensity clustering patterns corresponding to amplifications are very rare and hard to detect unless they span multiple probes [30].
- Samples categorized as homozygote samples (i.e. AA and BB) can correspond to hemizygous deletions (i.e. A and B) or amplifications (i.e. AA+ and BB+). Due to the saturation effect the algorithm does not stratify amplifications by the number of allele copies.
- Samples characterized as heterozygotes (i.e. AB) can have two or more copies (i.e. AB, AAB, ABB …). The total number of copies can be inferred by independently computing the number of copies of each allele and then adding the results for each sample.

Below we describe the procedure for determining the CNV genotypes from the set of channel intensities of each one of the four analysis steps.

#### Model selection

For each SNP genotype, the algorithm starts identifying clusters over the channel intensities that carry the corresponding allele information (i.e. channel A for AA homozygotes, channel B for BB homozygotes and both channels for AB heterozygotes). Due to the mentioned saturation effects, it is very uncommon to observe more than two intensity clusters in microarray data and, for this reason, only two models will be fitted to the intensity data: a one- and a two-component Gaussian mixture model (GMM). The first one will be fitted using the mean and the variance of all the intensities while the second one will be fitted using the Expectation-Maximization algorithm [31] (Figure 2A). A set of requirements in order to select the second model have been carefully developed and only if all of them are accomplished, the two-component model (indicating a pattern corresponding to a common CNV) will be selected (Figure 2A).

#### Component labeling

If the two-component model has been selected, a copy number category will be assigned to each one of the two components. As no prior knowledge is available to assign the two components either to a deletion pattern (i.e. CN = 1 and CN = 2) or to an amplification pattern (i.e. CN = 2 and CN = 3), a disambiguation method is necessary. GStream bases the component labelling both on the relative weight of each component (i.e. proportional to the copy number frequency) and on the presence of homozygous deletions (Figure S3A). When the one-component model has been selected, the component will be labelled by default to CN = 2 (i.e. diploid), which is assumed to be the most common state.

#### Outlier identification and CNV scoring

Outlier identification is intended to capture low frequency CNVs that are not captured by a two-component GMM and is based on identifying samples showing high or low deviations from the intensity distributions defined by the selected model. CNV scoring assigns to each sample *i* a score *S* between 0 and 3 depending on its copy number posterior probabilities (Figure S3B). At the end of this step, GStream has obtained a CNV score for all the samples that allows the identification of deletions and amplifications as well as a quantification of the reliability of the assignment (Figure 2B). Additional algorithm details are given in Text S1.

### Microarray data from HapMap samples

In order to evaluate and compare the performance of GStream SNP and CNV genotyping methods we have used raw Illumina microarray data from HapMap samples available at the Gene Expression Omnibus (GEO, http://www.ncbi.nlm.nih.gov/geo/) [32] (Table 1). This data has been also used as the input of state-of-the-art SNP and CNV genotyping algorithms in order to extract accurate comparison measures as well as to ensure a performance assessment independent from the technical biases of the raw data. Only markers having available NCBI Build 37 mapping information were kept for further analysis.

**Table 1:** Public microarray data used in this study.

PLATFORM	POPULATION	SAMPLES	GEO ACCESSION	AUTOSOMAL MARKERS	EVALUATION
Human610-Quad	CEU/CHBJPT/YRI	73/75/77	GSE17205/GSE17206/GSE17207	596528	568182
Human660W-Quad	CEU/CHBJPT/YRI	89/89/89	GSE17208/GSE17209/GSE17210	638582	552529
Human1M-Duo	CEU/CHBJPT/YRI	89/90/90	GSE16894/GSE16895/GSE16896	1141594	1058827
HumanOmni1-Quad	CEU/CHBJPT/YRI	88/89/90	GSE17197/GSE17201/GSE17203	1103791	882445

The used microarray data comes from four different Illumina BeadChip platforms and the sample data comes from three HapMap populations. The total number of autosomal markers and the number of markers used for SNP genotyping evaluation are shown.

### SNP genotyping performance evaluation and comparison with previous methods

#### Golden standard genotypes

In order to correctly evaluate the SNP genotyping algorithm performance, a set of independent and high-quality genotype calls is required. The genotype calls of HapMap samples have been established as a golden standard commonly used in the literature for performance evaluation of SNP genotyping methods. These calls are available for download through the online HapMap tool HapMart (http://hapmap.ncbi.nlm.nih.gov/biomart). For this study, we downloaded the genotypes corresponding to the samples having available microarray data and used them as the golden standard. SNPs used for performance evaluation were chosen in order to fulfil three criteria: (i) to have available Build37 mapping information, (ii) to be present both in the analyzed microarray platform and in the golden standard HapMap dataset, and (iii) to have concordant reference alleles both in the microarray and in the golden standard annotations (Table 1).

#### Algorithms

GStream SNP genotyping accuracy has been evaluated and compared with three methods: (i) GenoSNP [33] which is a well-known genotyping algorithm based on a within-sample approach; (ii) GenCall, which is the proprietary (Illumina, San Diego, US) algorithm [34], and it is used by the vendor genotyping software; (iii) M3 [35], which is a recently published method for SNP genotyping that re-analyzes the data in order to increase the accuracy over the low MAF SNPs and has shown to have increased performance.

### Copy number genotyping performance evaluation

Copy number evaluation was performed at two levels: Evaluation of the GStream ability to detect structural variation obtained from the 1000 Genomes Project [6] next-generation sequencing (NGS) data and evaluation and comparison of CNV population association results using different algorithms and golden standard calls from three recently published studies [9], [10], [15]. Below we describe materials, methods and metrics used for this two-stage evaluation.

#### Evaluation of CNV genotyping accuracy over the 1KGP Structural Variants

In order to test the ability of GStream to detect copy number variation, we have used the HumanOmni1-Quad GStream calls and a golden standard dataset from a public release of the 1KGP. HumanOmni1-Quad platform was chosen due to its highest coverage and resolution which allowed an evaluation over a major number of loci. The golden standard dataset consisted of the last variant call files that have been released by the 1KGP (version v3_20110521, ftp://ftp.1000genomes.ebi.ac.uk/vol1/ftp/release). From all the variants included in the 1KGP release we chose those that corresponded to structural variations (i.e. CNVs) and we filtered out variants with a MAF lower than 2% within all the populations. The resulting set included 2,531 structural variants (SV) with their respective calls over N = 353 unrelated HapMap samples. From these 2,531 loci, 1,956 are covered by HumanOmni1-Quad markers and GStream calls were available for 149 out of the 353 1KGP samples, which jointly formed the final evaluation set.

The evaluation procedure consisted of finding the markers whose GStream calls are in maximum LD with each SV:
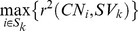
where S_k_ is the set of microarray markers within the region spanned by the SV, CN_i_ is the copy number genotypes assigned by GStream at marker i and SV_k_ is the 1KGP calls for the analyzed SV *k*.

#### Evaluation of the power to detect genome-wide associations over CNV markers and comparison with previous methods

The objective of this section is to evaluate the power to detect CNV associations and to compare GStream performance with other well-known methods. This comparison was performed using Human1M-Duo and HumanOmni1-Quad platforms. Using these two platforms also allowed an assessment of the specific platform power to detect CNV associations, comparing platforms with (i.e. HumanOmni1) and without (i.e. Human1M) a specific design to cover CNV.

The genome-wide association study over CNV markers was performed at the population level aiming to identify CNVs significantly associated to specific populations and comparing the association statistics with those obtained from golden standard datasets.

The CNV algorithms used are described below:

- PennCNV [22] is one of the most frequently used methods for analyzing CNVs using Illumina microarrays. This software implements a CNV estimation method based on Hidden-Markov-Models (HMM), in which copy number calls are performed sample by sample by analyzing the sample LRR (i.e. absolute intensity) and BAF values at each marker. Default settings were used in the analysis of the available HapMap samples generating the PFB file (i.e. population frequency of B allele) from their genotyping data.
- QuantiSNP is also one of the well-known methods for CNV analysis over Illumina microarrays. It is based on an Objective Bayes Hidden-Markov Model that is used to set certain hyperparameters in the HMM priors (for details see Colella et al. [20]). Default settings were used in this analysis with the provided Infinum HD parameter files and the local GC content files.
- CNstream [24] was also evaluated in order to demonstrate how our new method overperforms the previous one due to the major critical modifications introduced.

Association statistics obtained by each algorithm were compared with those obtained from three recently published reference studies:

- The first dataset was obtained from a study published by McCarroll et al. [15]. In this study a hybrid genotyping array was designed to simultaneously measure SNPs and CNVs. Almost half (N = 1,320) of the targeted CNV regions were observed in multiple unrelated individuals and were defined as CNPs. From this set of CNPs we selected the autosomal CNPs (N = 1,292) over the 270 HapMap samples as the first golden standard dataset.
- We used the data published by Conrad et al. [10] as the second golden standard dataset. In this study, an Agilent CGH based array was used to generate a map of CNVs greater than 443 base pairs. For 4,978 of these CNVs reference genotypes from 450 HapMap samples are available to download. We used the corresponding sample calls for all the 4,899 autosomal CNVs.
- The last dataset used for CNV performance evaluation was obtained from the results published by Campbell et al. [9]. In this study a custom Agilent CGH microarray targeting regions of known CNPs was designed and evaluated over HapMap samples of diverse ethnic backgrounds. For this analysis we used the published discrete CNV calls of polymorphic loci represented in the reference genome assembly (N = 874) for the 487 HapMap samples included in the analysis.

In order to provide a measure of genome-wide association power, pairwise population-association tests (CEU:YRI, CEU:CHBJPT and YRI:CHBJPT) were performed using the calls from the three golden standard datasets. Loci that either were not covered by the microarray platform or did not obtain significant associations (*P*-value<0.05) in any population test (Table 2) were filtered out. Chi-square test *P*-values were then computed at each locus and compared to those obtained using the calls of the four methods across the markers covering the locus. For each algorithm, the marker obtaining the best result across the region was selected for comparison.

**Table 2:** CNV regions for each dataset and platform used to evaluate the power to detect genome-wide associations.

			HumanOmni1-Quad		Human1M-Duo	
Study	Technology	N_CNVRs_	N_CNVRs_ covered	N_ASSOCS_ (*P*-value<0.05)	N_CNVRs_ covered	N_ASSOCS_ (*P*-value<0.05)
McCarroll	Affymetrix	1292	1290	929	1288	927
Campbell	Agilent CGH	874	874	962	873	962
Conrad	Agilent CGH	4899	4899	3659	4899	3671

N_CNVR_ refers to the number of CNV loci selected from each study. Coverage with at least one marker within the CNV loci of both platforms is very similar although the marker density differs considerably. N_ASSOCS_ column refers to the total number of associated regions for the three population tests detected over the golden standard calls.

### Copy number variation and disease susceptibility

Using microarray data to accurately extract information from copy number variation can be particularly relevant when trying to identify all the type of variants that convey risk to disease susceptibility. Using two available catalogues of disease genomic associations we have used two different approaches to demonstrate the joint capacity of microarray platforms and the GStream method to identify new CNV disease associations. The two analysis explained here have been performed using the CNV calls inferred by GStream over the HapMap samples genotyped with the Illumina HumanOmni1-Quad platform.

#### Catalog of published genome-wide association studies

Since microarray genotyping platforms became available, a large number GWAS have allowed the discovery of important SNP-trait associations. However, some of these SNPs have limited or no known functional impact. In these cases, the possibility that they act as proxies of other types of variations (i.e. CNVs [36], [37]) with a deeper functional impact is more likely.

In order to identify putative causal CNVs we have analyzed the LD patterns between all the trait-associated SNPs reported by the catalog of published genome-wide association studies (http://www.genome.gov/gwastudies) [26] and the CNV microarray markers detected over the HumanOmni1-Quad platform. Trait-associated SNP genotypes were extracted from the 1KGP data reported previously and CNV genotypes were called with GStream. All the HumanOmni1-Quad markers that presented a non-diploid frequency greater than 1% (CNV markers) were included in the analysis (NCNV = 90,892) together with the 7,571 trait-associated SNPs.

The conditions used for selecting the candidate SNP-CNV pairs where the CNV could provide new functional information on the reported association are described below:

- Distance between the SNP and the CNV markers not greater than 50 kb.
- Correlation coefficient r^2^ greater than 0.7 in any of the three analyzed HapMap populations (CEU, YRI and CHB+JPT).
- Distance between the CNV marker and the nearest gene not greater than 100 kb or CNV marker spanning binding transcription factor regions as defined by the Transcription Factor ChIP-seq track on the UCSC browser [38].

From the 7,571 trait-associated SNPs, 382 were paired with one or more CNV markers fulfilling these conditions. A final set of 333 SNP-CNV pairs was obtained after filtering out repeated associations of SNPs with the same trait by different GWAS studies.

#### CNV overlapping analysis with disease-related genes

In this second approach, we examined the CNV variants called by GStream spanning genes known to be involved in disease based on the OMIM database (http://www.omim.org) [27]. In order to characterize CNVs with a high probability of conveying functional effects on the disease-related OMIM genes we set multiple strict selection criteria:

- From the initial set of CNV markers (NCNV = 90,892) only those located less than 15 kb away from an OMIM gene and with at least two more CNV markers covering this gene were selected (NCNV = 5,836).
- We defined CNV loci as sets of three or more nearer CNV markers (i.e. distance between them not greater than 5 kb) in high LD (r^2^>0.7) spanning the same OMIM gene. After applying this filter we obtained a final set of 212 CNV loci spanning OMIM genes.
- Finally, when more than one CNV locus spanned the same gene, only the one showing the greatest r^2^ measurements between its CNV markers was kept for further analysis.

The final set of candidates consisted of 149 CNV loci spanning disease-related OMIM genes.

### Software availability

An executable version of GStream along with the documentation and example data files can be freely downloaded from our website http://www.urr.cat/GStream. This web site also provides regularly updated results of new CNV associations within known human risk loci identified with this method.

## Results

### Performance assessment of SNP genotyping

For each available Illumina platform, the golden standard genotype calls were compared with the calls generated by GStream, GenoSNP, GenCall and M3 software tools. The global accuracy results over autosomal SNPs (Table 3) show a moderate improvement for GStream with respect to GenoSNP and a substantial improvement with regards to GenCall and M3 methods. GenCall performed very well when “non-called” genotypes where discarded, but its global performance decreased due to its low call rate. M3 algorithm could only be evaluated over the Human610-Quad and the Human660W platforms due to code incompatibilities with the Human1M-Duo and the HumanOmni1-Quad platforms. Although the improvement of GStream SNP-genotyping method regarding its competitors may not appear very high, they can represent a significant improvement from an absolute point of view (i.e. the accuracy differences when using HumanOmni1-Quad would be equivalent to a gain of 2,300 completely genotyped SNPs). Chromosome X genotyping accuracy was also evaluated, obtaining a similar decrease in performance (∼0.5%) for all the algorithms and maintaining the accuracy differences between algorithms.

**Table 3:** Global accuracy results for SNP genotyping.

Method	call rate (%)	accuracy (%)	global accuracy (%)	CNstream differential (%)
Human610-Quad				
GStream	99.952	99.798	**99.750**	
GenoSNP	100.000	99.577	99.577	−0.173
GenCall	96.724	99.868	96.596	−3.154
M3	99.584	99.585	99.171	−0.579
Human660W-Quad				
GStream	99.990	99.804	**99.794**	
GenoSNP	100.000	99.650	99.650	−0.144
GenCall	95.411	99.879	95.295	−4.499
M3	99.798	99.635	99.434	−0.360
Human1M-Duo				
GStream	99.970	99.768	**99.738**	
GenoSNP	100.000	99.561	99.561	−0.177
GenCall	98.024	99.825	97.853	−1.885
M3	NA	NA	NA	NA
HumanOmni1-Quad				
GStream	99.971	99.671	**99.643**	
GenoSNP	100.000	99.435	99.435	−0.208
GenCall	97.083	99.747	96.838	−2.805
M3	NA	NA	NA	NA

Call rate refers to the percentage of called genotypes while the accuracy is computed as the number of correct genotypes over the number of called genotypes. Global accuracy summarizes both measurements by computing the number of correct genotypes over the total number of genotypes available within the golden standard dataset.

**Figure 3:**
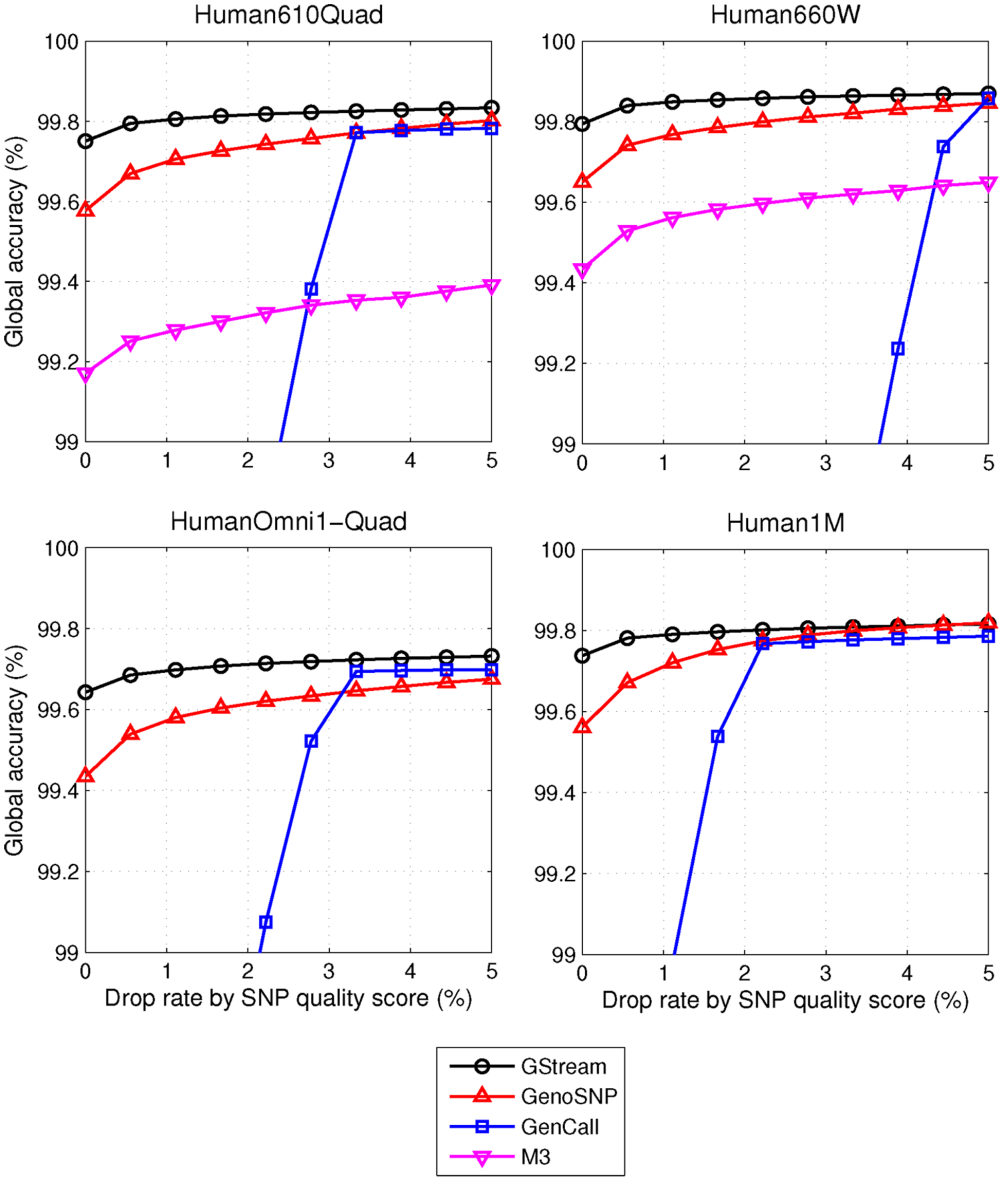
Evaluating SNP genotyping performance. Plots comparing SNP genotyping algorithms for each microarray platform are tested. The vertical axis represents the percentage of SNPs that are excluded from the accuracy calculation by the lowest quality score criteria. GStream performed better at all the drop rate levels in all the platforms. A high decrease in performance is observed for GenCall when drop rate values are lower than its uncall rate (i.e. ∼2% in Human610Quad).

The second performance test consisted of computing the global accuracies at different levels of drop rate, where drop rate refers to the percentage of markers which are removed from the accuracy computation based on low call confidence measures (as defined by Ritchie et al. [39]). When markers are discarded by low global marker quality score - a common filtering procedure in GWAS quality control steps - GStream reaches the best performance for all the evaluated drop rates (Figure 3). Furthermore, the difference in accuracy between low and high drop rates is much lower on GStream, which implies a robust genotyping performance, even for those markers with lower quality scores. The low accuracy values obtained using GenCall at low drop rates can be explained from its low call rate (i.e. only when the drop rate exceeds the uncall rate, GenCall performance is comparable to the other algorithms, otherwise uncalled SNPs are included in the performance evaluation). When discarding markers by low quality sample calls, the results show a similar pattern but with reduced accuracy differences between the algorithms (Figure S4A). These results place GStream as the best option for SNP genotyping, since its genotyping accuracy reaches its maximum at lower drop rates compared to the other algorithms.

We also examined the performance with respect to the minor allele frequency (MAF, Figure S4B). Two key conclusions result from this analysis: First, probes capturing rare SNPs (MAF<0.05) showed a slight accuracy reduction (∼0.5%) on all the platforms and algorithms tested and, second, GStream accuracy gain with respect to the other algorithms was practically independent of the SNP MAF.

Finally, we tested the effect of sample size on GStream accuracy (Figure S4C). The computed accuracy was compared to the accuracies obtained for the other algorithms when using all the Human610-Quad samples (N = 225). However, even for sample sizes as low as N = 20, the global accuracy of GStream is clearly higher than the accuracies obtained by the other algorithms, even if the highest sample size (N = 225) is used, demonstrating the superior sensitivity of GStream genotyping algorithm.

### Performance assessment of CNV genotyping

#### 1KGP Structural Variants

**Figure 4:**
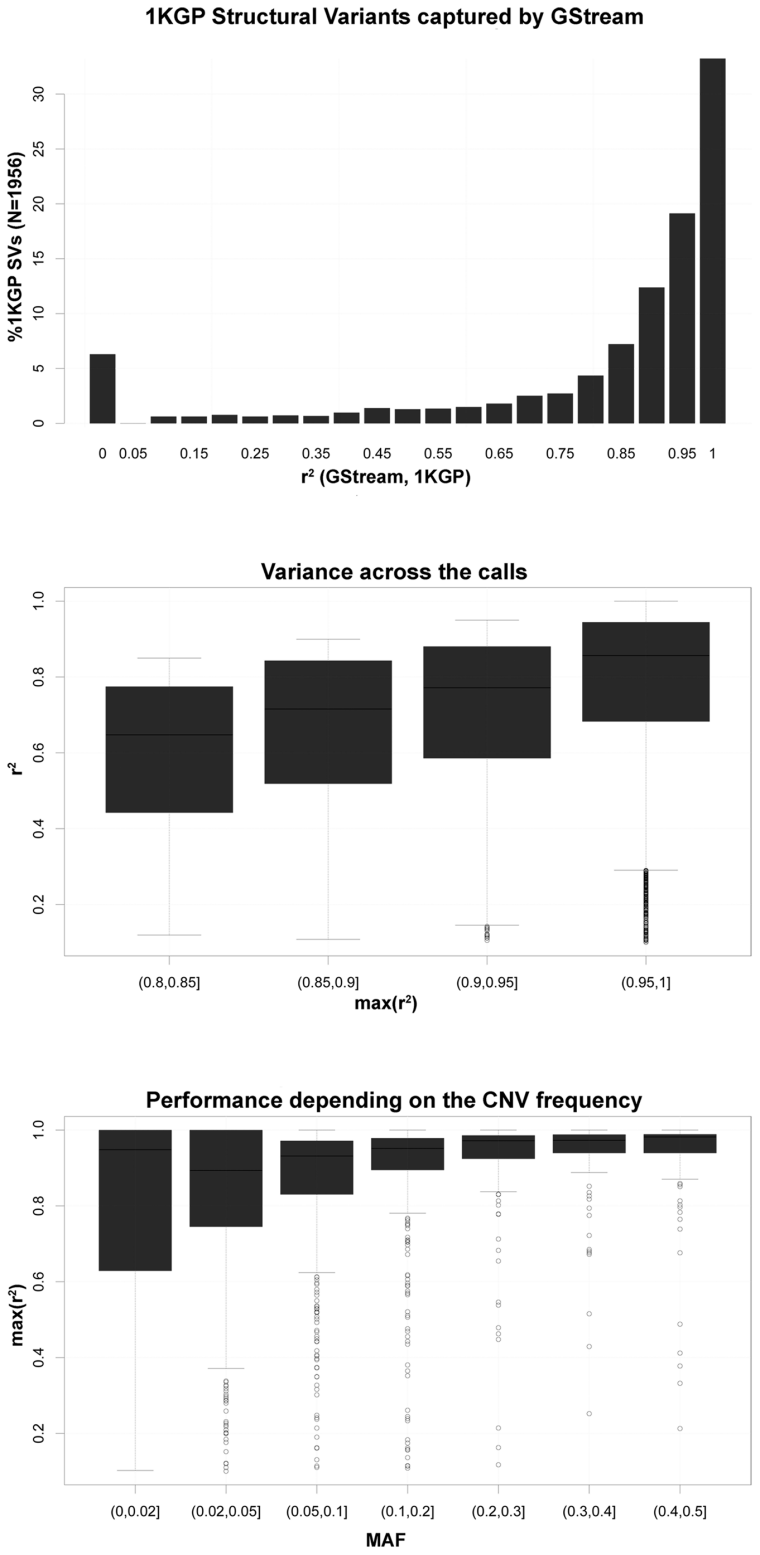
1KGP structural variants captured by GStream. (A) Percentage of 1KGP structural variants that are captured by GStream within different ranges of r^2^ between the 1KGP calls and the GStream calls over the best marker within the respective structural variant loci. (B) Distribution of the r^2^ values when more than one marker is found within the structural variant loci. Structural variants are stratified according to the best r^2^ obtained by all the markers covering the loci. (C) r^2^ distribution stratified by the frequency of the structural variation.

SV calls from 1KGP for 149 unrelated HapMap samples (i.e. N_CEU_ = 32, N_YRI_ = 37 and N_CHBJPT_ = 80) were compared with their respective GStream calls in order to measure the ability to detect this type of variation using GStream on microarray data. For each SV (N = 1,956), we computed the CNV genotyping accuracy by finding the maximum LD measurement between its golden standard calls and the GStream calls over the HumanOmni1-Quad markers covering the region. The results showed a high correlation between GStream and 1KGP calls: 75.7% of the SVs were captured by GStream with an r^2^>0.8, 18.3% with an r^2^<0.8 and only 6.0% were not detected by GStream (Figure 4A).

Once demonstrated the power of GStream to capture these variants, we examined the variance of the LD measurements across the markers spanning the same SV loci. This analysis was stratified by the maximum LD measurement of the SV as explained in the previous paragraph. From the results (Figure 4B) we can conclude that the calls inferred over probes spanning the same structural variant obtain consistent values with a slight variance due to the quality differences across markers.

Finally, we also observed that the calling performance slightly decreases with the frequency of the analyzed SVs due to an increment of the r^2^ interquartile ranges (Figure 4C). Nevertheless, lower quartiles exceeded r^2^ = 0.7 within almost all the frequency ranges tested.

We conclude this section by stressing the power of GStream to detect structural variation identified with more advanced technologies (i.e. NGS), obtaining CNV calls with an r^2^>0.8 over 75.7% of the 1KGP variants and calls with an r^2^>0.9 over 62.3% of the variants.

#### Genome-wide CNV association study

In order to evaluate the power to detect CNV associations we have performed a pairwise association study between three HapMap populations using golden standard data from three reference studies [9], [10], [15]. The association statistics obtained by the golden standard calls were compared with those obtained by GStream, CNstream1, PennCNV and QuantiSNP across two microarray platforms, HumanOmni1-Quad and Human1M-Duo. These two platforms were chosen since they represent the first and the second generation of the Infinium HD genotyping microarrays, which mainly differ by the inclusion of additional probes to obtain a better coverage of CNV loci. These differences (Figure S5) are clearly visible and the coverage analysis over the CNV regions defined by the three published studies showed how the marker density is doubled within these CNV regions between the first and the second platform generations (i.e. from 10 to 20 markers/region).

The main metric used to test CNV methods was the −log_10_ *P*-value ratio between the association values obtained by the calls of each method and those obtained from the golden standard calls. Under this metric, ratios near 1 represent a good performance for the method since the obtained association *P*-value is very similar to the golden standard. A performance summary statistic was computed as the percentage of −log_10_ *P*-value ratios giving values between 0.9 and 1.1 over all the associated CNV loci.

Firstly, the obtained results showed a high performance decrease in the detection of CNV associations when comparing HumanOmni1-Quad results with Human1M-Duo (Table 4). This loss was common to all the methods tested but PennCNV and QuantiSNP showed a higher percentage decrease (∼70%) than GStream and CNstream (∼40%). When comparing GStream to the other state-of-the-art methods, results showed a major performance gain on all the golden standard sets and on both microarray platforms. Results for each platform are described below.

**Table 4:** Power to detect CNP associations.

	HumanOmni1-Quad				Human1M-Duo				Platform diff.
	McCarroll^A^	Campbell^B^	Conrad^B^	Average	McCarroll^A^	Campbell^B^	Conrad^B^	Average	
GStream	56.40	46.80	63.87	55.69	45.09	27.65	24.38	32.37	23.32
CNstream	31.11	30.67	30.06	30.61	29.13	17.57	13.89	20.20	10.42
PennCNV	34.66	29.94	29.57	31.39	13.70	9.36	3.73	8.93	22.46
QuantiSNP	33.80	31.50	30.86	32.05	16.50	10.08	4.37	10.32	21.74

A CNV dataset provided by custom genotyping microarray-based studies.

B CNV datasets provided by CGH-based studies.

Percentage of −log_10_ *P* ratios higher than 0.9 and lower than 1.1 over the CNV population-associated regions computed for each study. Platform difference refers to the percentage differences between HumanOmni1-Quad and Human1M-Duo platforms.

**Figure 5:**
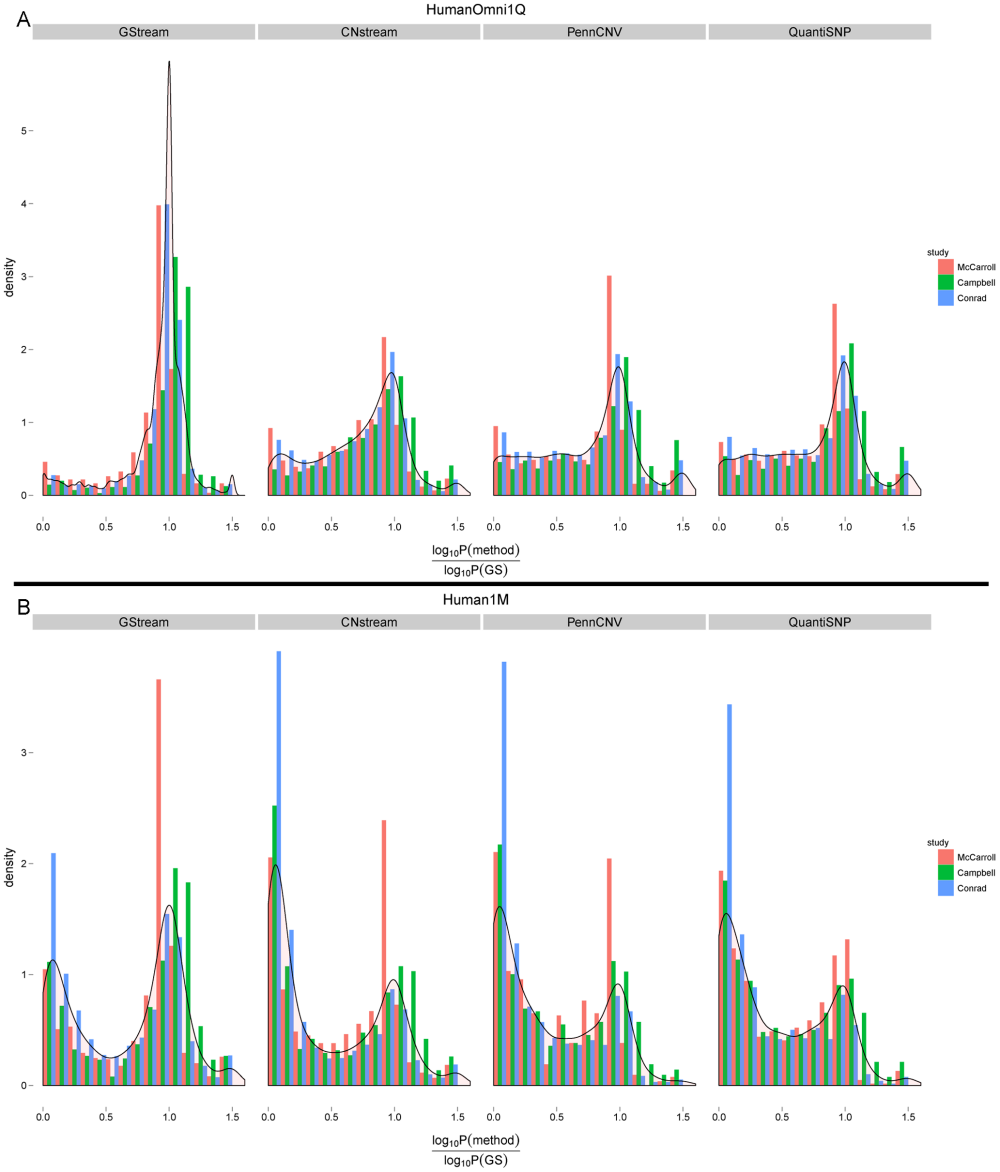
Evaluation of the power to capture genome-wide CNP association. Plots comparing Chi-square test *P*-values obtained with the golden standard calls (i.e. McCarroll, Campbell and Conrad datasets) with those obtained with the four tested methods using HumanOmni1-Quad (A) and Human1M-Duo (B) platforms. Comparison is performed by observing the distribution of the *P*-value association ratios (i.e. tested method versus golden standard). A high performance difference was obtained between the two platforms tested (i.e. due to their high difference in coverage density) and between GStream and the rest of algorithms tested.

HumanOmni1-Quad results show that GStream is able to precisely capture an average of 23.6% more associated loci than the other methods (Table 4) and that the number of false negatives (Figure S6) decreased considerably. Examining the association ratio distributions (Figure 5A), we also observed how GStream outperforms CNstream and, to a greater extent, PennCNV and QuantiSNP: while GStream ratio distribution resembles a unimodal distribution with a high kurtosis and centred around 0.95 (i.e. precisely captured loci), the rest of the distributions from CNstream, PennCNV and QuantiSNP showed a lower kurtosis with more ratio values distributed between 0 and 0.75 (i.e. loci not or poorly captured). To conclude, HumanOmi1-Quad comparison, we examined the association ratio distributions stratified by the *P*-value obtained by the golden standard calls (Figure S7). GStream obtains very good results within all the *P*-value ranges, with medians around 1 and decreasing interquartile ranges (from 0.2 to 0.02) as the *P*-value ranges decreased (i.e. greater evidence of association). Interquartile ranges obtained by the other methods were at least twice the obtained by GStream and increased as the *P*-value ranges decreased, losing performance when comparing higher associated loci. Poorer ratio medians were also obtained when using the other methods.

When comparing Human1M-Duo results, the performance differences between methods are similar to the previous comparison but with an absolute decrease in performance for all the methods due to previously referred differences on the platform design. Despite this global performance loss, GStream was able to precisely capture three times as many associations as PennCNV and QuantiSNP (Figure 5B). The number of associations not detected was also increased by a factor of 3 with respect to HumanOmni1-Quad results (Figure S6). When analyzing −log_10_ *P*-value ratio distributions by their respective golden standard association *P*-values, ratio medians were only maintained near one when using GStream (Figure S8). Instead CNstream, PennCNV and QuantiSNP showed a significant loss with ratio medians below 0.5.

### Copy number variation and disease susceptibility

Here we describe CNV associations that have been found by mining two available catalogues of disease genomic associations in order to demonstrate the power of GStream to identify new and functionally relevant CNV disease associations.

#### Catalog of published genome-wide association studies

A set of 333 SNP-CNV pairs have been identified when searching for CNV markers in high LD with trait-associated SNPs reported in the GWAS catalog (Table S1). From this set of paired associations, 94 spanned the HLA region, reflecting the known genomic complexity of this region. On the other hand, previously reported disease-associated CNVs were detected using this approach (Table 5 and Figure S9) like, for example, well-known deletions spanning *IRGM*, *LCE3* and *ARMS2* loci, which have been respectively associated to Crohn's disease [37], psoriasis [36] and age-related macular degeneration [40]. A 45-kb deletion near *NEGR1* gene and a 50-kb deletion upstream of *GPRC5B* gene previously associated to obesity and body mass index [41] were also identified.

**Table 5:** CNV loci highly correlated with trait-associated SNPs.

CHR	SNP	SNPbp	CNVbp	N	r2CEU	r2YRI	r2CHBJPT	pval	genes	GWAS_trait	PMID	Reported
1	rs2240335	17674537	17677196	12	0.82	0.65	0.95	2.E-08	PADI4	Rheumatoid arthritis	21505073	No
2	rs2867125	622827	623693	8	1.00	1.00	0.94	3.E-49	TMEM18	Body mass index	20935630	No
2	rs552976	169791438	169776139	5	0.94	0.26	0.32	8.E-18	ABCB11	Glycated hemoglobin levels	20858683	No
4	rs6815464	1309901	1290281	3	1.00	0.38	0.91	2.E-20	MAEA	Type 2 diabetes	22158537	No
6	rs6904029	29943067	29942384	5	1	1	0.89	1E-21	HCG9	Vitiligo	20410501	No
6	rs3077	33033022	33030885	3	0.61	0.94	1.00	2.E-61	HLA-DPA1	Hepatitis B	21750111	No
6	rs9296736	53924697	53930407	11	1.00	1.00	0.96	3.E-09	MLIP	Liver enzyme levels	22001757	No
7	rs2075671	100345106	100329189	12	0.87	0.00	0.91	1.E-09	ZAN	Red blood cell count	19862010	No
19	rs7247513	12691185	12694963	13	1.00	1.00	0.81	2.E-06	ZNF490	Bipolar disorder	21254220	No
1	rs2568958	72765116	72769429	14	1.00	1.00	1.00	1.E-11	NEGR1	Body mass index	19079260	Yes
1	rs2568958	72765116	72769429	14	1.00	1.00	1.00	2.E-08	NEGR1	Weight	19079260	Yes
1	rs2815752	72812440	72769429	14	1.00	1.00	1.00	2.E-22	NEGR1	Body mass index	20935630	Yes
1	rs4085613	152550018	152557073	46	1.00	1.00	0.97	7.E-30	LCE3E;LCE3D;LCE3C	Psoriasis	19169255	Yes
1	rs4112788	152551276	152557073	46	1.00	0.46	0.97	3.E-10	LCE3E;LCE3D;LCE3C	Psoriasis	20953190	Yes
5	rs13361189	150223387	150178347	27	1.00	1.00	1.00	2.E-10	IRGM	Crohns disease	17554261	Yes
5	rs1000113	150240076	150181492	13	1.00	0.39	0.91	3.E-07	IRGM	Crohns disease	17554300	Yes
5	rs11747270	150258867	150203780	12	1.00	0.71	1.00	3.E-16	IRGM	Crohns disease	18587394	Yes
5	rs7714584	150270420	150212972	8	1.00	0.69	1.00	8.E-19	IRGM	Crohns disease	21102463	Yes
10	rs10490924	124214448	124217287	10	1.00	0.94	0.90	0.E+00	ARMS2	Age-related macular degeneration	21665990	Yes
10	rs3793917	124219275	124216893	10	1.00	1.00	0.95	4.E-60	ARMS2	Age-related macular degeneration	20385819	Yes
10	rs11200638	124220544	124216893	10	1.00	1.00	0.95	8.E-12	ARMS2	Age-related macular degeneration	17053108	Yes
16	rs12444979	19933600	19949684	8	1.00	1.00	0.00	3.E-21	GPRC5B	Body mass index	20935630	Yes

This table shows significant SNP-CNV pairs found in high LD. N stands for the number of CNV microarray markers correlated with the SNP genotypes, and r2CEU, r2YRI and r2CHBJPT stand for the linkage disequilibrium measures between the SNP and the CNV. Reported GWAS *P*-value is also shown together with a field indicating if the CNV association has been previously reported.

A thorough study of the CNVs in high LD with trait-associated SNPs revealed several interesting loci. Some of these loci are described below (Table 5 and Figure S10):

- A synonymous exonic SNP rs2240335 (*PADI4* gene) has been associated to Rheumatoid Arthritis (RA) in a previous GWAS (*P*-value = 2E-8) using a Japanese cohort (1247 RA cases and 1486 controls) [42]. GStream found 12 CNV markers 2 kb away and spanning *PADI4* intron (length  = 800 bp) in high LD with rs2240335 both on CEU (r^2^ = 0.82) and CHB+JPT (r^2^ = 0.95) HapMap populations.
- An intergenic SNP rs2867125 (50 kb downstream *TMEM18* gene) has been associated to body mass index in previous GWAS (*P*-value = 3E-49) [41]. Two CNV loci near this SNP have been identified by GStream in high LD (r^2^ = 1 both on CEU and CHB+JPT populations) with this SNP.
- Levels of glycated hemoglobin have been associated to the *ABCB11* intronic SNP rs552976 (*P*-value = 8E-18) [43]. A deletion variant 3 kb upstream of this gene showed a high correlation (r2 = 0.94 CEU) with the associated SNP genotypes.
- Intronic SNP rs6815464 on *MAEA* gene has been associated (*P*-value = 2E-20) with type 2 diabetes [44]. This SNP has been found to be highly correlated with another intronic deletion spanning ∼2 kb.
- 5′UTR SNP rs6904029 (*HCG9* gene) has been previously associated with Vitiligo (*P*-value = 1E-21) [45]. Here we report a CNV locus spanning the 5′ region of the *HCG9* gene found in high LD with this SNP.
- Intronic SNP rs3077 (*HLA*-*DPA1* gene) has been recently associated to chronic Hepatitis B (*P*-value = 2E-61) on a Japanese cohort [46]. A deletion potentially spanning three *HLA*-*DPA1* exons has been identified to be highly correlated with this SNP both on the CHB+JPT and the YRI populations.
- A deletion highly correlated with intronic SNP rs9296736 (*MLIP* gene) has been identified spanning 5 kb *MLIP* intron. This SNP has been associated with liver enzyme levels (*P*-value = 3E-9) [47].
- Intronic SNP rs2075671 (*ZAN* gene) has been associated to red blood cell count (*P*-value = 1E-9) on a GWAS exploring erythrocyte phenotypes [48]. A deletion locus spanning multiple exons of the same gene has been found with a high correlation with the associated SNP genotypes both on the CEU (r^2^ = 0.87) and the CHB+JPT (r^2^ = 0.91) populations.
- Finally, the 3′UTR SNP rs7247513 (*ZNF490* gene), modestly associated with bipolar disorder (*P*-value = 2E-6) [49], was also found in high LD with a 2 kb deletion covering *ZNF490* intron.

A complete list of all the 333 associations can be consulted on Table S1 and, as previously mentioned, will be regularly updated in our website.

#### CNV overlap with disease-related genes

In this second approach we examined the CNV variants called by GStream spanning genes known to be involved in disease. A set of 149 CNV consistent loci spanning OMIM genes were obtained (Table S2) with a mean length of 6 kb and a mean number of 7 probes per CNV loci.

In this analysis, well-known associated deletions were found. For example, a common CFH haplotype with deletion of *CFHR1* and *CFHR3* genes associated with lower risk of age-related macular degeneration [50] was identified using the GStream CNV calls. A CNV spanning *CCL3L*-*CCL4L* genes has been extensively associated with various human immunodeficiency virus-related outcomes [51] and was also identified in this analysis. *SMN*, *GHR* and *PKHD1* gene deletions respectively associated to spinal muscular atrophy [52], responsiveness to growth hormone [53] and polycystic kidney disease [54] were also detected using GStream (Figure S11). Besides these deletions that have already been associated to disease risk, GStream has also allowed us to identify new exon spanning deletions within disease-associated genes (Table S2). Some examples of these findings are the deletions covering *SLC2A9*, *DAZL* and *MBL2* gene exons.

In almost all these identified CNV loci, GStream calls across the probes within the loci showed a high concordance demonstrating a high performance for a high variety of CNV cluster intensity patterns (Figure S12).

## Discussion

In this study we present GStream, an integrated tool for SNP and CNV genotyping addressed to Illumina microarray data. This new tool has been carefully designed to obtain a high performance in genotyping accuracy when analyzing GWAS data from Illumina BeadChip arrays. The performance of GStream has been assessed using reference data, extracted from the latest releases of the 1KGP and the HapMap projects, as well as from reference studies on CNV characterization. First, we show that GStream has superior SNP and CNV genotyping performance than current state-of-the-art methods. Second, we demonstrate its power to detect new structural variation recently identified with Next-Generation Sequencing technology. Finally, we also demonstrate the utility of GStream in the identification of CNVs within trait risk loci as well as known disease-associated genes. The newly identified CNV associations could help to advance in the understanding of the genetic basis of several human traits.

In a current scenario where genotyping microarrays are decreasing in cost and widening their spectrum of analyzed SNPs to more rare variations [55], the need of developing methods which increase SNP genotyping accuracy is even more fundamental. To this end, GStream provides a way of facilitating this success by obtaining the best performance results compared to the available state-of-the-art methods (i.e. GenCall [34] and GenoSNP [33]). This increased performance can be particularly meaningful in the case of identifying rare disease-associated SNPs, traditionally more exposed to genotyping errors and to the subsequent statistical bias [56], [57]. On the other hand, the accuracy of current SNP imputation methods [58], which expand the number of analyzed SNPs and also help to integrate the results obtained with different microarray platforms (GWAS meta-analysis), also depends on the quality of the originally genotyped SNPs. Therefore, prioritizing accurate SNP genotyping methods is a key success factor in order to obtain reliable imputation results [59].

Besides the importance of SNPs as a source of genetic variation, CNVs have also emerged as important variations for common trait risk [60] as evidenced by recent GWASs [61], [62], [63], [64], [65]. In the present study we tested our algorithm power to detect CNV loci that have been recently identified with the Next-Generation Sequencing technology. This NGS CNV data provided by the 1KGP includes not only previously known CNVs (i.e. detected with CGH arrays), but also new CNV loci that have not been previously detected. Since part of these loci are covered by microarray probes, their detection with microarray-based technologies is therefore possible. On the other hand, previous state-of-the-art methods for copy number genotyping [20], [22] present a lack of performance when CNVs span a few number of probes or when intensity distributions corresponding to the different copy number states partially overlap. The multi-component intensity distribution models implemented in GStream will allow researchers to deeply scan the genome for additional CNVs, widening their range to shorter, population-specific and/or previously uncharacterized CNVs.

In this study we also present a two-level comparison of the power of GStream to detect CNV associations in a population-based study. First, we have performed a comparison between the different algorithms tested and, second, we have performed a comparison between the two genotyping microarray generations represented by the Human1M-Duo and HumanOmni1-Quad platforms, this last one including a specific set of markers covering known CNV loci [55]. On the one hand, we confirmed the improvement introduced by the new generation microarrays as a consequence not only of their major density coverage within predefined CNV regions, but also of their improved signal quality. The number of correctly genotyped CNV regions (i.e. characterized in previous reference studies) increased in ∼20% when using HumanOmni1-Quad rather than Human1M-Duo, regardless of the CNV genotyping method being used. On the other hand, when comparing the results obtained by each algorithm tested, GStream showed a higher performance within all the scenarios. Its power increase for detecting and correctly genotyping CNVs (i.e. defined by three different reference studies based either on CGH or custom genotyping arrays) ranges from 50% to 100% compared with the best scoring of the other state-of-the-art methods. Therefore, we present GStream as an integrated SNP-CNV genotyping tool that shows a remarkable leap in performance with respect to previous methods.

One of the most important tasks when analyzing GWAS results is to link the associated variant to a functional effect that can explain the disease risk association. Identifying this link is not always easy since the identified variation can act as a proxy to the underlying causal mutation and may not be covered by the microarray platform. Actually, microarray probe design is based on the study of the linkage disequilibrium patterns and the resulting haplotypes that are inherited in blocks [66]. In this regard, we have identified several CNVs in high LD with SNPs that have been previously associated to disease susceptibility. A clear example of these linked CNVs are the *IRGM1* [37] and the *LCE3B*/*LCE3C* [36] deletions which have been associated to Crohn's Disease and Psoriasis, respectively. Furthermore, these two deletions have been demonstrated to affect the expression of the deleted genes. In addition to these previously known associations, we have identified additional CNVs previously not associated with the disease that could also have functional impact. For example, several CNVs spanning hundreds of bases of gene introns have been found to highly correlate with disease-associated SNPs. These CNVs could provide a functional link to the associated risk modifying, for example, RNA splicing [10], [67].

Furthermore, as CNV are known to span from hundreds of bases to multiple kilobases, it is interesting to analyze not only those that correlate previously associated SNPs, but also those that overlap coding sequences of genes that have been previously associated to human disease (i.e. OMIM genes). The results from this analysis include several known CNV associated loci, as those spanning *CFHR1*/*CFHR3* [65], *CCL3L*/*CCL4L* [51], *SMN* [52], *GHR* [53] and *PKHD1* [54] genes. More importantly, new interesting CNV loci also appeared, as those spanning *SLC2A9*, *DAZL* and *MBL2* disease-associated gene exons. The *SLC2A9* gene (OMIM 606142) deletion has been identified by GStream within eight microarray probes spanning two gene exons and two gene introns (chr4:9,929,128-9,966,793). Since mutations within this gene have been previously associated to uric acid concentration [68] and to Hypouricemia [69], the functional effects of this deletion should be further evaluated in relation to these traits. Indeed, GLUT9ΛN (resulting from alternative splicing of *SLC2A9* gene) is predominantly expressed in the kidney and expression association signals reported by Doring et al. [68] link this gene to the regulation of urate concentrations. The described exon deletion could probably imply a similar effect by modifying the resulting transcribed protein. On the other hand, *DAZL* (OMIM 601486) deletion was identified over 4 microarray probes spanning 5.8 kb (chr3:16,638,525-16,644,130). This deletion could affect multiple gene exons resulting in a drastic functional modification. Previous studies [70] have linked variants within this gene with susceptibility to spermatogenic failure and therefore, this deletion should be evaluated in the context of this human trait. GStream also found a relevant deletion spanning the last exon of the *MBL2* gene. *MBL2* mutations and the consequent Mannose Binding Lectin deficit have been previously associated with cystic fibrosis [71] and recovery from infections [72]. This deletion could drastically modify *MBL2* gene expression and subsequently involve a Mannose Binding Lectin deficit whose association has also been demonstrated.

In a time of rapidly evolving technologies and where Next-Generation Sequencing is becoming available for the study of common diseases, microarray-based technologies are still a commonly used strategy to identify the genetic basis of human traits. First, they allow the analysis of large sample collections at an affordable cost and, second, they have anincreasing global genome coverage, expanding their analysis scope to rarer variants. Therefore, accurate genotyping methods are basic to discover new associated loci that can be then further studied in more detail using Next-Generation Sequencing. The tool that we present in this study, GStream, provides an unprecedented accuracy when analyzing GWAS data from previous and recent Illumina microarray platforms. Furthermore, our software tool implementation allows large-scale GWAS projects to be analyzed in a very short time, providing both SNP and CNV in a single analysis. With these results, we encourage researchers conducting GWAS on these genotyping platforms to use GStream in order to leverage the power of their SNP and CNV loci association analyses.

## Supporting Information

Figure S1 **Example of raw intensity normalization.** The intensity distribution of candidate homozygote samples (i.e. AA) across its specific allele channel (i.e. channel A) is plotted together with its centroid scaling value as computed by Peiffer *et al.* [29]. GStream first weights this distribution and computes its maximum to scale the channel intensities by the corresponding intensity value. This example shows a typical CNV pattern where the error produced by the first approach is magnified.
(PDF)

Figure S2 **Example of how zero-threshold is computed.** (A) BAF and absolute intensities of an example marker where some homozygous deletion samples with low intensity values can be observed. (B) Absolute intensities are sorted and differences between consecutive sorted intensities normalized to one. The observed peak over these differences points to the intensity value that will be set as threshold.
(PDF)

Figure S3 **CNV labelling and scoring.** (A). Category disambiguation when the two-component model is selected. The leftmost graph shows a case where the higher intensity component (blue) is more frequent and it is assigned to the diploid state while the lower intensity component (red) is assigned to the deletion state. This assignment is due to the fact that high frequency amplifications are very uncommon and undetectable with this technology. The centre graph shows a case where the higher intensity component is less frequent and homozygous deletion samples have been found (magenta). In this case, the higher component (blue) is assigned to the diploid state and the lower component (red) to the deletion state fulfilling the expected Hardy-Weinberg equilibrium frequencies. Finally, the rightmost graph shows the last case where the higher intensity component is less frequent and no homozygous deletion samples have been found. In this case the higher component is assigned to the amplification state and the lower component to the diploid state. (B) Posterior probability of each copy number depending on the score assigned by GStream: From 0 to 0.5 samples can be categorized as homozygous deletion, from 0.5 to 1.5 as hemizygous deletion, from 1.5 to 2.5 as diploid and from 2.5 to 3 as amplification.
(PDF)

Figure S4 **Genotyping performance.** (A) Genotyping performance depending on the drop rate, where calls dropped from the accuracy analysis were selected according to the genotype call quality score. (B) Genotyping performance depending on the SNP minor allele frequency. (C) Genotyping accuracy of GStream at different sample sizes (i.e. N = 20, 40, 60, 80, 160 and 225) compared to the accuracies obtained by GenoSNP and M3 with the highest sample size (N = 225).
(PDF)

Figure S5 **Microarray coverage density.** Coverage density of each microarray platform over the CNV regions defined by each reference study. There are major differences between the first Infinum HD platforms (Human610-Quad and Human1M-Duo) and their succesors including specific CNV coverage (Human660W-Quad and HumanOmni1-Quad). Both Human610-Quad and Human1M-Duo have a mean number of ∼10 markers covering CNV regions, while Human660W was designed with a highest coverage (∼20 markers/region) for almost 50% of the regions. Finally, HumanOmni1-Quad increased the global coverage to ∼20 markers/region.
(PDF)

Figure S6 **Missed associations.** Percentage of associations (i.e. *P*-value<0.05 over the golden standard dataset) that were not captured by the different methods tested (i.e. *P*-value>0.05 over the tested method).
(PDF)

Figure S7 **HumanOmni1-Quad** ***P*** **-value distributions.** Distributions of the *P*-value association ratios depending on the golden standard dataset used for evaluation (i.e. represented by different colours) and on the *P*-value range obtained over the golden standard calls (i.e. horizontal axis).
(PDF)

Figure S8 **1M-Duo** ***P*** **-value distributions.** Distributions of the *P*-value association ratios depending on the golden standard dataset used for evaluation (i.e. represented by different colours) and on the *P*-value range obtained over the golden standard calls (i.e. horizontal axis).
(PDF)

Figure S9 **Previously reported CNV associations detected by LD analysis between GStream CNV genotypes and trait-associated SNPs.** (A) *LCE* gene cluster deletion associated with Psoriasis risk. (B) *NEGR1* deletion associated with body mass index. (C) *IRGM* deletion associated with Chrohn's disease. (D) *ARMS2* deletion associated with age-related macular degeneration. (E) *GPRC5B* upstream deletion associated with body mass index.
(PDF)

Figure S10 **Interesting CNV associations detected by LD analysis between GStream CNV genotypes and trait-associated SNPs.** (A) *PADI4* gene deletion associated with Rheumatoid Arthritis. (B) *TMEM18* downstream deletion associated with body mass index. (C) 3′-deletion of gene *ABCB11* associated with glycated hemoglobin levels. (D) *MAEA* gene intron deletion associated with type 2 diabetes. (E) *HCG9* deletion associated with Vitiligo. (F) *HLA*-*DPA1* deletion associated with Hepatitis B. (G) *MLIP* intron deletion associated with liver enzyme levels. (H) *ZAN* gene deletion associated with red blood cell count. (I) *ZNF490* intron deletion associated with bipolar disorder.
(PDF)

Figure S11 **GStream detected CNP loci spanning disease-related genes (OMIM) where CNVs have been previously associated with disease.** (A) CNP spanning *CFHR1* and *CFHR3* previously associated to age-related macular degeneration. (B) Deletion of *GHR* exon 3 that has been previously associated with increased responsiveness to growth hormone and Laron dwarfism. (C) Detected *SMN* gene deletion previously associated with spinal muscular atrophy. (D) *PKHD1* deletion associated with polycystic kidney. (E) *CCL3L1*/*CCL3L3* deletion previously associated with susceptibility to HIV/AIDS.
(PDF)

Figure S12 **GStream calls across consecutive markers spanning the same CNV loci.** These 6 microarray probes cover the same CNV loci but show very different CNV intensity patterns. GStream is completely adapted to these types of variations and its calling procedure is able to obtain very concordant calls when analyzing probes spanning the same CNV.
(PDF)

Table S1 **CNV markers in high LD with trait-associated SNPs reported in the GWAS catalog.**
(XLSX)

Table S2 **Set of 149 CNV consistent loci spanning OMIM genes.**
(XLSX)

Text S1 **GStream algorithm details.**
(PDF)
