# Supplementary figures and images for: GStream: Improving SNP and CNV Coverage on Genome-Wide Association Studies
Source: PLoS One. 2013 Jul 3;8(7):e68822. doi: 10.1371/journal.pone.0068822 (PMC3700900; doi:10.1371/journal.pone.0068822)

Intensity normalization

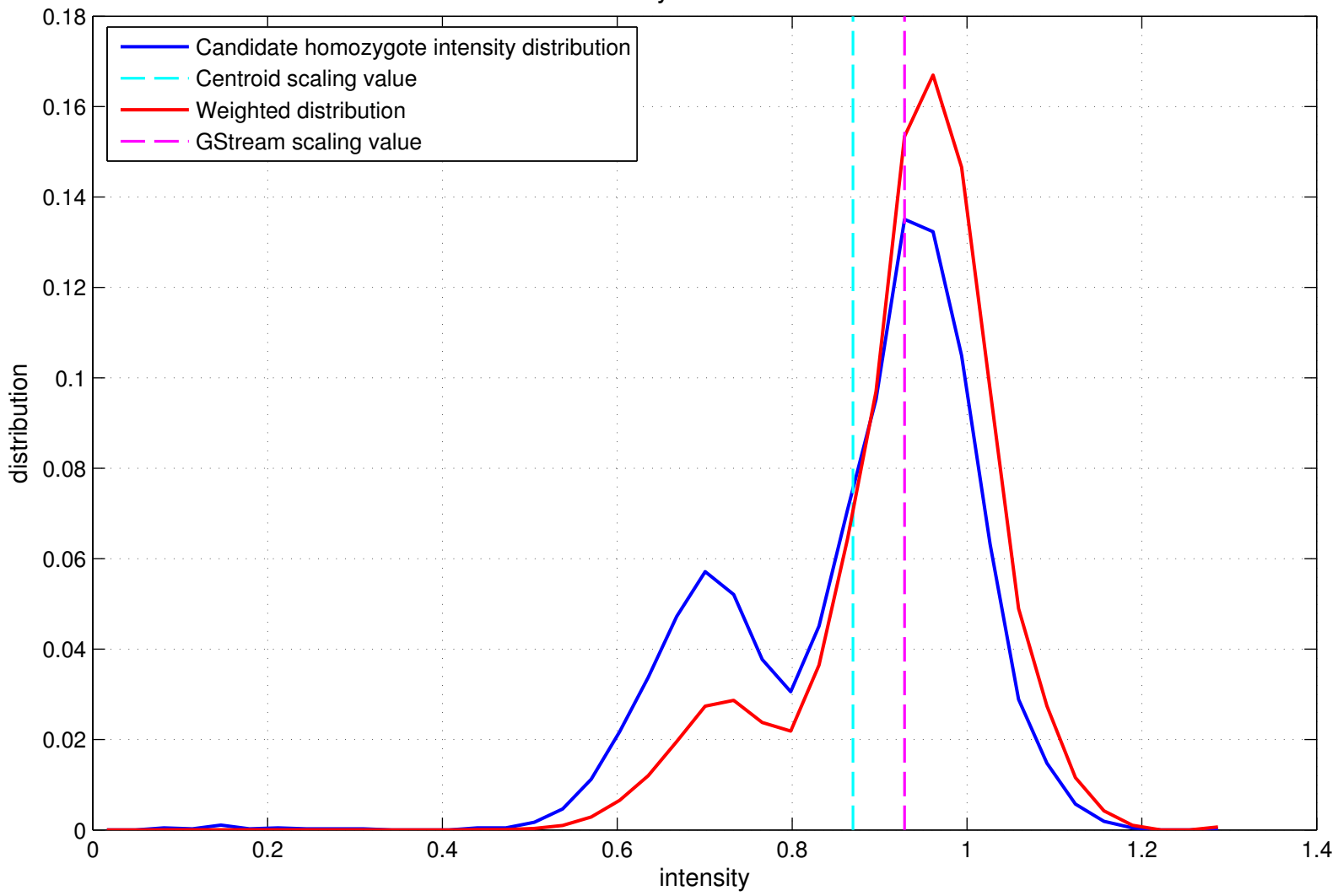

Supplement: Figure S1 — Example of raw intensity normalization. The intensity distribution of candidate homozygote samples (i.e. AA) across its specific allele channel (i.e. channel A) is plotted together with its centroid scaling value as computed by Peiffer et al. [29]. GStream first weights this distribution and computes its maximum to scale the channel intensities by the corresponding intensity value. This example shows a typical CNV pattern where the error produced by the first approach is magnified. (PDF) [file pone.0068822.s001.pdf]

INT vs BAF

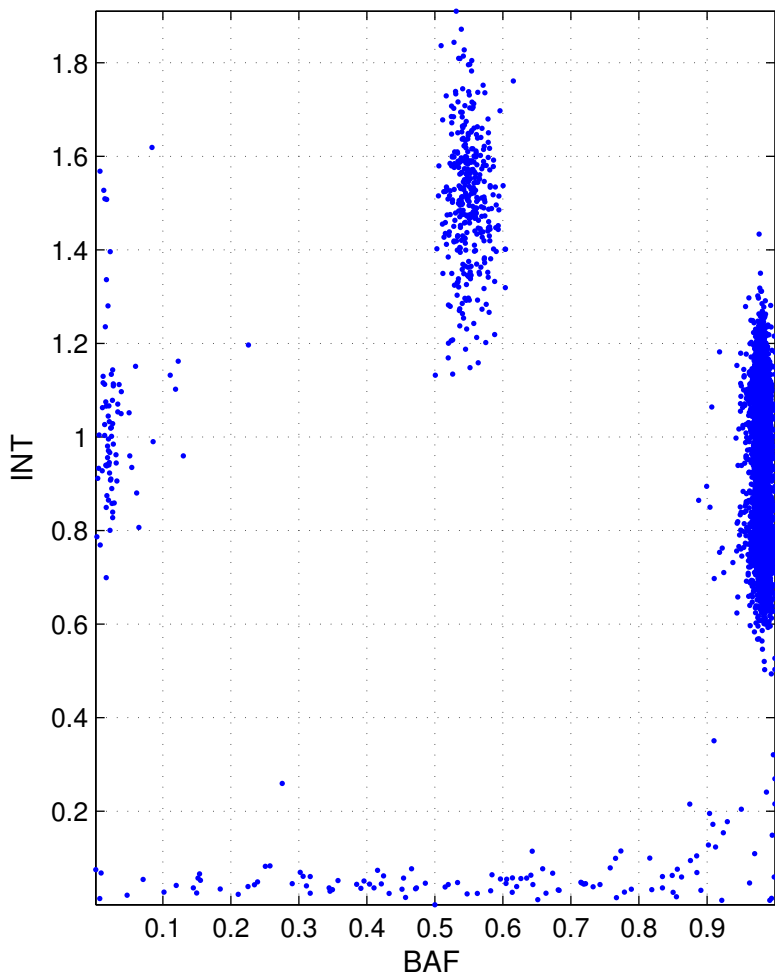

Sorted intensities

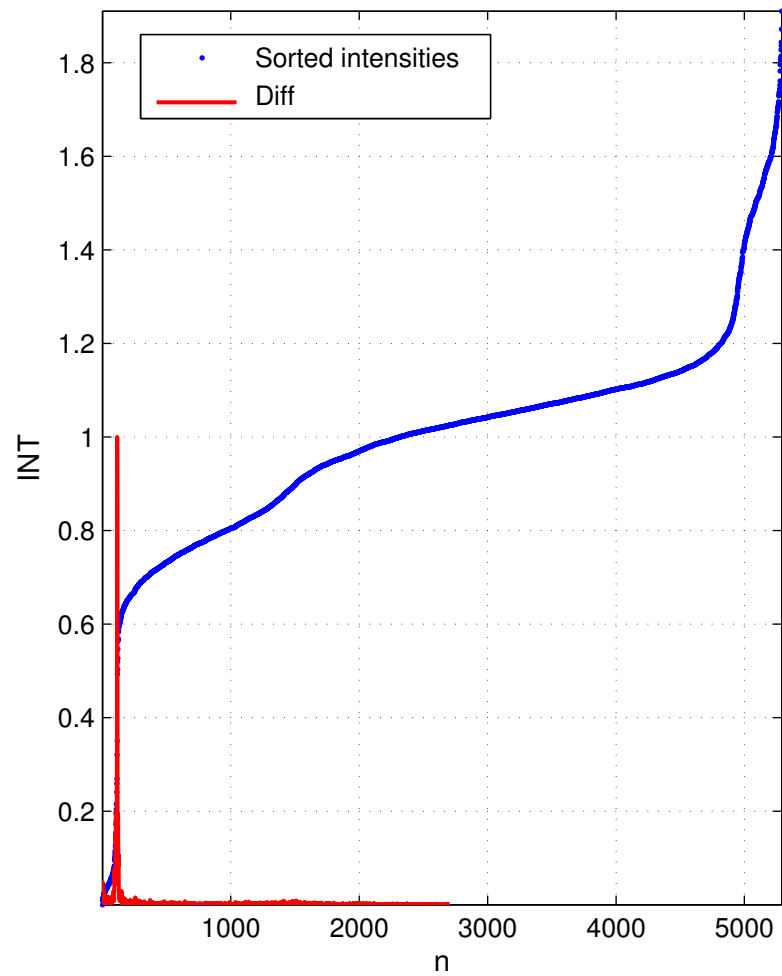

Supplement: Figure S2 — Example of how zero-threshold is computed. (A) BAF and absolute intensities of an example marker where some homozygous deletion samples with low intensity values can be observed. (B) Absolute intensities are sorted and differences between consecutive sorted intensities normalized to one. The observed peak over these differences points to the intensity value that will be set as threshold. (PDF) [file pone.0068822.s002.pdf]

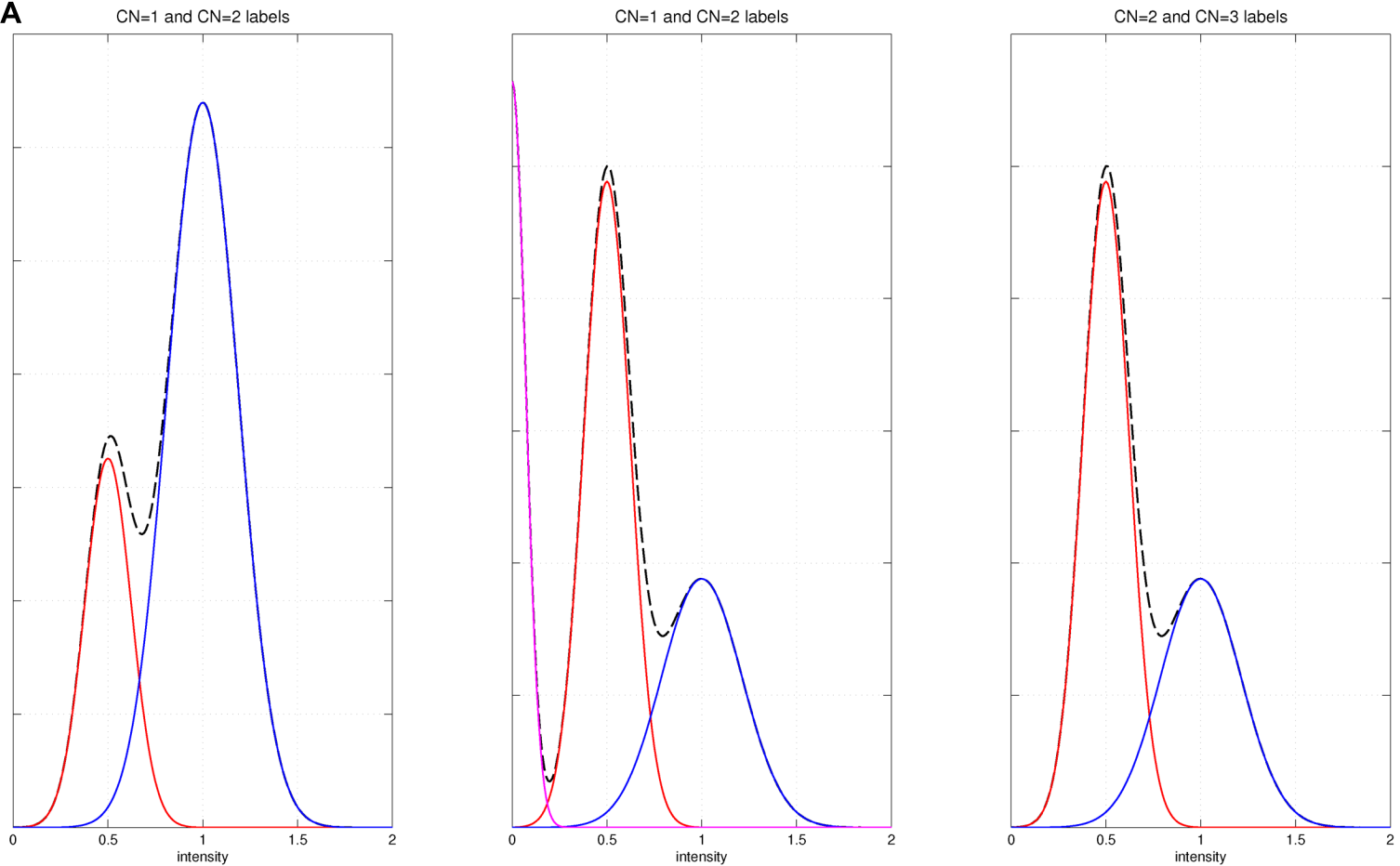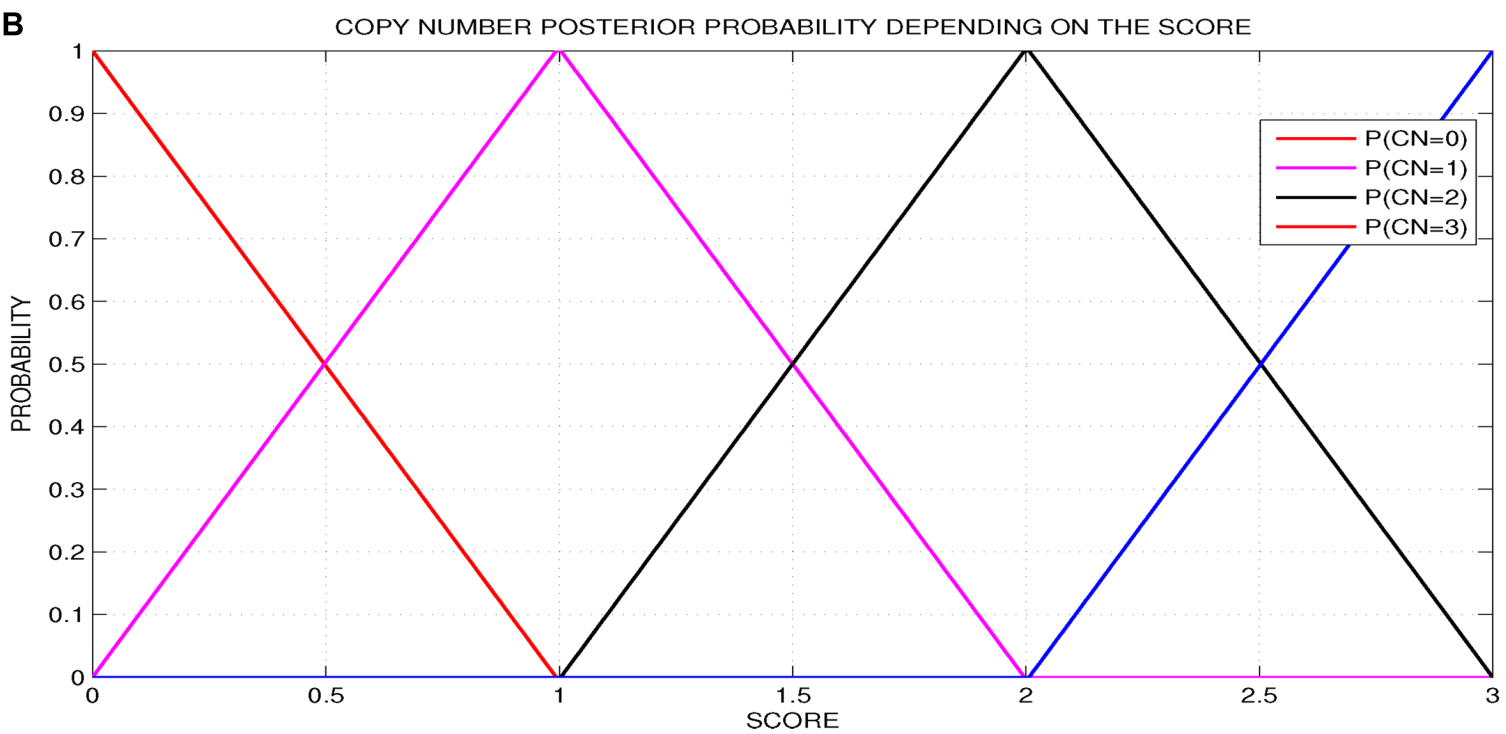

Supplement: Figure S3 — CNV labelling and scoring. (A). Category disambiguation when the two-component model is selected. The leftmost graph shows a case where the higher intensity component (blue) is more frequent and it is assigned to the diploid state while the lower intensity component (red) is assigned to the deletion state. This assignment is due to the fact that high frequency amplifications are very uncommon and undetectable with this technology. The centre graph shows a case where the higher intensity component is less frequent and homozygous deletion samples have been found (magenta). In this case, the higher component (blue) is assigned to the diploid state and the lower component (red) to the deletion state fulfilling the expected Hardy-Weinberg equilibrium frequencies. Finally, the rightmost graph shows the last case where the higher intensity component is less frequent and no homozygous deletion samples have been found. In this case the higher component is assigned to the amplification state and the lower component to the diploid state. (B) Posterior probability of each copy number depending on the score assigned by GStream: From 0 to 0.5 samples can be categorized as homozygous deletion, from 0.5 to 1.5 as hemizygous deletion, from 1.5 to 2.5 as diploid and from 2.5 to 3 as amplification. (PDF) [file pone.0068822.s003.pdf]

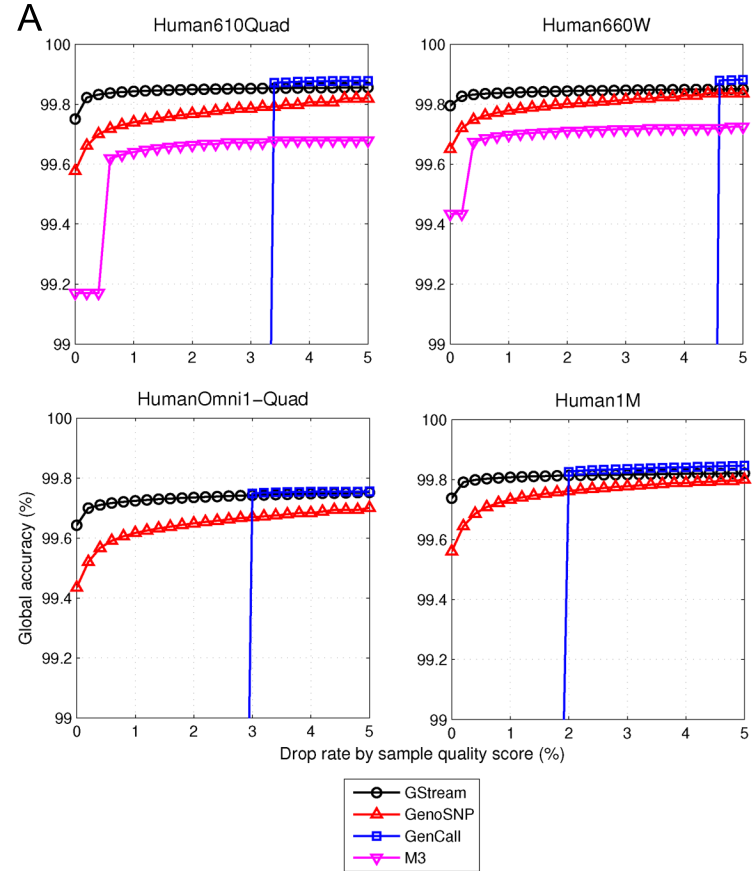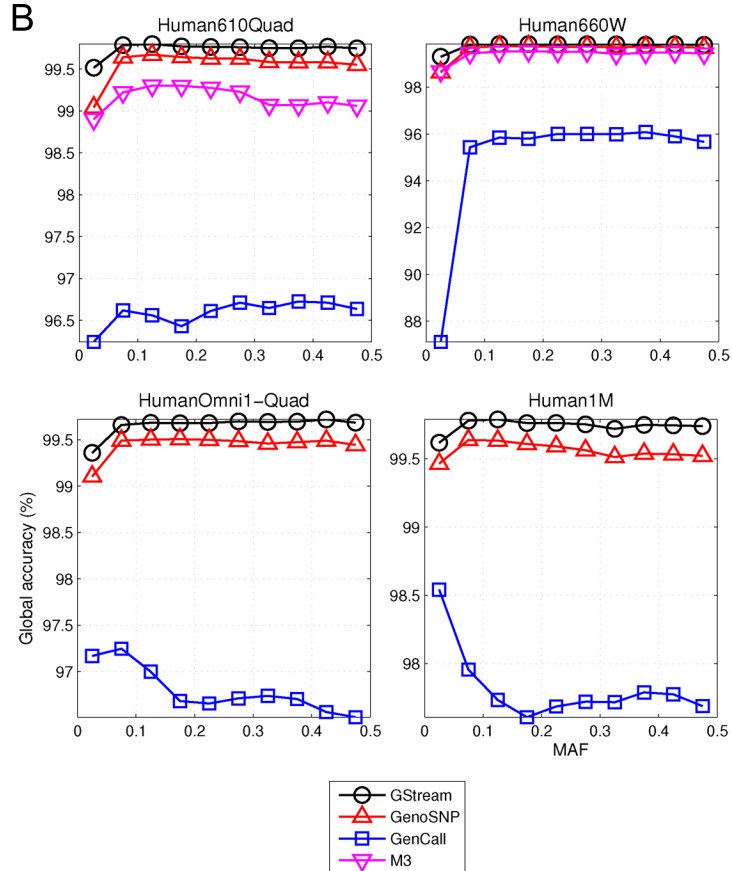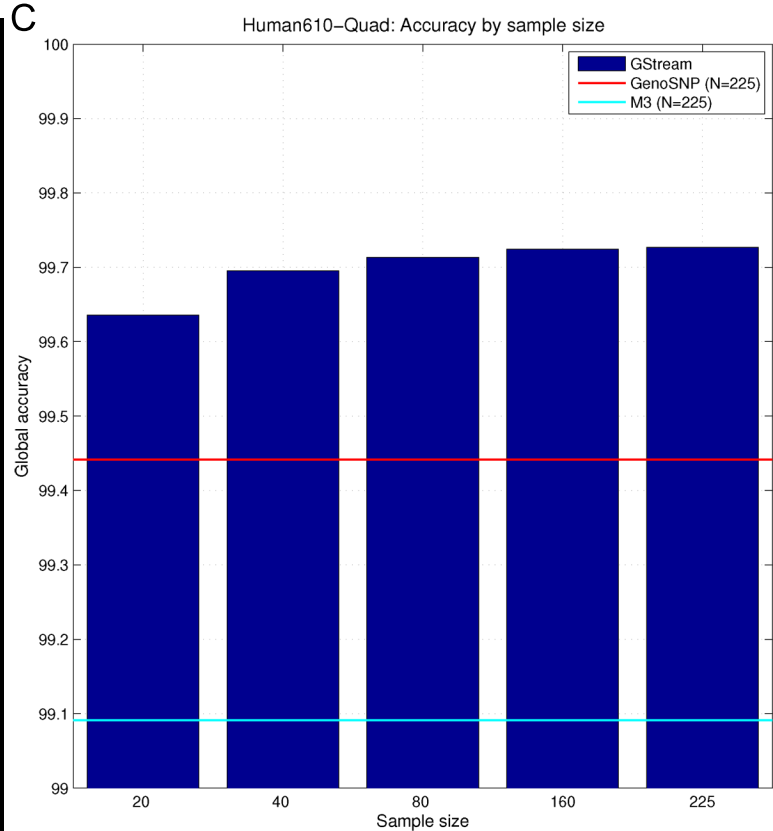

Supplement: Figure S4 — Genotyping performance. (A) Genotyping performance depending on the drop rate, where calls dropped from the accuracy analysis were selected according to the genotype call quality score. (B) Genotyping performance depending on the SNP minor allele frequency. (C) Genotyping accuracy of GStream at different sample sizes (i.e. N = 20, 40, 60, 80, 160 and 225) compared to the accuracies obtained by GenoSNP and M3 with the highest sample size (N = 225). (PDF) [file pone.0068822.s004.pdf]

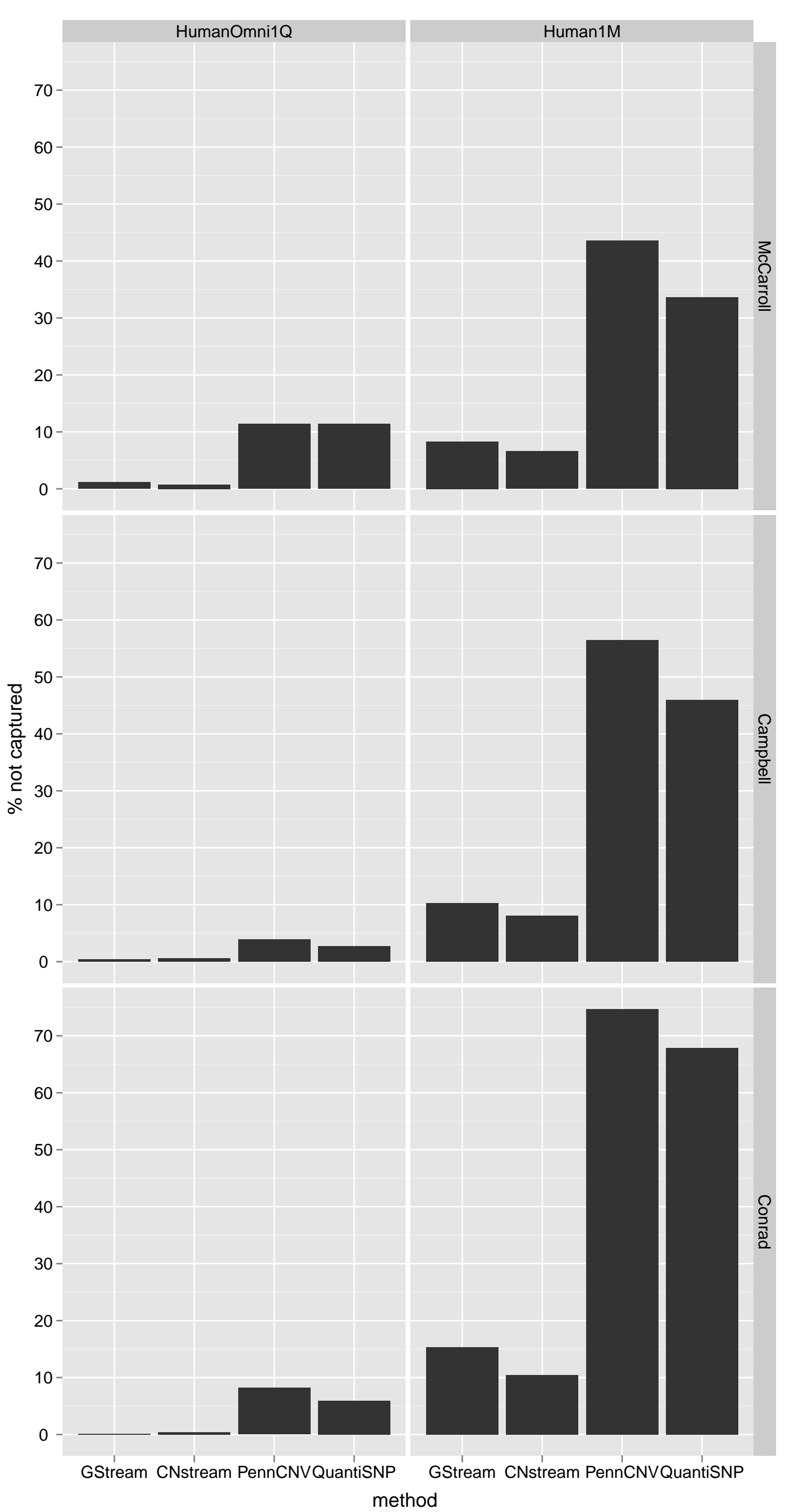

Supplement: Figure S6 — Missed associations. Percentage of associations (i.e. P-value<0.05 over the golden standard dataset) that were not captured by the different methods tested (i.e. P-value>0.05 over the tested method). (PDF) [file pone.0068822.s006.pdf]

HumanOmni1Q

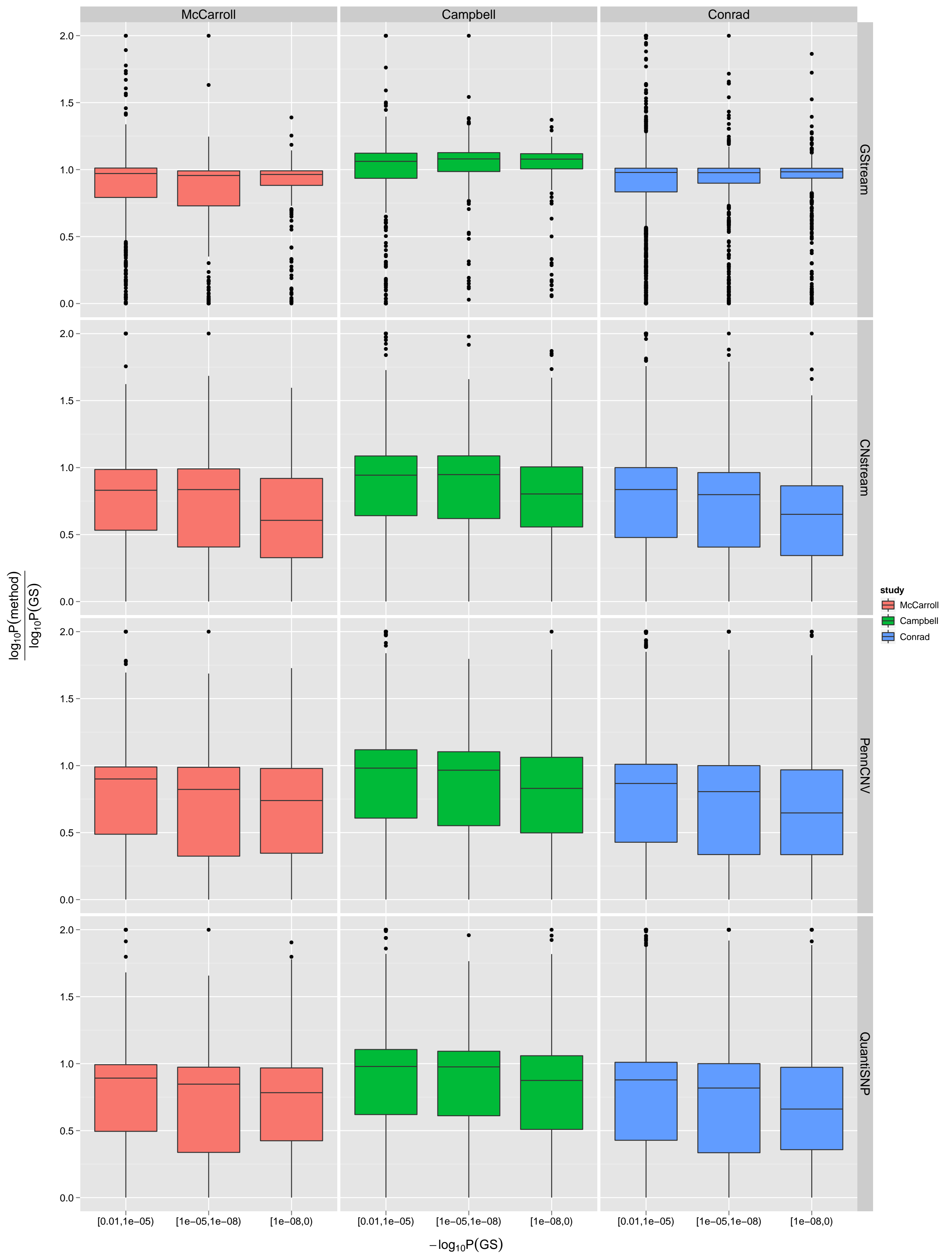

Supplement: Figure S7 — HumanOmni1-Quad P -value distributions. Distributions of the P-value association ratios depending on the golden standard dataset used for evaluation (i.e. represented by different colours) and on the P-value range obtained over the golden standard calls (i.e. horizontal axis). (PDF) [file pone.0068822.s007.pdf]

## Human1M

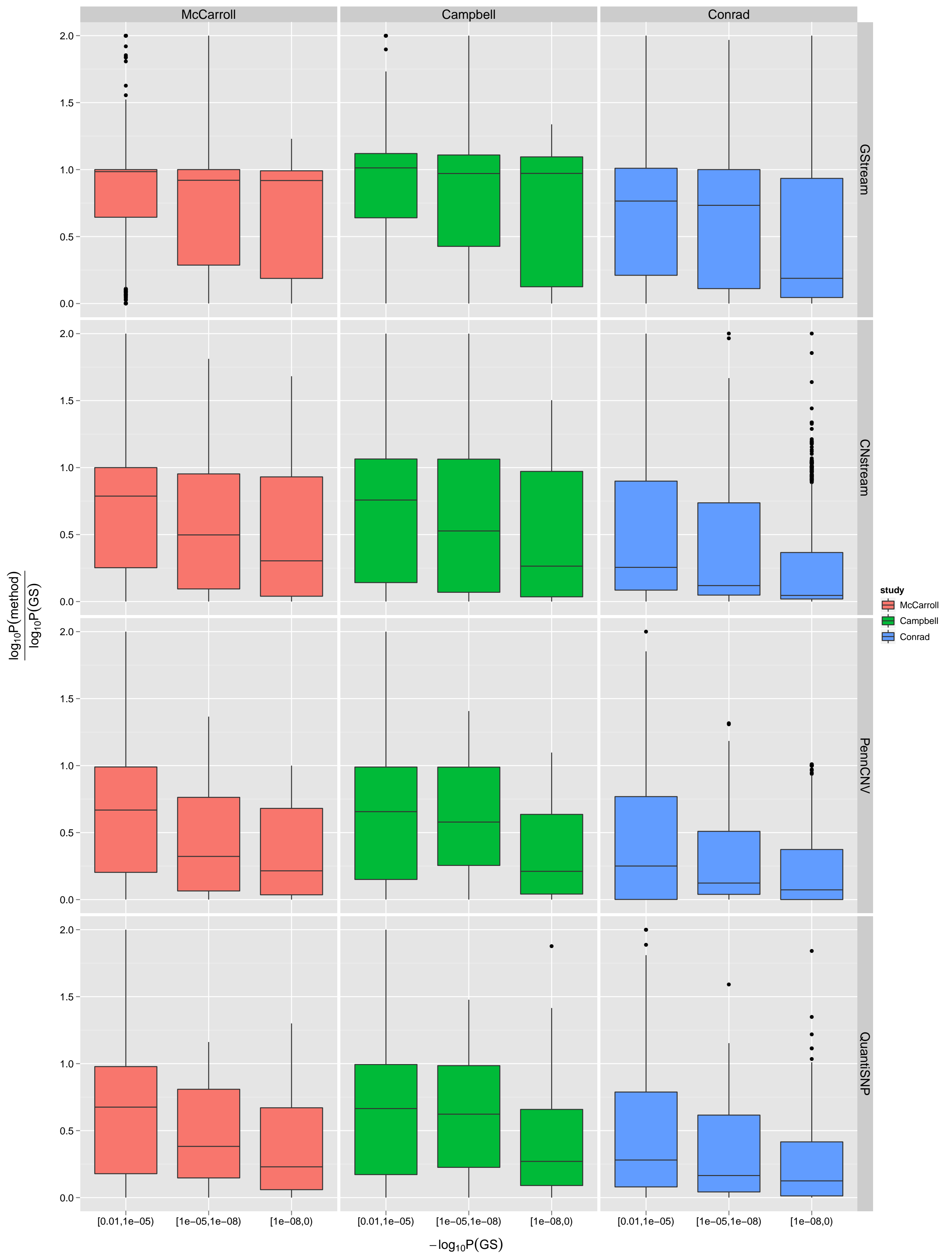

Supplement: Figure S8 — 1M-Duo P -value distributions. Distributions of the P-value association ratios depending on the golden standard dataset used for evaluation (i.e. represented by different colours) and on the P-value range obtained over the golden standard calls (i.e. horizontal axis). (PDF) [file pone.0068822.s008.pdf]

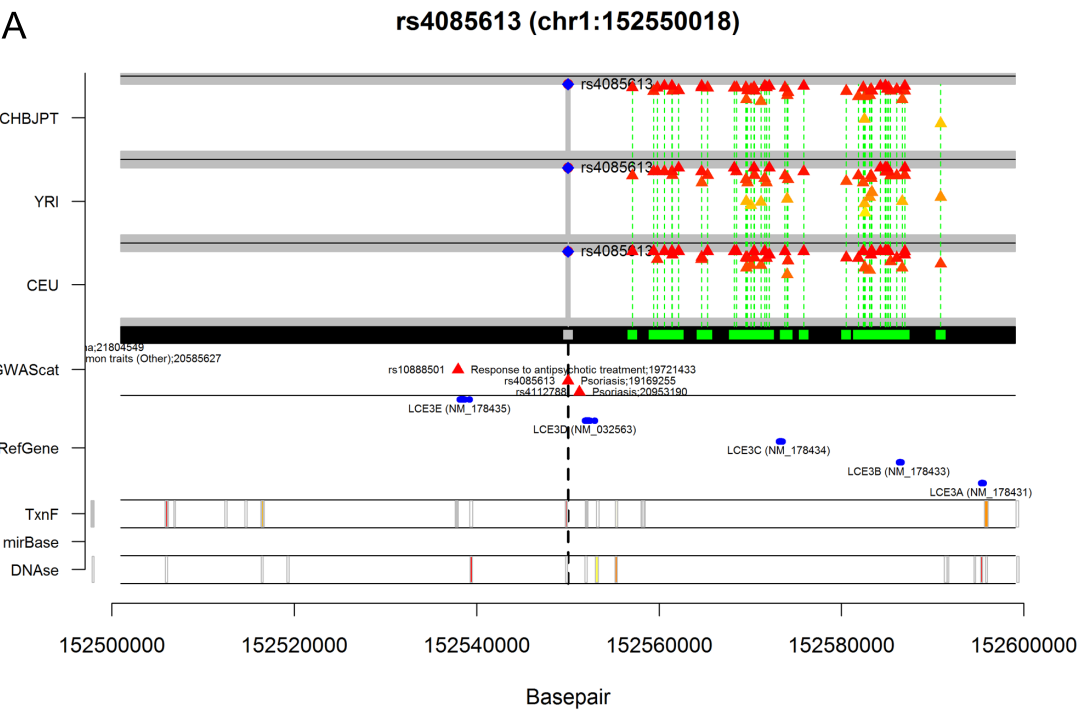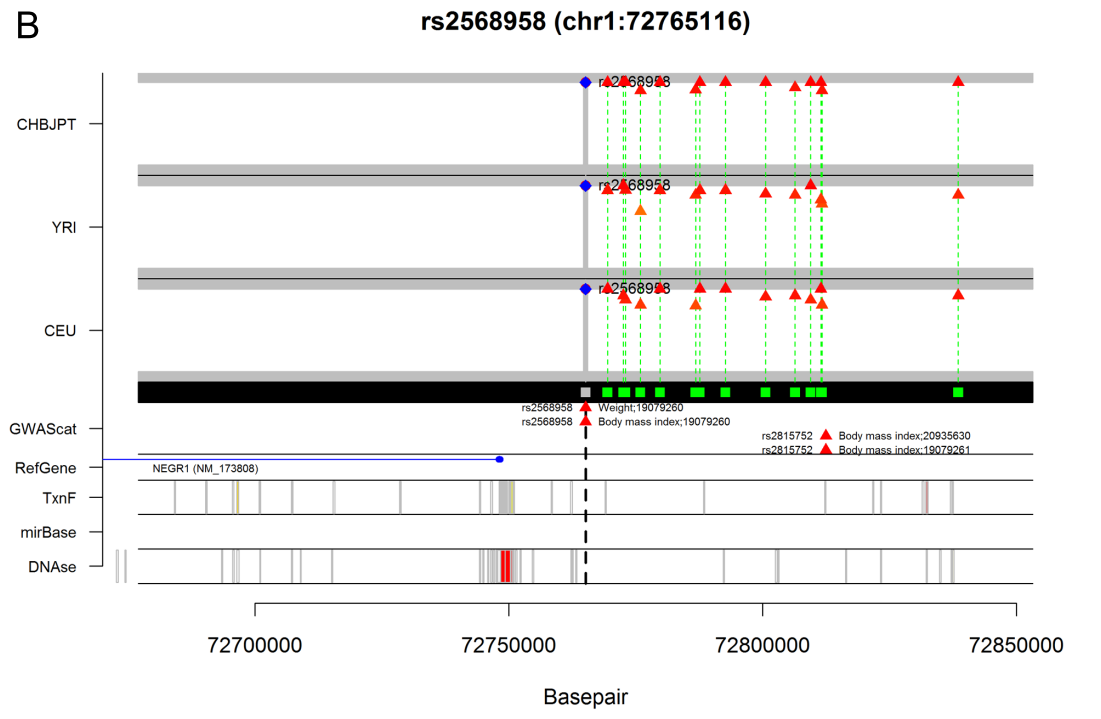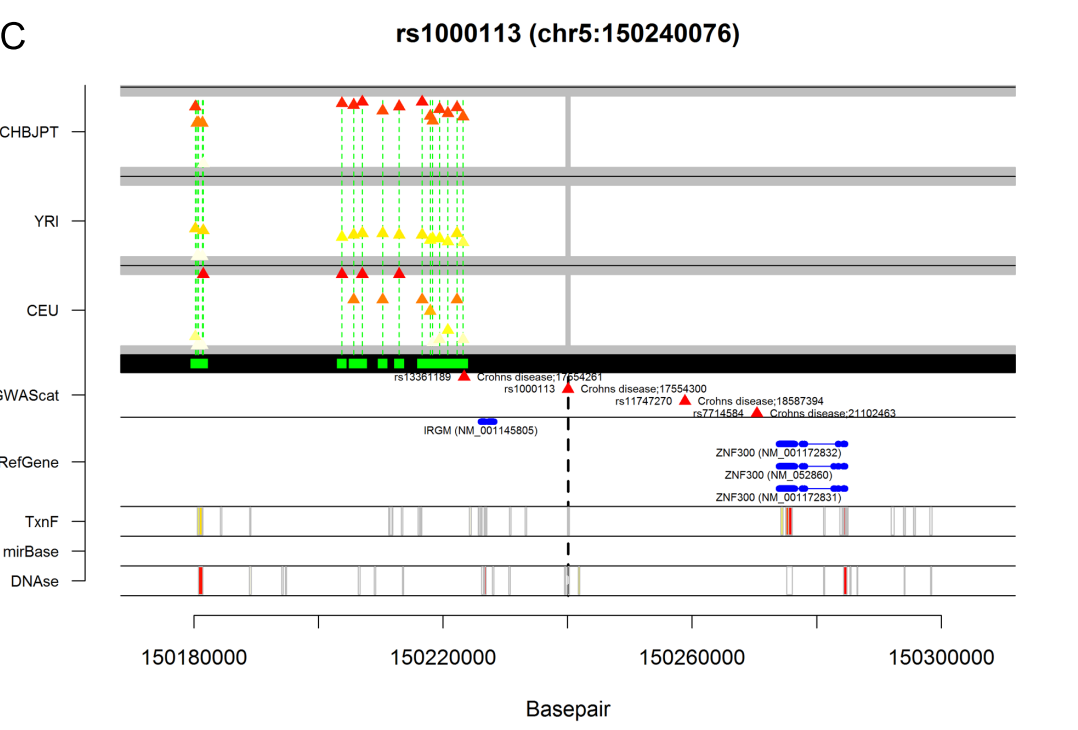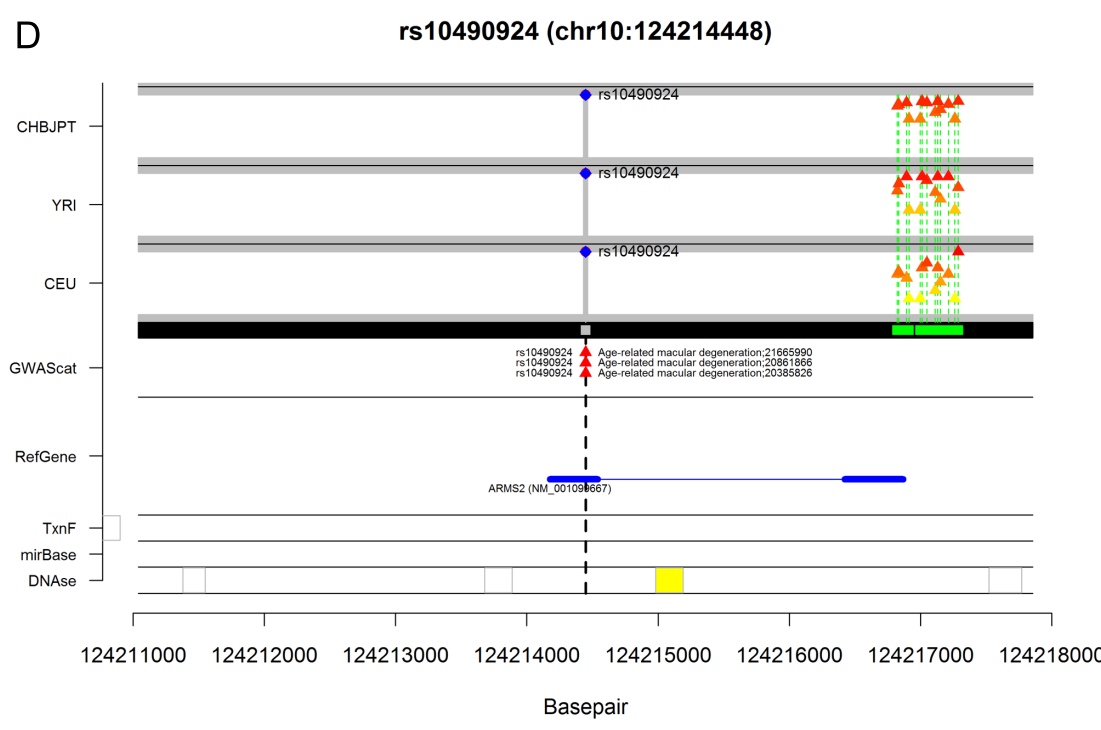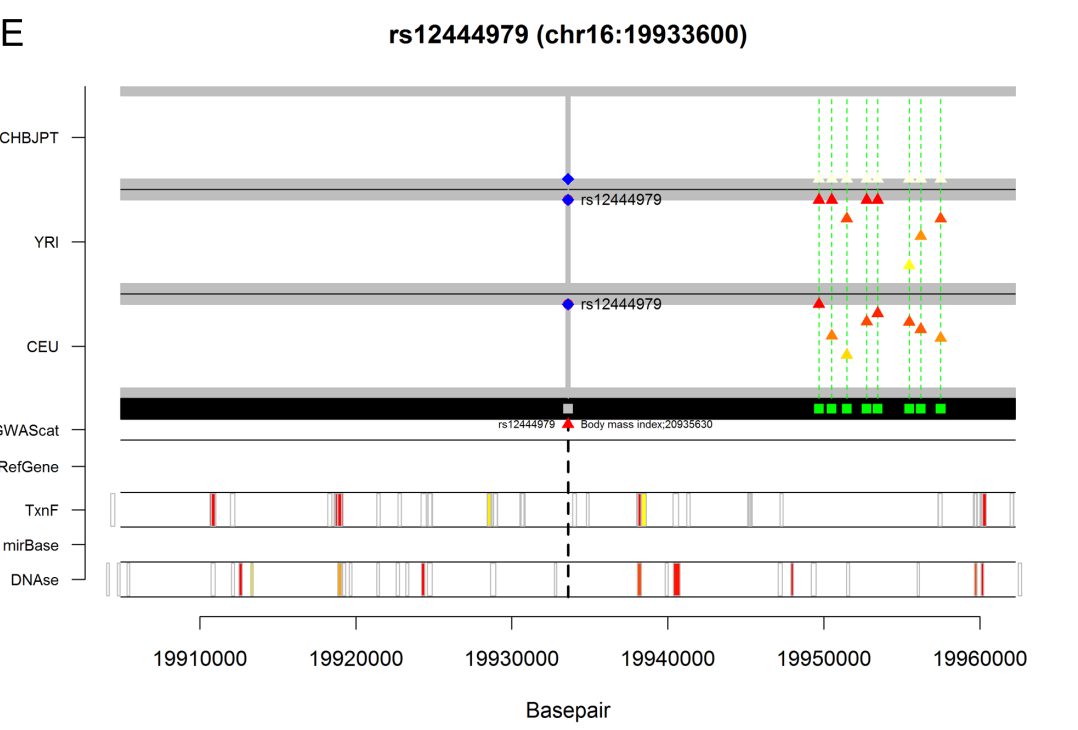

Supplement: Figure S9 — Previously reported CNV associations detected by LD analysis between GStream CNV genotypes and trait-associated SNPs. (A) LCE gene cluster deletion associated with Psoriasis risk. (B) NEGR1 deletion associated with body mass index. (C) IRGM deletion associated with Chrohn's disease. (D) ARMS2 deletion associated with age-related macular degeneration. (E) GPRC5B upstream deletion associated with body mass index. (PDF) [file pone.0068822.s009.pdf]

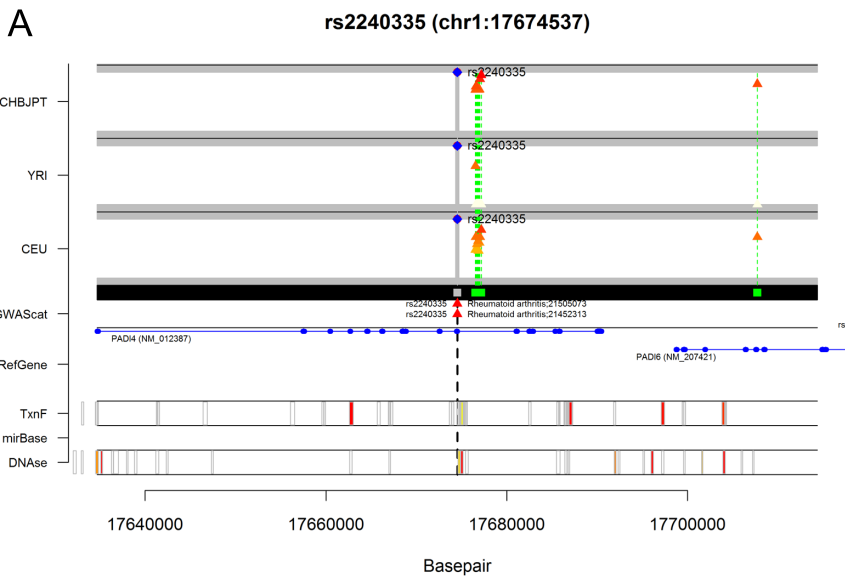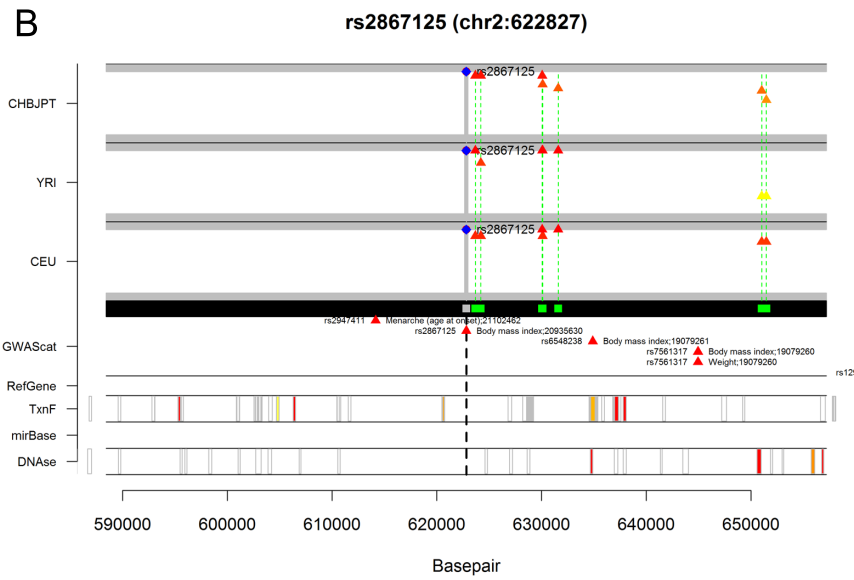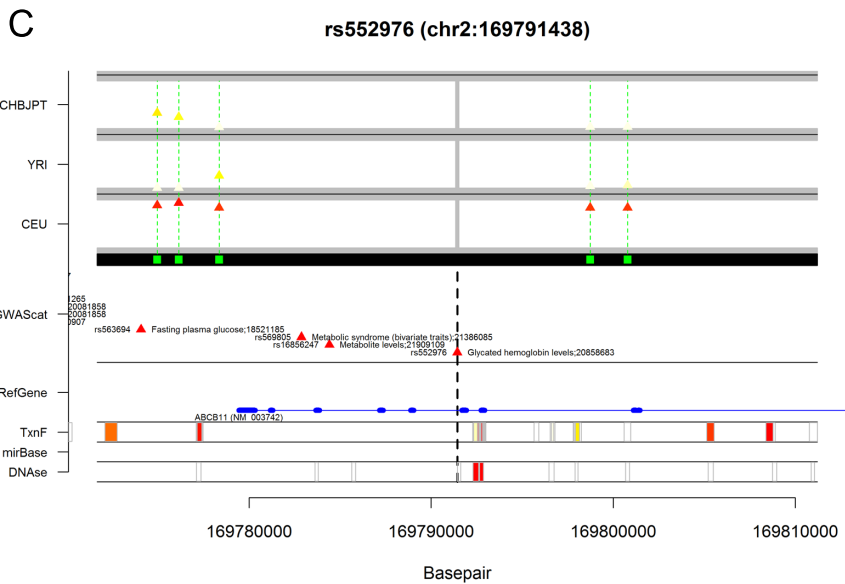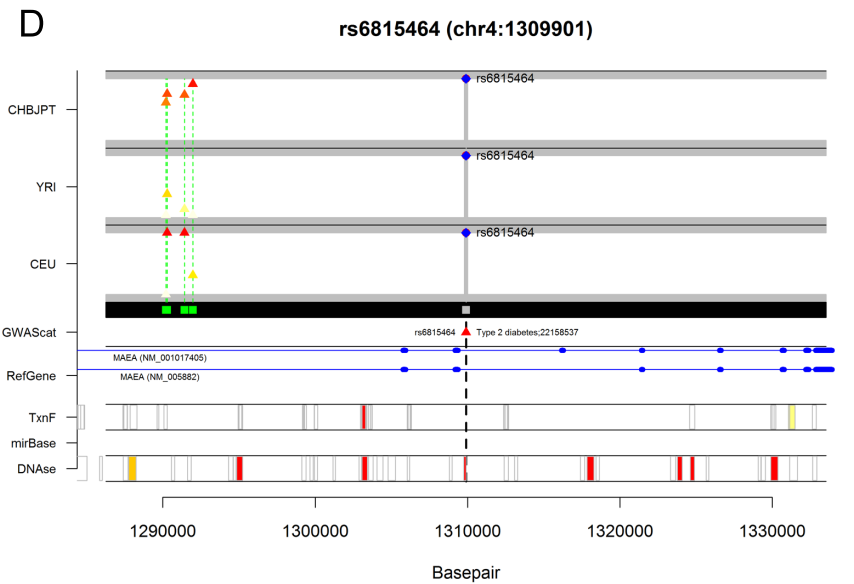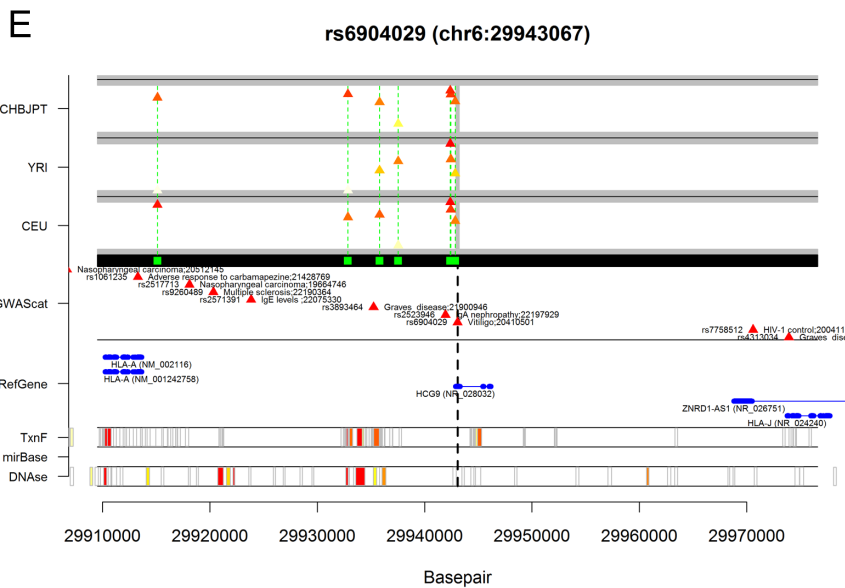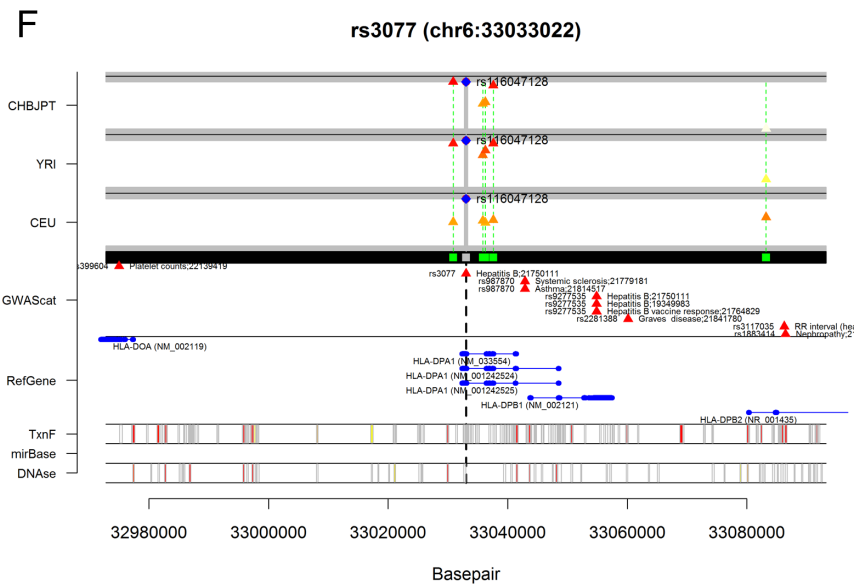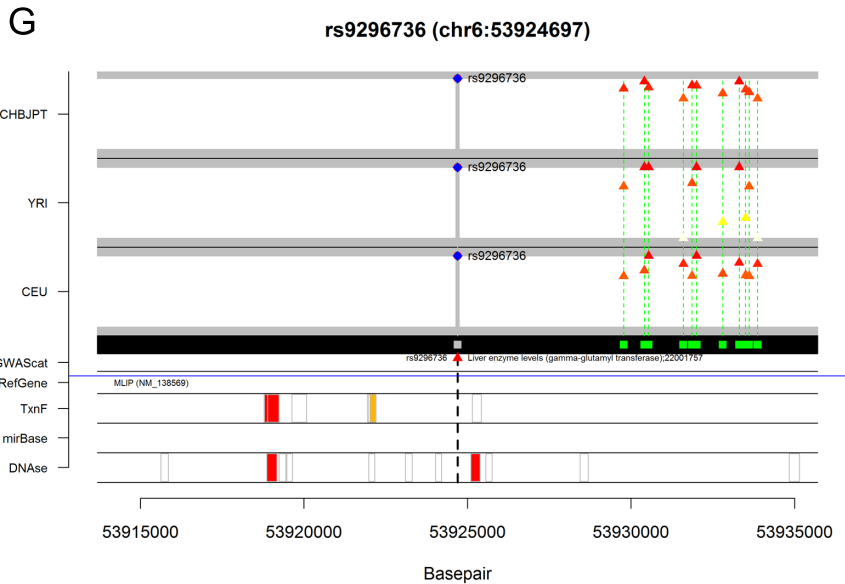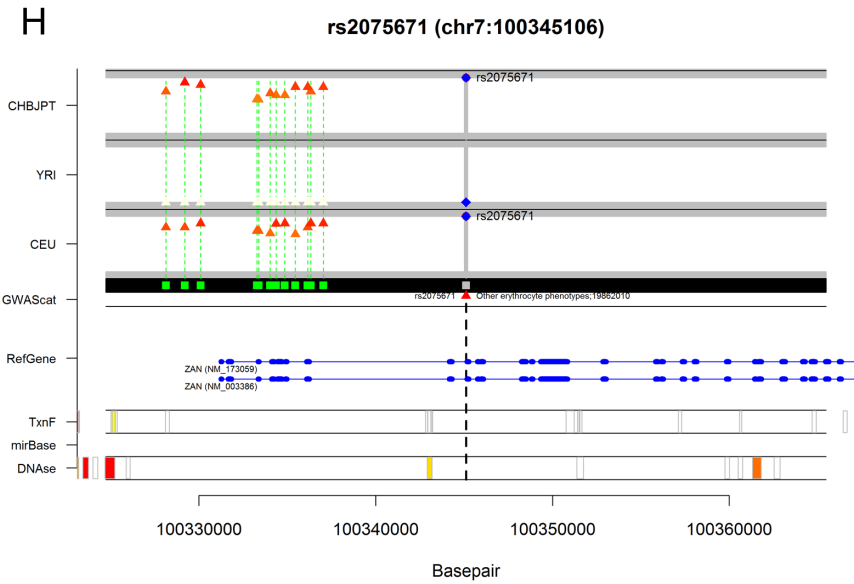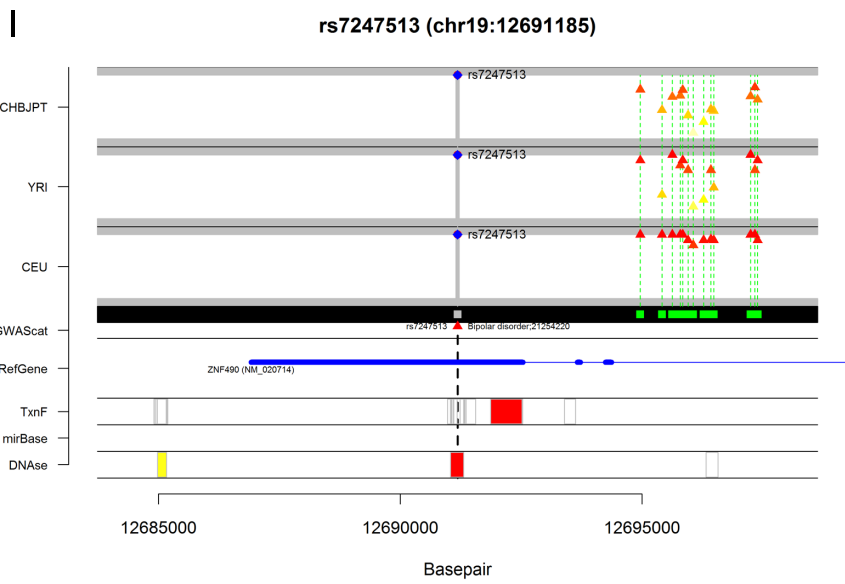

Supplement: Figure S10 — Interesting CNV associations detected by LD analysis between GStream CNV genotypes and trait-associated SNPs. (A) PADI4 gene deletion associated with Rheumatoid Arthritis. (B) TMEM18 downstream deletion associated with body mass index. (C) 3′-deletion of gene ABCB11 associated with glycated hemoglobin levels. (D) MAEA gene intron deletion associated with type 2 diabetes. (E) HCG9 deletion associated with Vitiligo. (F) HLA-DPA1 deletion associated with Hepatitis B. (G) MLIP intron deletion associated with liver enzyme levels. (H) ZAN gene deletion associated with red blood cell count. (I) ZNF490 intron deletion associated with bipolar disorder. (PDF) [file pone.0068822.s010.pdf]

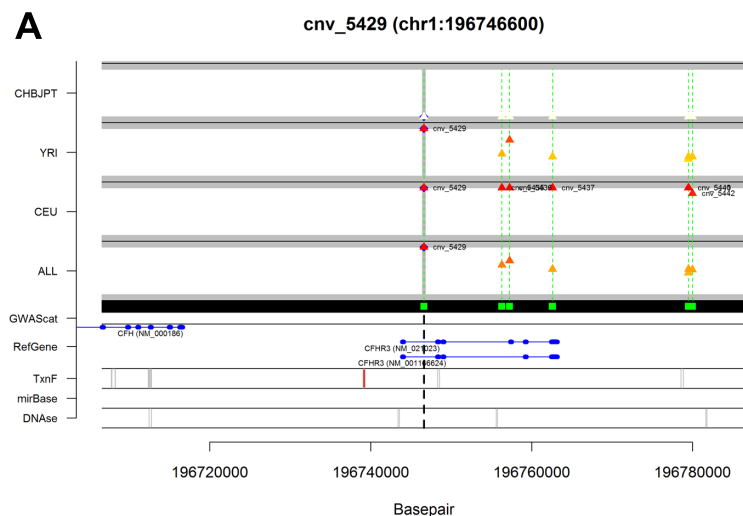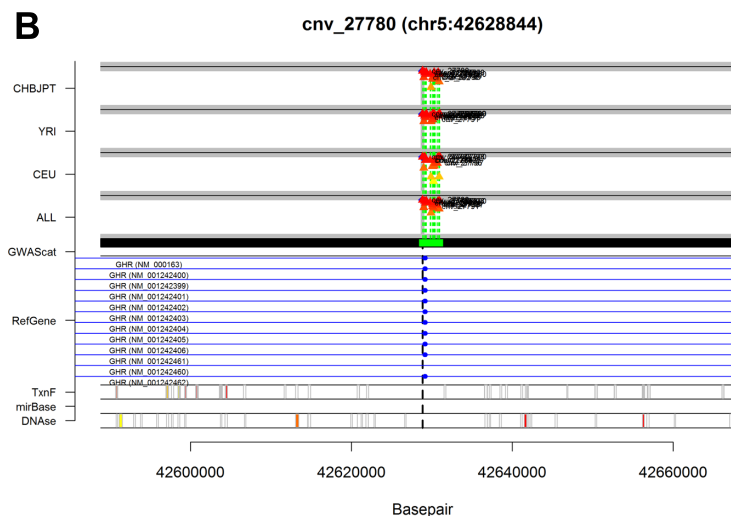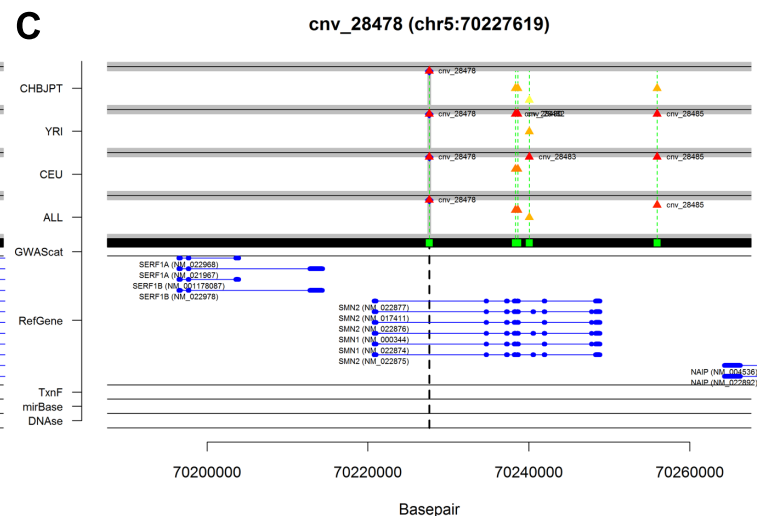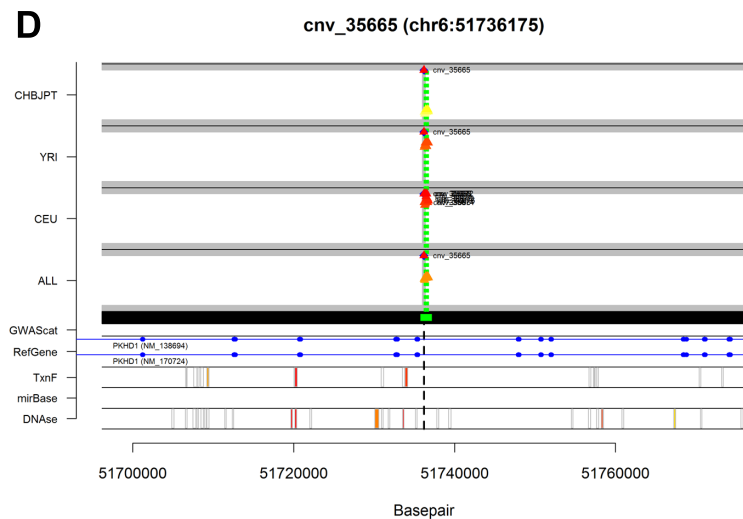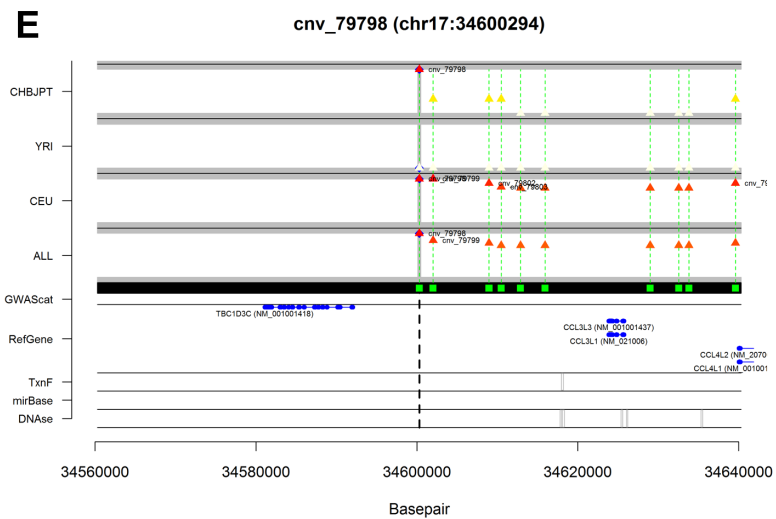

Supplement: Figure S11 — GStream detected CNP loci spanning disease-related genes (OMIM) where CNVs have been previously associated with disease. (A) CNP spanning CFHR1 and CFHR3 previously associated to age-related macular degeneration. (B) Deletion of GHR exon 3 that has been previously associated with increased responsiveness to growth hormone and Laron dwarfism. (C) Detected SMN gene deletion previously associated with spinal muscular atrophy. (D) PKHD1 deletion associated with polycystic kidney. (E) CCL3L1/CCL3L3 deletion previously associated with susceptibility to HIV/AIDS. (PDF) [file pone.0068822.s011.pdf]

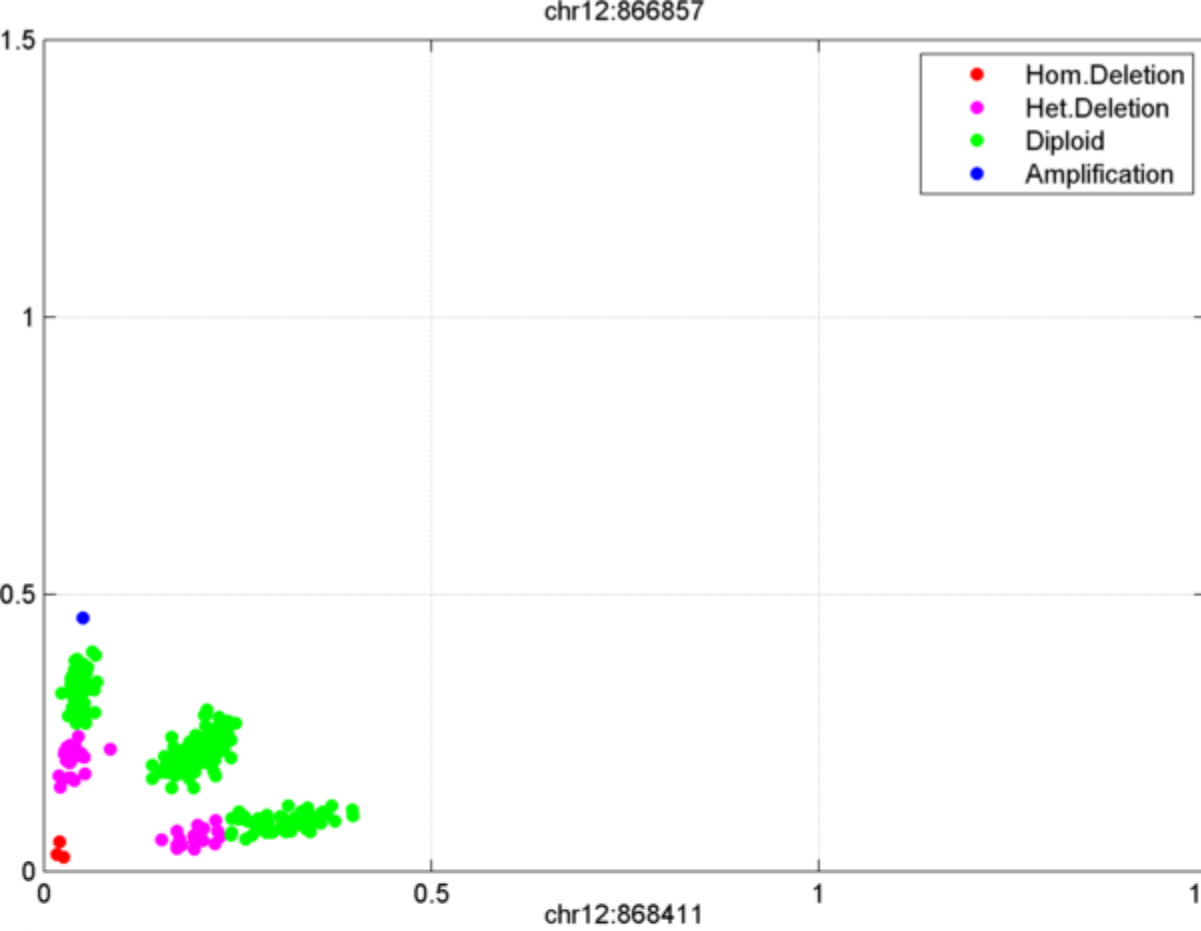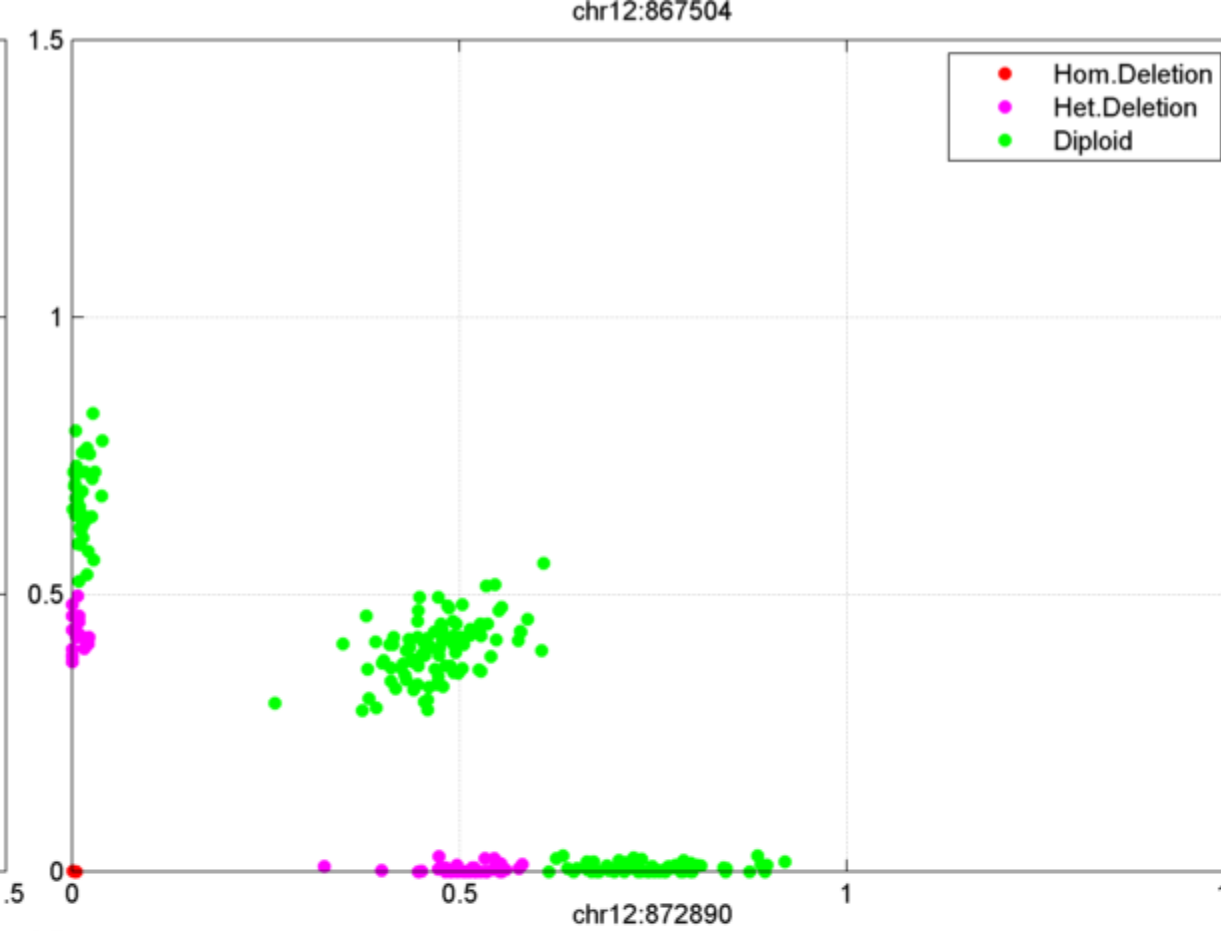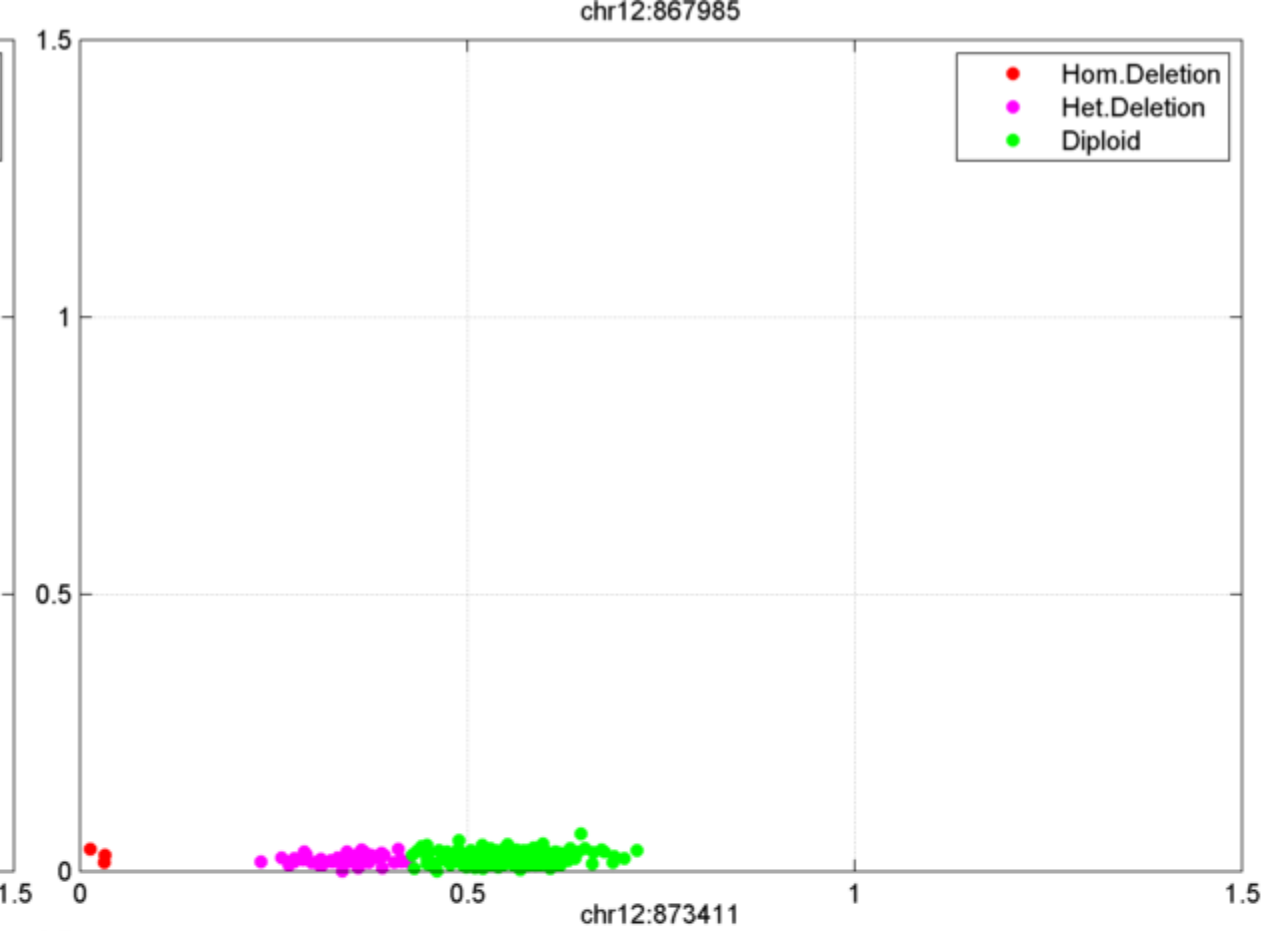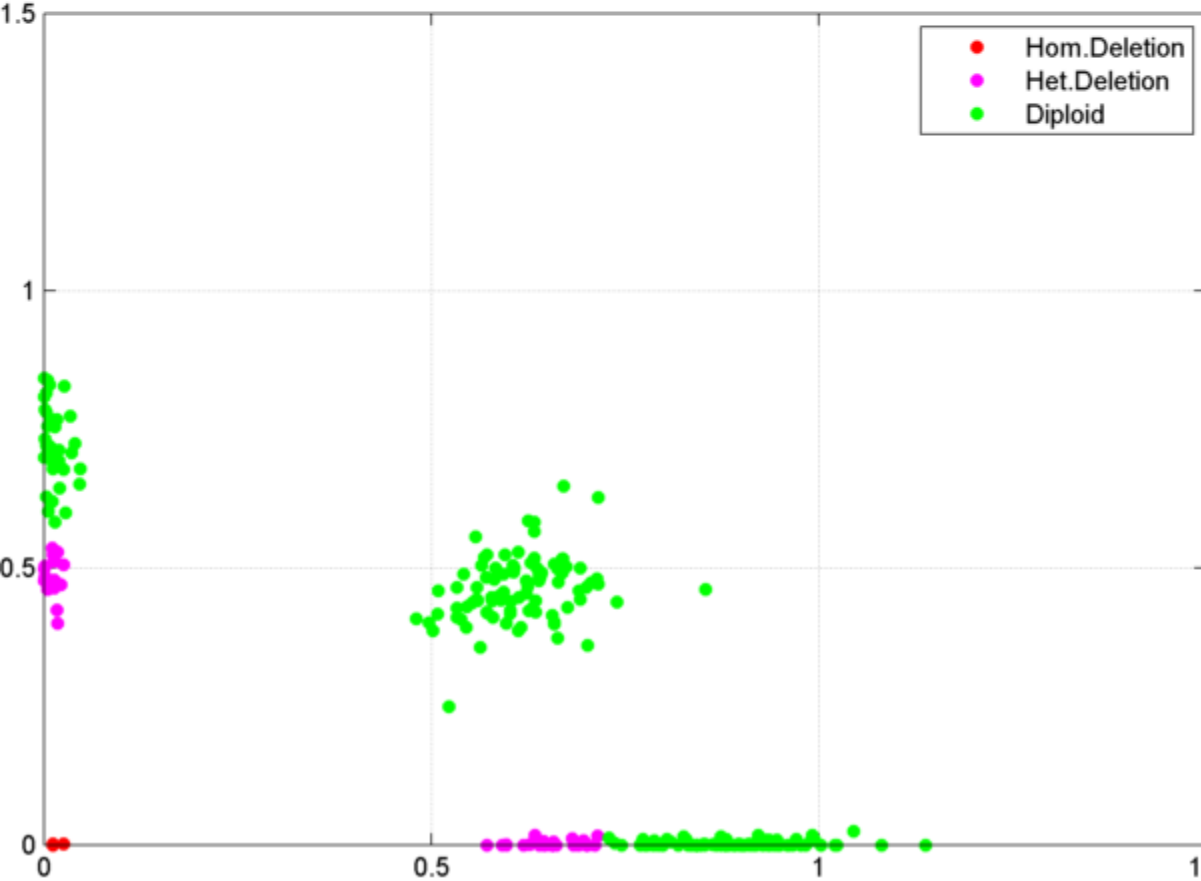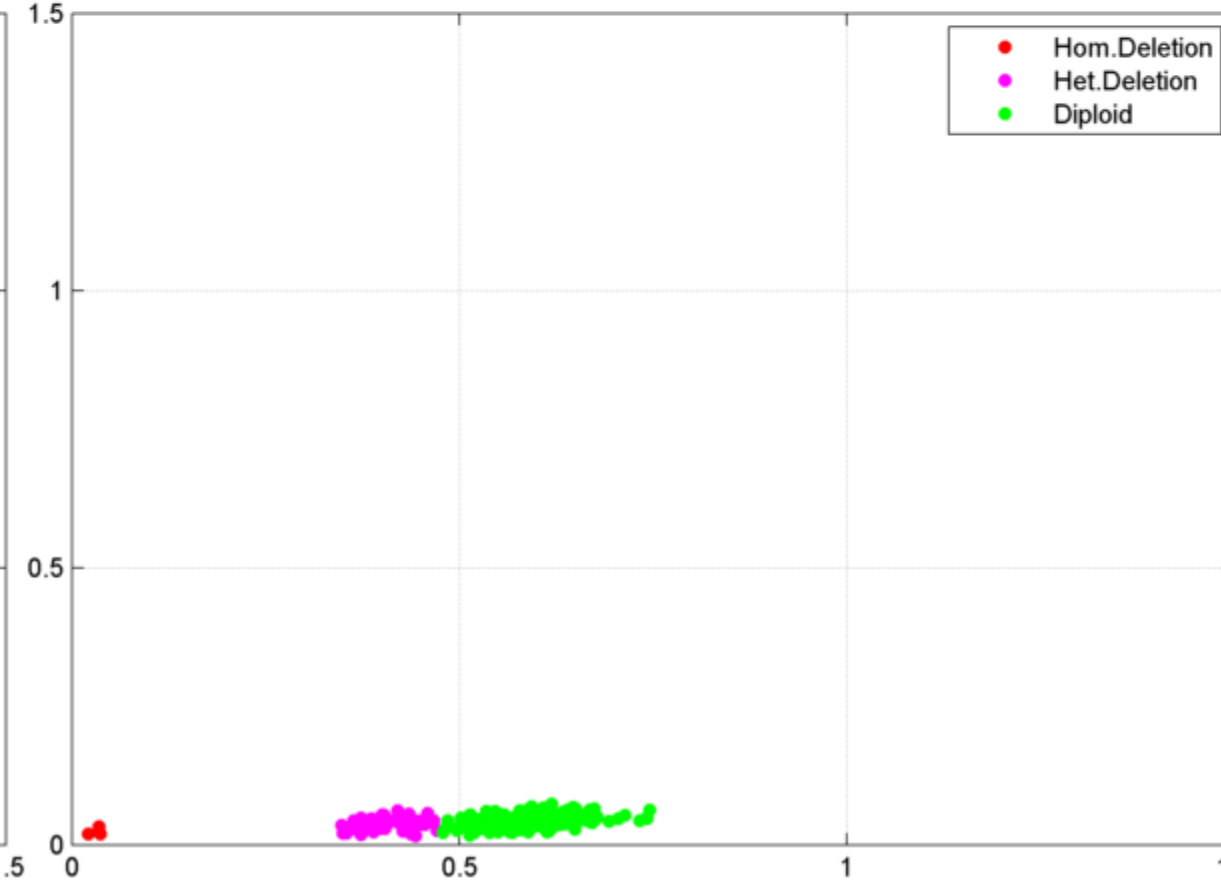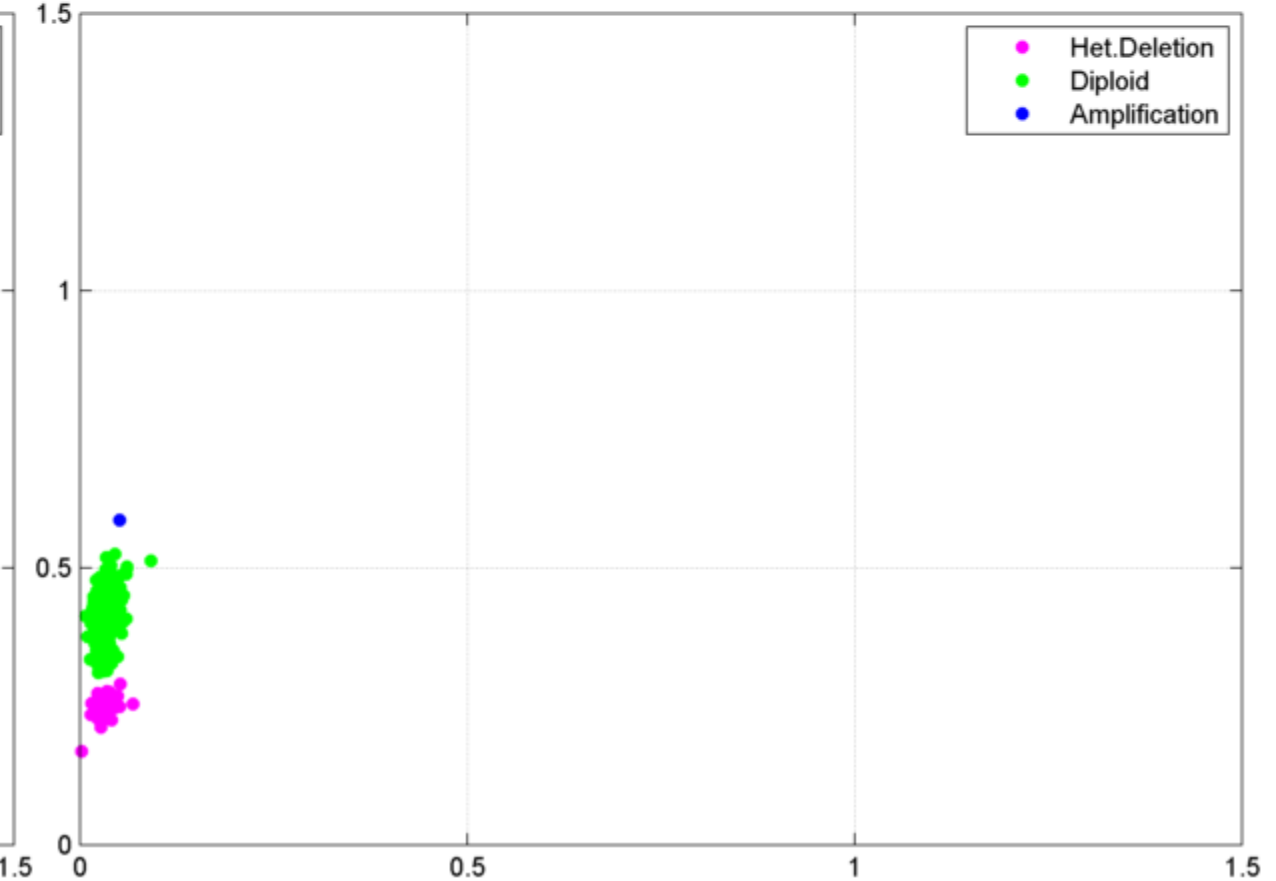

Supplement: Figure S12 — GStream calls across consecutive markers spanning the same CNV loci. These 6 microarray probes cover the same CNV loci but show very different CNV intensity patterns. GStream is completely adapted to these types of variations and its calling procedure is able to obtain very concordant calls when analyzing probes spanning the same CNV. (PDF) [file pone.0068822.s012.pdf]
