# Supplementary material for: GStream: Improving SNP and CNV Coverage on Genome-Wide Association Studies
Source: PLoS One. 2013 Jul 3;8(7):e68822. doi: 10.1371/journal.pone.0068822 (PMC3700900; doi:10.1371/journal.pone.0068822)
Supplement: Figure S5 — Microarray coverage density. Coverage density of each microarray platform over the CNV regions defined by each reference study. There are major differences between the first Infinum HD platforms (Human610-Quad and Human1M-Duo) and their succesors including specific CNV coverage (Human660W-Quad and HumanOmni1-Quad). Both Human610-Quad and Human1M-Duo have a mean number of ∼10 markers covering CNV regions, while Human660W was designed with a highest coverage (∼20 markers/region) for almost 50% of the regions. Finally, HumanOmni1-Quad increased the global coverage to ∼20 markers/region. (PDF) [file pone.0068822.s005.pdf]

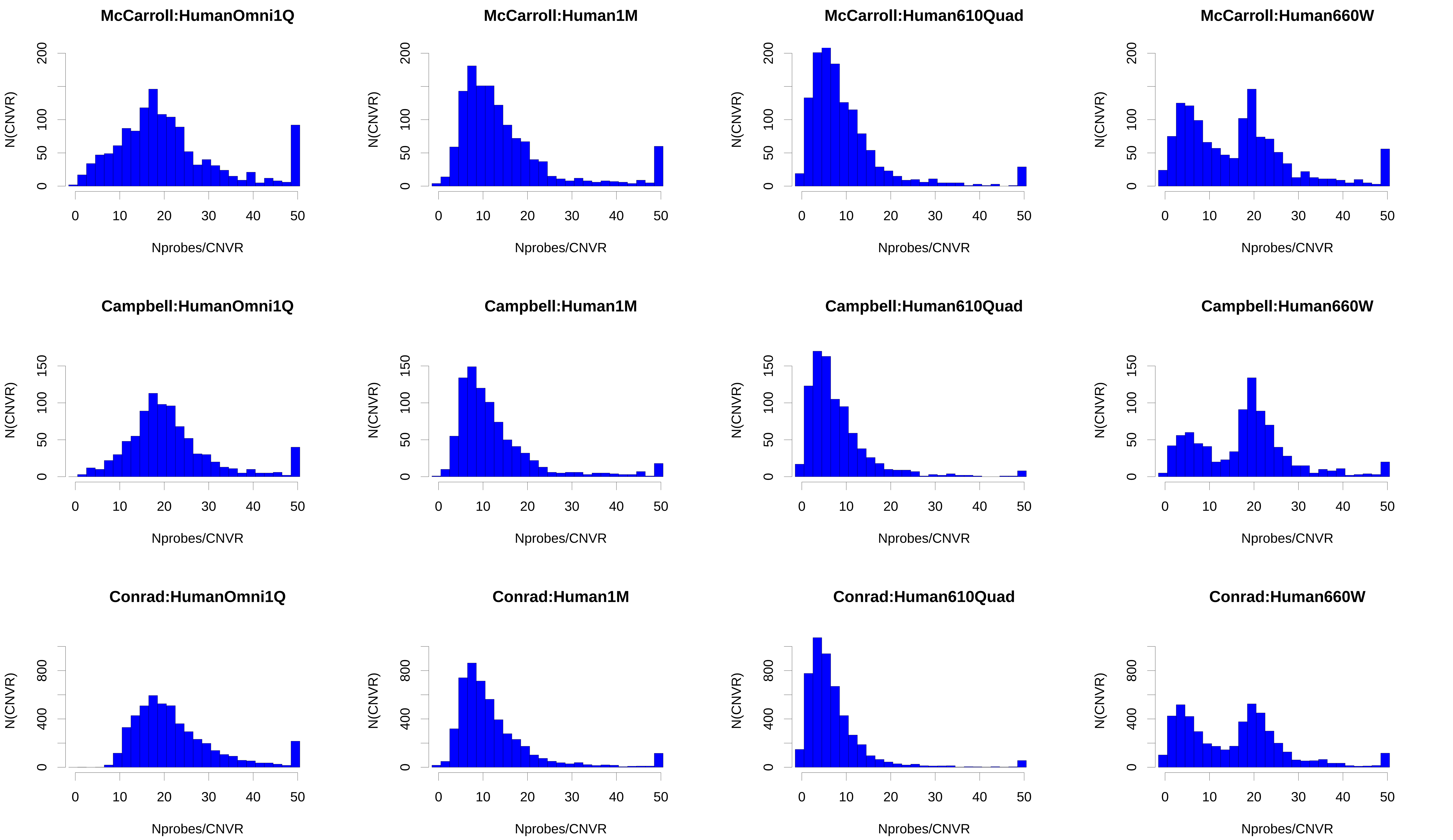
McCarroll:HumanOmni1Q
McCarroll:Human1M
McCarroll:Human610Quad
McCarroll:Human660W
Campbell:HumanOmni1Q
Campbell:Human1M
Campbell:Human610Quad
Campbell:Human660W
Conrad:HumanOmni1Q
Conrad:Human1M
Conrad:Human610Quad
Conrad:Human660W
N(CNVR)
Nprobes/CNVR
0
10
20
30
40
50
100
150
200
400
800
