# Supplementary material for: GStream: Improving SNP and CNV Coverage on Genome-Wide Association Studies
Source: PLoS One. 2013 Jul 3;8(7):e68822. doi: 10.1371/journal.pone.0068822 (PMC3700900; doi:10.1371/journal.pone.0068822)
Supplement: Text S1 — GStream algorithm details. (PDF) [file pone.0068822.s015.pdf]

GStream algorithm

INPUT DATA

The input data required by GStream is provided by a single file and corresponds to channel A and B intensities for each sample at each probe. Each line corresponds to one probe and columns one to three are reserved for the probe annotation data (name, chromosome and basepair). The following columns must contain channel A and B intensities for each sample. Therefore the expected number of columns is $N_{col} = 3 + 2N_S$, where N_S is the number of analyzed samples. We recall that channel A and B intensities are proportional to the number of copies of A and B alleles.

The input file can be easily generated from the Illumina GenomeStudio software by selecting the data fields required by GStream. Figure 1 shows the appropriate fields that have to be used to export GenomeStudio data to GStream.

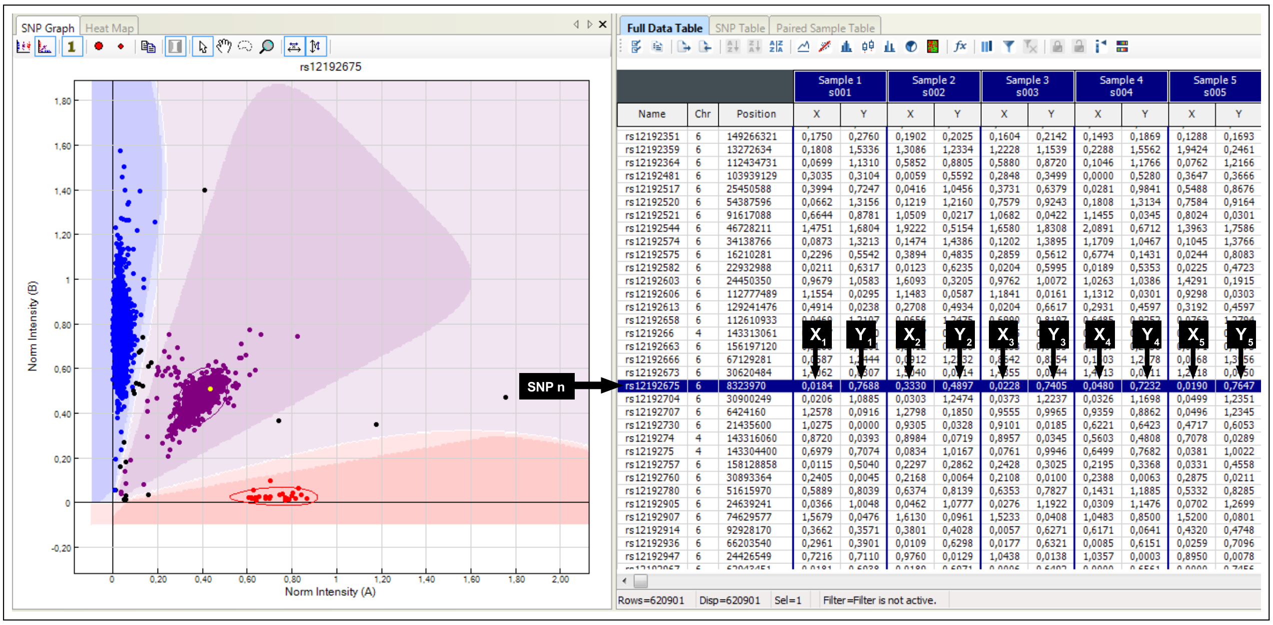

Figure 1: *GenomeStudio screenshot.* GenomeStudio data table showing the required columns for GStream analysis.

Since each probe is processed independently, the details on the algorithm will be specified for only one probe. From here on, channels A and B will be denoted as channel X and Y to avoid confusion between SNP genotypes (AA, AB and BB) and channel names. The following nomenclature will be used to refer to the input data:

- X_n: Channel X intensity for sample n at the processed SNP probe
- Y_n: Channel Y intensity for sample n at the processed SNP probe

The following sections explain in detail the algorithmic procedures integrated in GStream. These sections follow the processing workflow of GStream (fig. 2) and are subsequently and independently applied to each SNP probe:

- *Normalization:* This step aims to normalize channel X and Y intensity distributions in order to redress the sensibility bias between both channels.
- *SNP Genotyping:* This step has the objective of assigning a SNP genotype GT_n to each sample n. Samples are clustered in three main groups: AA homozygotes, AB heterozygotes and BB homozygotes ($GT_n \in AA, AB, BB$).
- *CNV Genotyping:* This step assigns a copy number score SC_n to each sample n.

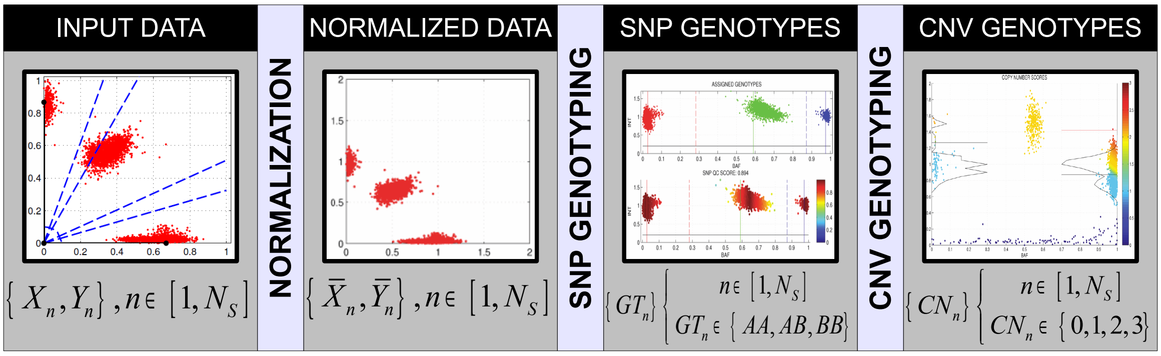

Figure 2: *GStream workflow.* This schema shows the three main stages of GStream method.

INTENSITY NORMALIZATION

This step performs channel intensity normalization in order to equalize both channel intensity distributions. Once this normalization step is performed, a coordinate transformation is applied to obtain the absolute intensities I_n and the allelic frequencies BAF_n. Channel intensity normalization is crucial since the sensibility differences of each SNP probe and channel can lead to bias affecting the genotyping performance.

First, in order to avoid numerical overflows raw channel intensities under a sensitivity threshold (T_S) are set to the minimum allowed value T_S:

$$X'_n = \begin{cases} T_S, & \text{if } X_n < T_S; \\ X_n, & \text{else.} \end{cases} \quad Y'_n = \begin{cases} T_S, & \text{if } Y_n < T_S; \\ Y_n, & \text{else.} \end{cases} \tag{1}$$

The following step consists of the identification the two candidate homozygote groups, containing the samples that have a high probability of being diploid homozygotes. The candidates must fulfill two conditions:

- Having high absolute intensities ($I_n = X'_n + Y'_n$).
- Having angular coordinates ($BAF_n = \frac{2}{\pi} \arctan \frac{Y'_n}{X'_n}$) next to 0 (AA homozygotes) or 1 (BB homozygotes).

$$n \in AA_{cand} \Leftrightarrow \begin{cases} I_n = X'_n + Y'_n > I_{min} \\ BAF_n < \theta_L \end{cases} \qquad n \in BB_{cand} \Leftrightarrow \begin{cases} I_n = X'_n + Y'_n > I_{min} \\ BAF_n > 1 - \theta_L \end{cases} \tag{2}$$

Once candidate samples are detected (fig. 4A), the algorithm applies different procedures to compute the scaling factors depending on the candidate groups identified:

- AA and BB candidates found If both candidate groups have been detected, the algorithm independently computes the scaling factors α_X and α_Y of each channel. These scaling factors correspond to the maximums of their respective candidate intensity-weighted histograms H_W (fig. 3) which are computed as follows:

$$\begin{gathered} S(m') = \frac{m'}{10} \max_{n \in AA_{cand}} (I_n) \ , \ m' \in [0 \dots 10] \\ W(m) = 0.5 * (S(m) + S(m+1)), m \in [0 \dots 9] \\ H_W(m) = W(m)^3 * \textstyle\sum_{n=1}^{N_S} F_I(n \in AA_{cand}) * F_I(S(m) < I_n \leq S(m+1)) \\ \alpha_X = W(\underset{m}{argmax}(H_W(m))) \end{gathered} \tag{3}$$

  where $F_I(expr) = 1 \leftrightarrow expr = True$,else $F_I(expr) = 0$. α_Y is computed in the same way but changing $AA_{cand} \rightarrow BB_{cand}$. $S(m)$ is a vector containing the centers of the intervals used to compute the histogram and $W(m)$ the weighting factor. α_Y is computed in the same way but changing $AA_{cand} \rightarrow BB_{cand}$ within eq.3.
- Only one candidate group found If only the candidate AA homozygote group is detected (resp. BB), a peak detection algorithm is applied over the BAF histogram. If only one peak is detected, the algorithm assumes that the probe does not capture allelic variation and only detects AA homozygote samples (resp. BB). In that case, both channels are normalized using the corresponding AA homozygote scaling factor α_X (resp. α_Y) as computed in the previous section. If more than one peak is detected the algorithm assumes that peaks correspond to AA homozygote and AB heterozygote clusters (resp. BB and AB). α_X (resp. α_Y) is computed as in the previous section and $\alpha_Y = \alpha_X R_{xy}$ (resp. $\alpha_X = \alpha_Y R_{yx}$) where R_{xy} corresponds to a cross-sensibility ratio computed from the heterozygote sample intensities $R_{xy} = \frac{\overline{Y}_{het}}{\overline{X}_{het}}$ ($R_{yx} = R_{xy}^{-1}$).

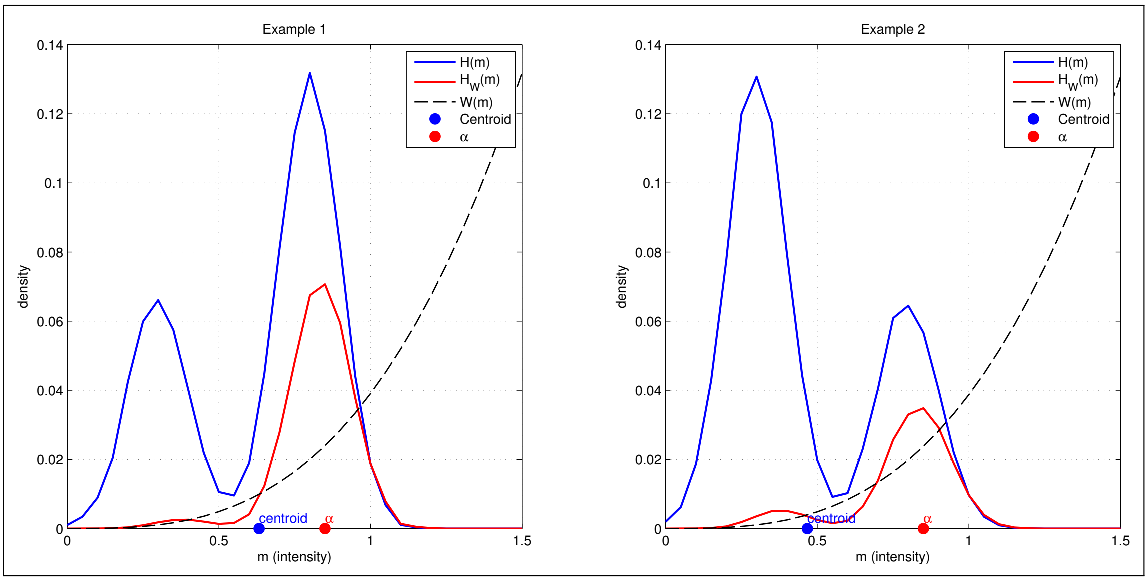

Figure 3: *Weighted intensity histogram.* In this figure two candidate homozygote intensity distributions are shown with a typical CNV pattern. The most frequent component corresponds to the two-copy component (higher intensity, left side), while the right side corresponds to the one-copy component. The weighted intensity histogram approach obtains scaling factors corresponding to the average intensity of the two-copy samples.

- <u>Neither AA nor BB candidates found</u>

  This case is uncommon. α_X and α_Y are computed as follows:

$$\alpha_X = \alpha_Y = \frac{1}{N} \sum_{n=1}^{N_S} I_n \tag{4}$$

Once scaling factors α_X and α_Y are found, channel intensities are scaled and new BAF and I coordinates are computed (fig.4B):

$$\begin{array}{lcl} X'_n = X'_n/\alpha_X & & I_n = X'_n + Y'_n \\ & \rightarrow & \\ Y'_n = Y'_n/\alpha_Y & & BAF_n = \frac{2}{\pi} \arctan \frac{Y'_n}{X'_n} \end{array} \tag{5}$$

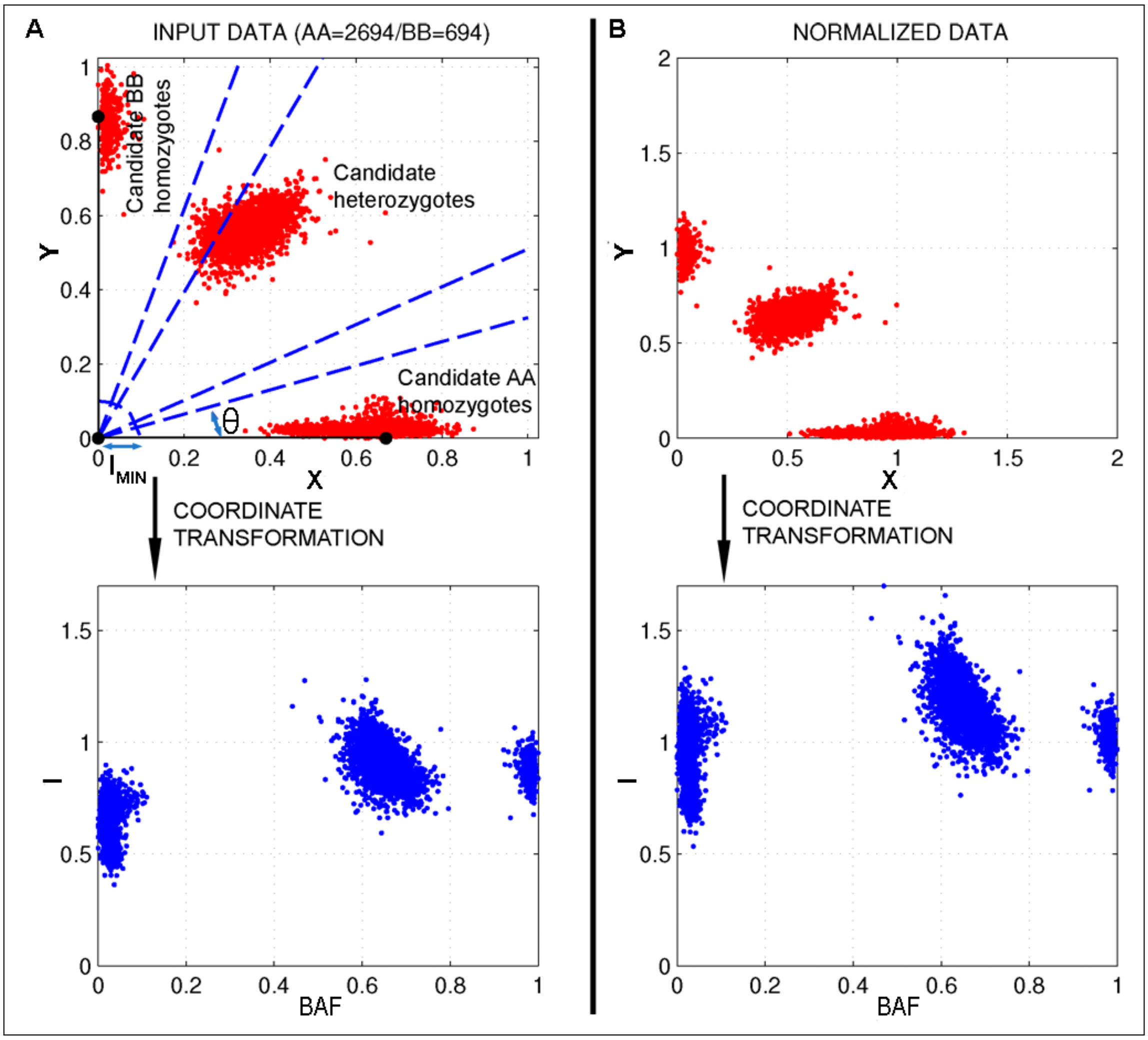

Figure 4: *Normalization.* (A) This figure shows an example on how homozygote candidates are detected depending on the I_{min} and θ parameters. (B) Shows the resulting intensities after normalization. The intensity differences between AA and BB homozygotes have been corrected and normalized to one.

SNP GENOTYPING ALGORITHM

Once intensities have been normalized, GStream identifies the clusters corresponding to each SNP genotype (AA, AB and BB). In this stage, specific CNV probes from Illumina microarrays are processed as the conventional SNP probes, with the singularity of commonly having only one genotype cluster. The genotyping algorithm is divided into the following steps:

- Zero detection: Absolute intensities (I_n) are used to detect homozygous deletion samples (0 alleles) characterized by low intensities at both channels (fig. 5). To do that the algorithm sorts the intensities in ascending order and computes the averaged derivative of the resulting

vector:

$$j = f(n)\ |I_j \leq I_{j+1}\ ,\ \ \forall n \in [1 \dots N_S]$$

$$D(j) = I_{j+1} - I_j \tag{6}$$

$$\overline{D}(j) = \frac{D(j-1)+D(j)+D(j+1)}{3}$$

Only sample intensities below the samples intensity average are taken into this calculation. Once $\overline{D}(j)$ has been computed, the second stage consists of identifying its maximum and verifying if it exceeds a predetermined threshold (T_{min}). If true, the zero threshold T_0 is fixed to its corresponding intensity value:

$$T_0 = \begin{cases} 0.5 * (I_{argmax(\overline{D}(j))} + I_{1+argmax(\overline{D}(j))}, & \text{if } max(\overline{D}(j)) > T_{min}; \\ 0, & \text{else.} \end{cases} \tag{7}$$

All the samples with an intensity below T_0 are excluded from the following analyses and genotyped as homozygous deletions ($GT_n = 0$ y $SC_n = 0$).

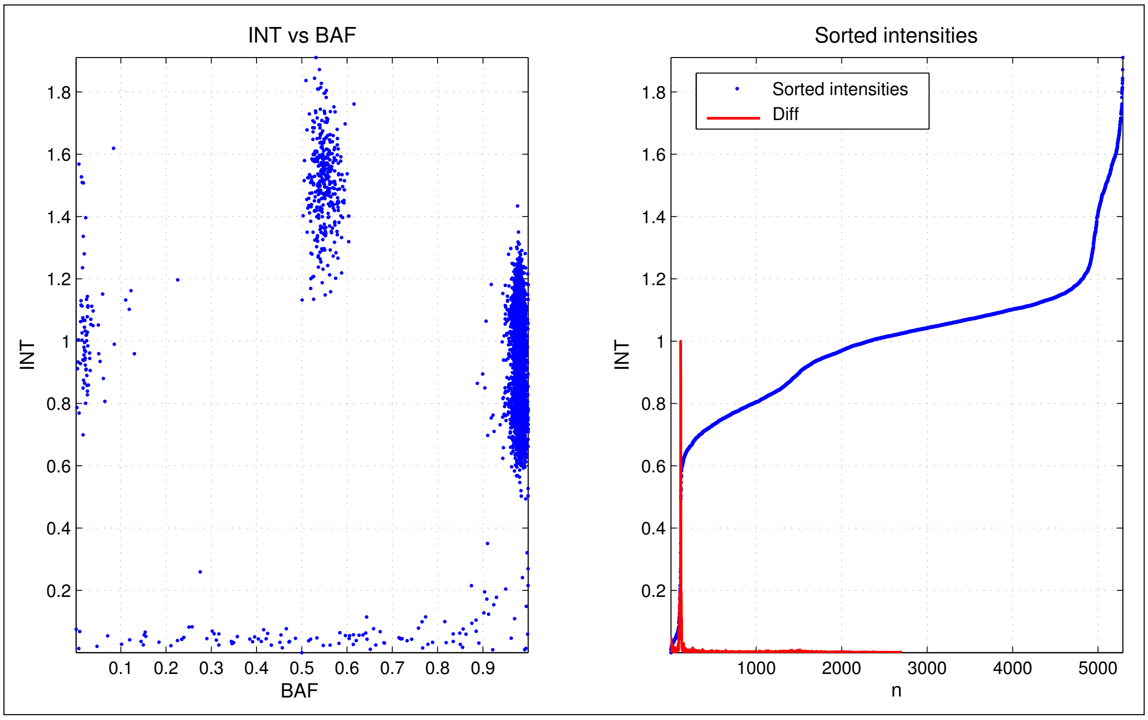

Figure 5: *Zero detection.* (A) Shows sample BAF and absolute intensity distribution within a probe where homozygote deletions can be observed. (B) Absolute intensities are sorted and peaks are detected over its averaged derivative. If the peak exceeds a threshold, the corresponding intensity will determine the homozygous deletion intensity threshold.

- Limit detection between genotypes: The allele frequency (BAF_n) probability density function (PDF) is estimated computing its histogram $H_{BAF}(m)$. The histogram resolution N_{res} (number of bins) is adjusted depending on the number of analyzed samples between the range $20 < N_{res} < 40$. Once the PDF has been estimated, the maximum peak corresponding to an

homozygote cluster is identified ($m_p(1)$). In order to label a peak to an homozygote cluster it must be located at allelic frequencies next to 0 (AA) or 1 (BB). From this peak, a sliding window with a predefined length ($L = \frac{N_{res}}{2}$) is applied over the PDF until another value within the window exceeds the first window value at a predefined distance d_p. When this condition is reached, the BAF value corresponding tho the PDF minimum within the window is set as a genotype limit L_g. This algorithm is applied iteratively until two limits are fixed ($L_{AA|AB}$ y $L_{BB|AB}$) or all the BAF range ($[0, 1]$) has been covered (fig. 6A). The following pseudocode resumes the algorithm:

$H_{BAF}(m),\ m \in [0, 1, ...N_{res}];$ — histogram
$m_p(1) = \underset{m}{\operatorname{argmax}}(H_{BAF}(m));$ — maximum homozygote peak
$i_{peak} = 2;$
$while(i_{peak} < 3),$ — condición stop 1
    $for(i = (m_p + 1) : N_{res}),$ — sliding window starting point
        $m_i = \underset{m \in [i...i+\frac{N_{res}}{2}]}{\operatorname{argmax}} (H_{BAF}(m))$ — maximum within the window
        $if(m_i - m_p(i_{peak} - 1) >= d_p),$ — minimum distance to assign a new genotype peak
            $m_p(i_{peak}) = m_i;$ — new peak position
            $i_{peak} = i_{peak} + 1;$
            $Lg_{i_{peak}-1} = \underset{m \in [m_p(i_{peak}-1)...m_p(i_{peak})]}{\operatorname{argmin}} (H_{BAF}(m))$ — genotype limit
            $break;$ — search new peak
        end
        $if(i == N_{res}),\ break;\ end$ — condición stop 2
    end
end

The limits between genotypes determine the BAF ranges assigned to each genotype and each sample will be genotyped accordingly (fig. 6B):

$$GT_n = \begin{cases} 0, & \text{if } I_n < T_0; \\ 1, & \text{if } I_n \geq T_0 \ \ \& \ \ BAF_n < L_{AA|AB}; \\ 2, & \text{if } I_n \geq T_0 \ \ \& \ \ L_{AA|AB} \leq BAF_n < L_{BB|AB}; \\ 3, & \text{if } I_n \geq T_0 \ \ \& \ \ BAF_n \geq L_{BB|AB}. \end{cases} \tag{8}$$

- Re-Genotyping: If the number of detected clusters is less than three, each cluster is reanalyzed with a better resolution (increasing the number of bins used for the PDF estimation) with the purpose of identifying subclusters corresponding to different genotypes. This method avoids common errors seen in other algorithms where, for example, the genotypes corresponding to probes with high discordant sensibilities between channels are incorrectly assigned.

- Scoring: Finally, a probe quality score and an individual sample genotyping score are computed (fig. 6C). The global score is proportional to the average standard deviation between the BAF values assigned to each genotype, while the individual scores correspond to the distance between BAF sample value and its assigned genotype cluster center normalized by the distance between genotype cluster centers.

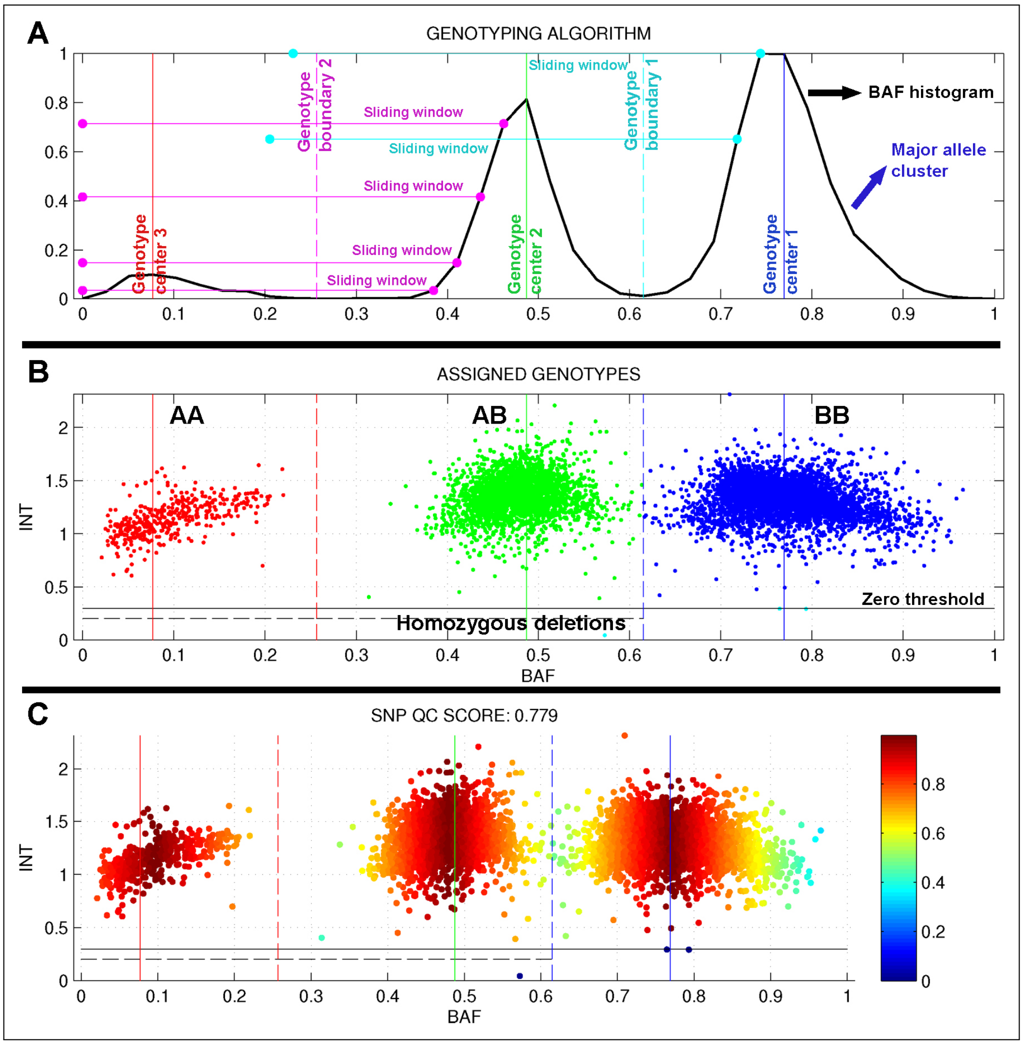

Figure 6: *Limit detection between genotypes.* (A) Limit detection algorithm over the BAF probability density function. (B) Resulting genotypes assigned by GStream. (C) Sample genotype scores.

CNV GENOTYPING ALGORITHM

GStream uses normalized intensities and SNP genotypes computed in the SNP genotyping stage to identify the presence of deletions and amplifications. These variations are characterized by variable clustering patterns on the intensity probe data (i.e. high frequency CNVs) or by slight deviations from the diploid distribution (i.e. low frequency CNVs).

One of the improvements incorporated by the algorithm is that each SNP genotype cluster is independently analyzed, taking only into account the intensity channel that carries valuable information. This way, the CNV algorithm is divided in four parallel steps (fig. 7):

- Analysis of channel A intensities from the samples genotyped as AA homozygotes ($X'_{n|GT_n=AA}$).
- Analysis of channel B intensities from the samples genotyped as BB homozygotes ($Y'_{n|GT_n=BB}$).
- Analysis of channel A intensities from the samples genotyped as AB heterozygotes ($X'_{n|GT_n=AB}$).
- Analysis of channel B intensities from the samples genotyped as AB heterozygotes ($Y'_{n|GT_n=AB}$).

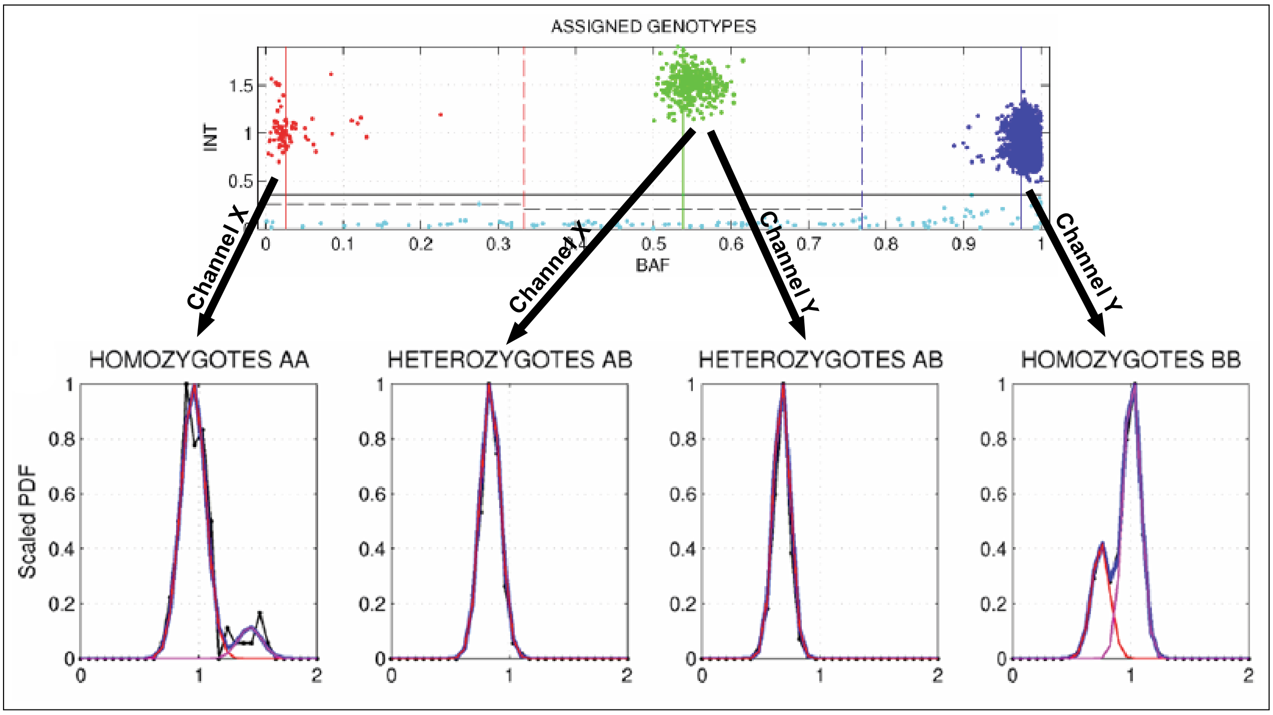

Figure 7: *Genotype and channel independent analysis.* Each SNP genotype cluster is independently analyzed using the intensity channel that carries the information.

As well as dividing the analysis in four independent steps, the algorithm is based on the following assumptions:

- Homozygous deletions (0 copies) are previously detected during the SNP genotyping stage.
- Due to the technical limitations of genotyping microarrays, the intensity measurements show a saturation effect when amplifications are found. For this reason, intensity clustering patterns corresponding to amplifications are very rare and hard to detect unless they span multiple probes.
- Samples categorized as homozygote samples (i.e. AA and BB) can correspond to heterozygous deletions (i.e. A and B) or amplifications (i.e. AA+ and BB+). Due to the saturation effect the algorithm does not stratify amplifications by the number of allele copies.

- Samples characterized as heterozygotes (i.e. AB) can have two or more copies (i.e. AB, AAB, ABB...). The total number of copies can be inferred by independently computing the number of copies of each allele and then adding the results for each sample.

Below we describe the required steps for determining the CNV genotypes using the SNP genotype data and the normalized channel intensities:

Model selection

The algorithm starts by adjusting two probabilistic models to the channel intensities that carry the allele information corresponding to the SNP genotype cluster that is being analyzed. Due to the mentioned saturation effects, it is very uncommon to observe more than two intensity clusters in microarray data and, for this reason, only two models will be fitted to the intensity data: a one-component $\mathcal{P}_1$ and a two-component $\mathcal{P}_2$ Gaussian mixture model (GMM):

- AA homozygotes $\Longrightarrow \mathcal{P}_1(X_n|n \in AA)$ and $\mathcal{P}_2(X_n|n \in AA)$
- BB homozygotes $\Longrightarrow \mathcal{P}_1(Y_n|n \in BB)$ and $\mathcal{P}_2(Y_n|n \in BB)$
- AB heterozygotes $\Longrightarrow \mathcal{P}_1(X_n|n \in AB)$ and $\mathcal{P}_2(X_n|n \in AB)$
- AB heterozygotes $\Longrightarrow \mathcal{P}_1(Y_n|n \in AB)$ and $\mathcal{P}_2(Y_n|n \in AB)$

These two models will be evaluated over the intensity data to identify which fits better. The procedure is detailed for the channel X over the cluster genotype AA but it can be easily extrapolated to other genotypes and channels ($X_n \to Y_n$ y $AA \to AB$ or BB).

The first model is fitted using the mean and the variance of the corresponding intensities:

$$\begin{gathered} \mu_a = \tfrac{1}{N_{AA}} \textstyle\sum_{n \in AA} X'_n \\ \sigma_a^2 = \tfrac{1}{N_{AA}} \textstyle\sum_{n \in AA} (X'_n - \mu_a)^2 \\ X'_{n|n \in AA} \sim \mathcal{P}_1(X) = \mathcal{N}(\mu_a, \sigma_a^2) \end{gathered} \tag{9}$$

where N_{AA} refers to the number of samples genotyped as AA. The second model is fitted using the Expectation-Maximization algorithm (EM):

$$X'_{n|n \in AA} \sim \mathcal{P}_2(X) = \omega_1 \mathcal{N}(\mu_{b_1}, \sigma_{b_1}^2) + \omega_2 \mathcal{N}(\mu_{b_2}, \sigma_{b_2}^2) \tag{10}$$

The initialization of the 2-component model before applying the EM algorithm is crucial to achieve an optimal convergence of the EM algorithm and to reduce the number of iterations required for convergence. The following initialization method (fig. 8A) has been developed to ensure these

results:

$$
\begin{gathered}
\mu_b = \begin{cases} [med(X'_{n|n\in AA}) - \frac{\sigma_a}{4} \quad , \quad med(X'_{n|n\in AA}) + 2\sigma_a], & \text{if } \sum_{n\in AA}(X'_n - med(X'_n)) > 0; \\ [med(X'_{n|n\in AA}) - 2\sigma_a \quad , \quad med(X'_{n|n\in AA}) + \frac{\sigma_a}{4}], & \text{if } \sum_{n\in AA}(X'_n - med(X'_n)) \leq 0; \end{cases} \\
\omega_b = \begin{cases} [\frac{2}{3} \quad , \quad \frac{1}{3}], & \text{if } \sum_{n\in AA}(X'_{n_i} - med(X'_{n_i})) > 0; \\ [\frac{1}{3} \quad , \quad \frac{2}{3}], & \text{if } \sum_{n\in AA}(X'_{n_i} - med(X'_{n_i})) \leq 0; \end{cases} \\
\sigma_b^2 = [0.1\sigma_a^2 \quad , \quad 0.1\sigma_a^2]
\end{gathered} \tag{11}
$$

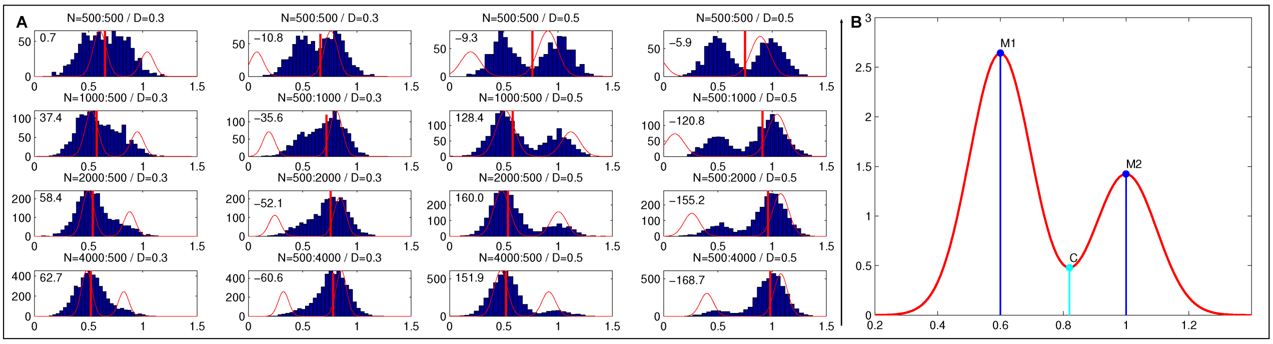

Figure 8: *Model selection.* (A) Shows the two-component GMM initialization within different intensity distributions. (B) Shows how M_1, M_2 and C are computed.

Once fitted the two models, a set of requirements in order to select the second model have been carefully developed and, only if all of them are accomplished, the two-component model (indicating a pattern corresponding to a common CNV) will be selected. Given the 2-component GMM, its probability density function is defined as follows:

$$
\mathcal{P}_2(X) = f_1(X) + f_2(X) = \frac{\omega_1}{\sqrt{2\pi\sigma_{b_1}^2}} \exp\left(\frac{-(x-\mu_{b_1})^2}{2\sigma_{b_1}^2}\right) + \frac{\omega_2}{\sqrt{2\pi\sigma_{b_2}^2}} \exp\left(\frac{-(x-\mu_{b_2})^2}{2\sigma_{b_2}^2}\right) \tag{12}
$$

The X values that maximize each one of the two components (f_1 y f_2) and the X value that minimizes the GMM ($\mathcal{P}_2$) between the two component maximums are defined as follows (fig. 8B):

$$
\begin{aligned}
X_{M_1} &= \underset{X}{\operatorname{argmax}}(f_1(X)) \longrightarrow M_1 = f_1(X_{M_1}) \\
X_{M_2} &= \underset{X}{\operatorname{argmax}}(f_2(X)) \longrightarrow M_2 = f_2(X_{M_2}) \\
X_C &= \underset{X\in(X_{M_1}, X_{M_2})}{\operatorname{argmin}} (\mathcal{P}_2(X)) \longrightarrow C = \mathcal{P}_2(X_C)
\end{aligned} \tag{13}
$$

Once these values are computed, the five requirements that must be accomplished for selecting the 2-component model are:

- $\omega_{b_i} > 0.04\ (0.2)\ ,\ i \in [1, 2]$
- $f_1(X_{M_2}) < M_1$ y $f_2(X_{M_1}) < M_2$

- $\frac{C}{min(M_1,M_2)} < 0.8$ (0.4)
- $\frac{X_{M_1}}{X_{M_2}} < 0.85$
- $\overline{D}_{KL} = \max(\frac{1}{2}(\frac{\sigma^2_{b_1}}{\sigma^2_{b_2}} + \frac{(\mu_{b_2}-\mu_{b_1})^2}{\sigma^2_{b_2}} - 1), \frac{1}{2}(\frac{\sigma^2_{b_2}}{\sigma^2_{b_1}} + \frac{(\mu_{b_1}-\mu_{b_2})^2}{\sigma^2_{b_1}} - 1)) > 2$ (6)

The bracketed values refer to the requirements when analyzing intensities of heterozygote clusters. These values are more restrictive due to their higher variance and to their lower likelihood of having copy number patterns.

Component labeling

Once the models corresponding to each set of intensities and genotypes have been determined, the algorithm must assign a copy number label to each model component. If the one-component model has been selected, only one label will be required, while the two-component model will require two:

$$\begin{aligned}
&\text{AA Homozygotes} \begin{cases} \mathcal{P}_1(X) \text{ selected} \Longrightarrow CN_{AA}(X) \\ \mathcal{P}_2(X) \text{ selected} \Longrightarrow CN^1_{AA}(X) \text{ and } CN^2_{AA}(X) \end{cases} \\
&\text{BB Homozygotes} \begin{cases} \mathcal{P}_1(Y) \text{ selected} \Longrightarrow CN_{BB}(Y) \\ \mathcal{P}_2(Y) \text{ selected} \Longrightarrow CN^1_{BB}(Y) \text{ and } CN^2_{BB}(Y) \end{cases} \\
&\text{AB Heterozygotes} \begin{cases} \mathcal{P}_1(X) \text{ selected} \Longrightarrow CN_{AB}(X) \\ \mathcal{P}_2(X) \text{ selected} \Longrightarrow CN^1_{AB}(X) \text{ and } CN^2_{AB}(X) \\ \mathcal{P}_1(Y) \text{ selected} \Longrightarrow CN_{AB}(Y) \\ \mathcal{P}_2(Y) \text{ selected} \Longrightarrow CN^1_{AB}(Y) \text{ and } CN^2_{AB}(Y) \end{cases}
\end{aligned} \tag{14}$$

This procedure applies different methods depending on the analyzed SNP genotype:

- Homozygotes
  The procedure is detailed for the analysis of channel X intensities over the samples genotyped as AA (equivalent to the analysis of channel Y intensities over the BB samples).
  - $\mathcal{P}_1(X)$ *selected:* Since only one component has been detected, a unique label will be required. The default label assigned to this case is $CN_{AA}(X) = 2$ (the common diploid state) unless a high number of homozygous deletions have been detected. In this case, the assigned label will be $CN_{AA}(X) = 1$ to ensure Hardy-Weinberg equilibrium ($f(--) > f(A-) > f(AA)$).
  - $\mathcal{P}_2(X)$ *selected:* Since two components have been detected, two labels will be required and the weights of each component (ω_1 and ω_2) are expected to be proportional to the frequencies of each group. Labels are assigned to ensure Hardy-Weinberg equilibrium

(fig. 9):

$$[CN^1_{AA}(X)\ ,\ CN^2_{AA}(X)] = \begin{cases} [1\ ,\ 2)], & \text{if } \omega_1 < \omega_2;\ \text{(fig. 9A)} \\ [1\ ,\ 2], & \text{if } \omega_1 \geq \omega_2 \text{ and } f(--)\uparrow;\ \text{(fig. 9B)} \\ [2\ ,\ 3], & \text{if } \omega_1 \geq \omega_2 \text{ and } f(--)\downarrow;\ \text{(fig. 9C)} \end{cases} \tag{15}$$

- *Heterozygotes*
  When analyzing heterozygotes the common state is AB, which means one copy per intensity channel. Then, when $\mathcal{P}_1(X)$ or $\mathcal{P}_1(Y)$ are selected the default labels are $CN_X = 1$ or $CN_Y = 1$. When $\mathcal{P}_2(X)$ or $\mathcal{P}_2(Y)$ are selected the default labels are $[CN^1_{AB}(X)\ ,\ CN^2_{AB}(X)] = [1, 2]$ o $[CN^1_{AB}(Y)\ ,\ CN^2_{AB}(Y)] = [1, 2]$.

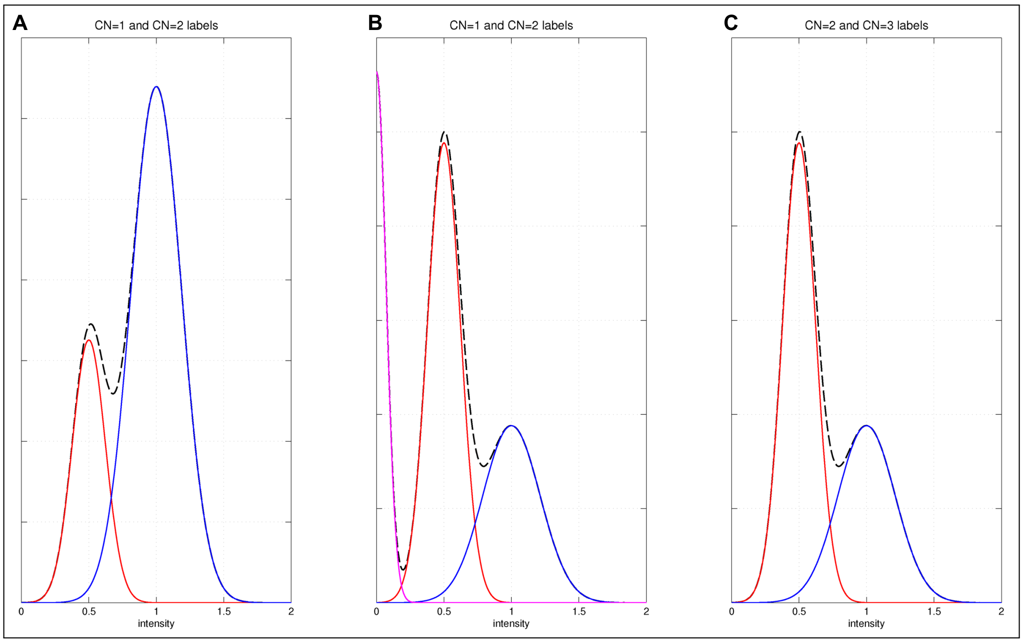

Figure 9: *Component labeling.* These figures show three examples of how labels are assigned to ensure Hardy-Weinberg equilibrium.

Scoring

Sample scores will be assigned depending on their genotypes and their intensity likelihood relative to each model component. Depending on the SNP genotype scores will be computed as follows (fig. 10):

- AA Homozygotes:
  - $\mathcal{P}_1(X)$: If the one-component model has been selected, the algorithm assigns to each

sample an score proportional to its deviation with respect to the component mean:

$$SC_n = \begin{cases} CN_{AA}(X) + \frac{X_n - \mu_a}{8\sigma_a}, & \text{si } |\frac{X_n - \mu_a}{\sigma_a}| < 8; \\ CN_{AA}(X) + 1, & \text{si } \frac{X_n - \mu_a}{\sigma_a} > 8; \\ CN_{AA}(X) - 1, & \text{si } \frac{X_n - \mu_a}{\sigma_a} < -8; \end{cases} \tag{16}$$

– $\mathcal{P}_2(X)$: If the two-component model has been selected, each sample is scored depending on its intensity and its relative position with respect to the mean of each component (μ_{b_1} and μ_{b_2}):

$$SC_n = \begin{cases} CN^1_{AA}(X) - 1, & \text{si } X_n < \mu_{b_1} - \frac{8\sigma}{b_1}; \\ CN^1_{AA}(X) + \frac{X_n - \mu_{b_1}}{8\sigma_{b_1}}, & \text{si } \mu_{b_1} - \frac{8\sigma}{b_1} \leq X_n < \mu_{b_1}; \\ \frac{f_1(X_n)}{f_1(X_n)+f_2(X_n)} CN^1_{AA}(X) + \frac{f_2(X_n)}{f_1(X_n)+f_2(X_n)} CN^2_{AA}(X), & \text{si } \mu_{b_1} \leq X_n \leq \mu_{b_2}; \\ CN^2_{AA}(X) + \frac{X_n - \mu_{b_2}}{8\sigma_{b_2}}, & \text{si } \mu_{b_2} \leq X_n < \mu_{b_2} + \frac{8\sigma}{b_2}; \\ CN^2_{AA}(X) + 1, & \text{si } X_n > \mu_{b_2} + \frac{8\sigma}{b_2}; \end{cases} \tag{17}$$

- BB Homozygotes:
  Same than previous but replacing: $AA \to BB$, $X \to Y$ and $X_n \to Y_n$.
- AB Heterozygotes:
  Para $W \in [X, Y]$:
  – $\mathcal{P}_1(W)$: If the one-component model has been selected, the algorithm assigns the same score to all the samples corresponding to the component label $CN_{AB}(W)$.

$$SC^W_n = CN_{AB}(W) \ , \ \forall n \in AB \tag{18}$$

  – $\mathcal{P}_2(X)$: If the two-component model is selected, each sample is scored depending on its intensity and its relative position with respect to the mean of each component (μ_{b_1} and μ_{b_2}):

$$SC^W_n = \begin{cases} CN^1_{AB}(Z), & \text{si } Z_n < \mu_{b_1}; \\ \frac{f_1(Z_n)}{f_1(Z_n)+f_2(Z_n)} CN^1_{AB}(Z) + \frac{f_2(Z_n)}{f_1(Z_n)+f_2(Z_n)} CN^2_{AB}(Z), & \text{si } \mu_{b_1} \leq Z_n \leq \mu_{b_2}; \\ CN^2_{AB}(Z), & \text{si } \mu_{b_2} \leq Z_n; \end{cases} \tag{19}$$

The final score of heterozygote samples will be computed as: $SC_n = SC^X_n + SC^Y_n$

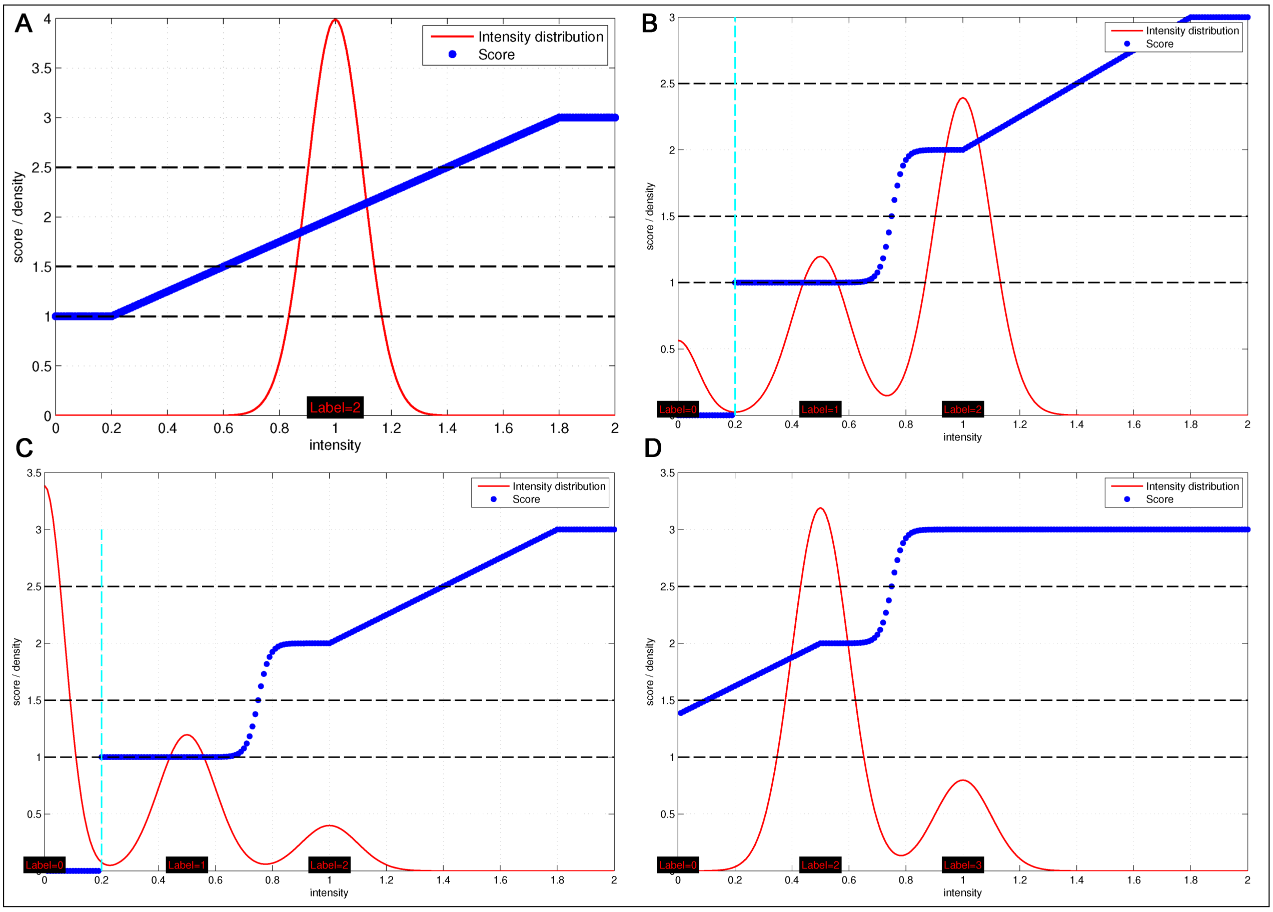

Figure 10: *Scoring.* These figures show how the scores are assigned depending on the labels and the number of components of the selected model (red line). Blue points show the relationship between sample intensities (horizontal axis) and sample scores (vertical axis). The vertical cyan line represents T_0, the 0-copies intensity threshold computed in the SNP genotyping stage, while the horizontal black-dotted lines represent the limits between discrete copy number assignments.
